# CDK1 and CCNB1 as potential diagnostic markers of rhabdomyosarcoma: validation following bioinformatics analysis

**DOI:** 10.1186/s12920-019-0645-x

**Published:** 2019-12-23

**Authors:** Qianru Li, Liang Zhang, Jinfang Jiang, Yangyang Zhang, Xiaomeng Wang, Qiaochu Zhang, Yang Wang, Chunxia Liu, Feng Li

**Affiliations:** 1 Department of Pathology, Shihezi University School of Medicine and The Key Laboratories for Xinjiang Endemic and Ethnic Diseases, Chinese Ministry of Education, Shihezi, Xinjiang, 832002 China; 2 Department of Anesthesiology, Peking Union Medical College Hospital, Chinese Academy of Medical Sciences and Peking Union Medical College, Beijing, 100730 People’s Republic of China; 3 Department of Pathology and Medical Research Center, Beijing Chaoyang Hospital, Capital Medical University, Beijing, 100020 People’s Republic of China

**Keywords:** Rhabdomyosarcoma, Hub genes, Diagnosis, CDK1, CCNB1, Bioinformatics analysis

## Abstract

**Background:**

Rhabdomyosarcoma (RMS), a common soft-tissue malignancy in pediatrics, presents high invasiveness and mortality. However, besides known changes in the PAX3/7-FOXO1 fusion gene in alveolar RMS, the molecular mechanisms of the disease remain incompletely understood. The purpose of the study is to recognize potential biomarkers related with RMS and analyse their molecular mechanism, diagnosis and prognostic significance.

**Methods:**

The Gene Expression Omnibus was used to search the RMS and normal striated muscle data sets. Differentially expressed genes (DEGs) were filtered using R software. The DAVID has become accustomed to performing functional annotations and pathway analysis on DEGs.

The protein interaction was constructed and further processed by the STRING tool and Cytoscape software. Kaplan–Meier was used to estimate the effect of hub genes on the ending of sarcoma sufferers, and the expression of these genes in RMS was proved by real-time polymerase chain reaction (RT-PCR). Finally, the expression of CDK1 and CCNB1 in RMS was validated by immunohistochemistry (IHC).

**Results:**

A total of 1932 DEGs were obtained, amongst which 1505 were up-regulated and 427were down-regulated. Up-regulated genes were largely enriched in the cell cycle, ECM-receptor interaction, PI3K/Akt and p53 pathways, whilst down-regulated genes were primarily enriched in the muscle contraction process. CDK1, CCNB1, CDC20, CCNB2, AURKB, MAD2L1, HIST2H2BE, CENPE, KIF2C and PCNA were identified as hub genes by Cytoscape analyses. Survival analysis showed that, except for HIST2H2BE, the other hub genes were highly expressed and related to poor prognosis in sarcoma. RT-PCR validation showed that CDK1, CCNB1, CDC20, CENPE and HIST2H2BE were significantly differential expression in RMS compared to the normal control. IHC revealed that the expression of CDK1 (28/32, 87.5%) and CCNB1 (26/32, 81.25%) were notably higher in RMS than normal controls (1/9, 11.1%; 0/9, 0%). Moreover, the CCNB1 was associated with the age and location of the patient’s onset.

**Conclusions:**

These results show that these hub genes, especially CDK1 and CCNB1, may be potential diagnostic biomarkers for RMS and provide a new perspective for the pathogenesis of RMS.

## Background

Rhabdomyosarcoma (RMS) is a common pediatric malignant tumour [1] that can be categorised into four main histological types, namely, embryonal RMS (ERMS), alveolar RMS (ARMS), pleomorphic RMS (PRMS) and spindle cell/sclerosing RMS (SRMS); amongst these subtypes, ERMS and ARMS are the two most important. Approximately 80% of all ARMS contains the PAX3/7-FOXO1 gene fusion, and this fusion-positive gene was identified as a prognostic predictor in RMS [2–6]. Other prognostic (e.g. MYOD1 mutation, RAS pathway mutation, NCOA2 or VGLL2 gene fusion) and diagnostic (e.g. intermediate silk proteins [desmin, vimentin and nestin], muscle proteins [myoglobin and actin] and myocyte determinant-coding genes [MYOD1 and myogenin]) markers have also been found in RMS [3, 7]. Although these biomarkers play a considerable role in RMS, the 5-year survival rate of RMS sufferer is still lower than 20% [4–6]. Therefore, more efforts are needed to explore the development of RMS and determine the diagnosis and prognostic biomarkers of the disease.

Microarray analysis has recently received increased attention in the field of medical oncology [8]. The technology can not only find differences in genetic and epigenetic phenotypes caused by tumours but also evaluate markers for disease diagnosis and therapy [8]. In this study, we analysed differentially expressed genes (DEGs) among RMS and ordinary striated muscle specimens retrieved from database and identified 10 hub genes. Real-time polymerase chain reaction (RT-PCR) revealed that the expression trend of the 10 hub genes were consistent with the sequencing results. The cell cycle-related molecules CDK1 and CCNB1 are the most connected hub genes, and these genes have been reported to be potential diagnostic or prognostic markers in a variety of tumours, such as glioblastoma malignancies [9], hepatocellular carcinoma [10, 11] and non-muscle invasive bladder cancer [12]. However, the clinical role of CDK1 and CCNB1 in RMS has not been clarified. Therefore, the study used immunohistochemistry (IHC) to further verify that more than 80% of RMS clinical samples showed increased expression of CDK1 and CCNB1 protein levels, and the CCNB1 gene was associated with the age and location of RMS patients.

## Methods

### Procurement of raw data

The required original data were gained from Gene Expression Omnibus (GEO) database (www.ncbi.nlm.nih.gov/geo/). We screened 82 samples, including 66 samples of RMS patient tissues (GSE16382 [*N* = 8] [13] and GSE66533 [*N* = 58) [14]) and 16 samples of normal striated muscle tissues (GSE39454 [*N* = 5] [15], GSE17674 [*N* = 5] [16] and GSE38417 [*N* = 6]). All data were obtained on the GPL570 platform to ensure consistent data processing.

### Analysis of DEGs by R software

Pre-processing and standardisation of the original dataset were the basis for obtaining accurate data. The matrix was constructed by the R language (https://www.r-project.org/) Affy package and the Affymetrix gene expression microarray data-CEL format data was read. The absolute value of | log2 fold change (FC) | was set to > 2, and the cutoff value of *P* < 0.01 was considered valid. Screening for FDR and *P* values to obtain all DEGs. The datasets generated during the current study are available in the GEO Database Series GSE141690 repository. R software can also regroup, integrate and compare DEGs according to experimental requirements. In this experiment, ggplot2 and the pheatmap package were used to plot the volcano diagrams and pheatmap of the DEGs, respectively.

### Enrichment analysis of DEGs

The annotated, visualised and imploded database DAVID (https://david.ncifcrf.gov/) is a repository for integrating biological information. This database allows online analysis of a variety of biological functions and can be used to obtain specific genes for specific pathways [17]. Gene ontology (GO) analysis, including cell composition (CC), biological processes (BP) and molecular functions (MF), was used to find the function of DEGs. The Kyoto Encyclopedia of Genes and Genomes (KEGG) was selected to screen for pathway enrichment of DEGs. A *P* value of < 0.05 was considered effective.

### Manufacture of the protein interaction network and module production

We used the STRING (http://www.string-db.org/) [18] to explore relationships between DEGs. This database not only intuitively reflects the network map but also presents a tabular form of genetic relationships. We entered all DEG names to obtain protein–protein interaction (PPI) information. A high confidence score of > 0.9 was considered significant. We used the plug-ins CytoHubba and MCODE [19] of Cytoscape [20] to filter hub genes and important modules in the network to obtain high-connectivity genes and functional enrichment.

### Survival analysis of the hub genes

The Kaplan–Meier (KM) plotter (http://kmplot.com/) is a network tool that integrates databases such as GEO, EGA and TCGA to detect patient survival [21]. We selected the median of the hub gene to classify patients into high and low expression groups to compare the overall survival of the patients.

### Collection of human tissue specimens

A total of 32 paraffin-embedded RMS specimens (7 ARMS, 20 ERMS, 2 PRMS, 1 SRMS and 2 unclassified RMS) and 9 normal striated muscle samples were obtained from Xinjiang Medical University and Shihezi University Hospital, China. This research has been endorsed by the hospital ethics committee and is based on the ethical requirements of the Helsinki Declaration. All participants have the right to know.

### Detection of the mRNA expression of the hub genes by RT-PCR

Total RNA was collected from paraffin-embedded RMS and normal striated muscle tissues using RNeasy FFPE kit (Cat No.73504, QIAGEN, Germany) according to the instructions. RNA samples were reverse transcribed into cDNA by SuperQuick Real-Time Master Mix (Catalog No. CW2391M, China Chagan) reagent. RT-PCR analysis was performed with UltraSYBR Mixture (Low ROX) (Cat no. CW2601M, Qiagen, China) on the 7500 RT- PCR System. All primers were synthesised by Sangon Biotech and shown in Additional file 1: Table S1. The experiment was repeated in triplicate. The relative value of the hub gene was normalised to those of GAPDH through the 2^-ΔΔCt^ calculation.

### IHC staining of CDK1 and CCNB1 genes

RMS and normal samples were immunostained using the EnVision system (DAKO, Carpinteria, CA).

Slides with lengths of 1–2 mm were deparaffinised, heat repaired with EDTA antigen repair solution (pH = 9.0) and incubated with anti-CDK1 rabbit monoclonal antibody (EPR165 [ab133327]; 1:1000 dilution; Abcam) and anti-Cyclin B1 rabbit monoclonal antibody (Y106 [ab32053]; 1:250 dilution; Abcam). Tissue colour was visualised by staining with diaminobenzidine. The slides were counterstained with haematoxylin and dehydrated. The staining results of CDK1 were evaluated by the sum of staining intensity and degree (0–5 points). The positive percentage of tumour cells was divided into 3 levels (0 [0%], 1 [1–50%] and 2 [51–100%]), and staining intensity was differentiated into 4 ranks (0 (absent), 1 (inferior), 2 (medium) and 3 (marked)). Scores > 3 indicate high expression levels, whilst scores ≤2 indicate low expression levels [22]. Tonsil tissues were used as the positive control. The staining results of CCNB1 were evaluated in terms of staining intensity and extent (0–9 points). Scores > 3 indicate high expression levels, whilst scores ≤3 indicate low expression levels. Degree of staining was divided into four levels based on the positive percentage of tumour cells (0, absence; 1, < 10%; 2, 10–50%;3, > 50%). Degree of dyeing was also divided into 4 grades (no signal, 0; inferior, 1; medium, 2; marked, 3) [23]. Cervical cancer tissue was applied to the positive reference.

### Statistical analysis

Data were processed by the Statistical Package for the Social Science version 20.0. IHC results of CDK1 and CCNB1 were accurately detected using χ2 detection or Fisher’s exact detection and their relationship to clinical data was assessed. All experimental data of RT-PCR were analyzed using GraghPad Prism 7.0. The expression level of each hub gene was expressed as a fold change using the 2^-ΔΔC^ method. *P* < 0.05 considered significant.

## Results

### DEGs from RMS and normal striated muscle tissues

**Fig. 1:**
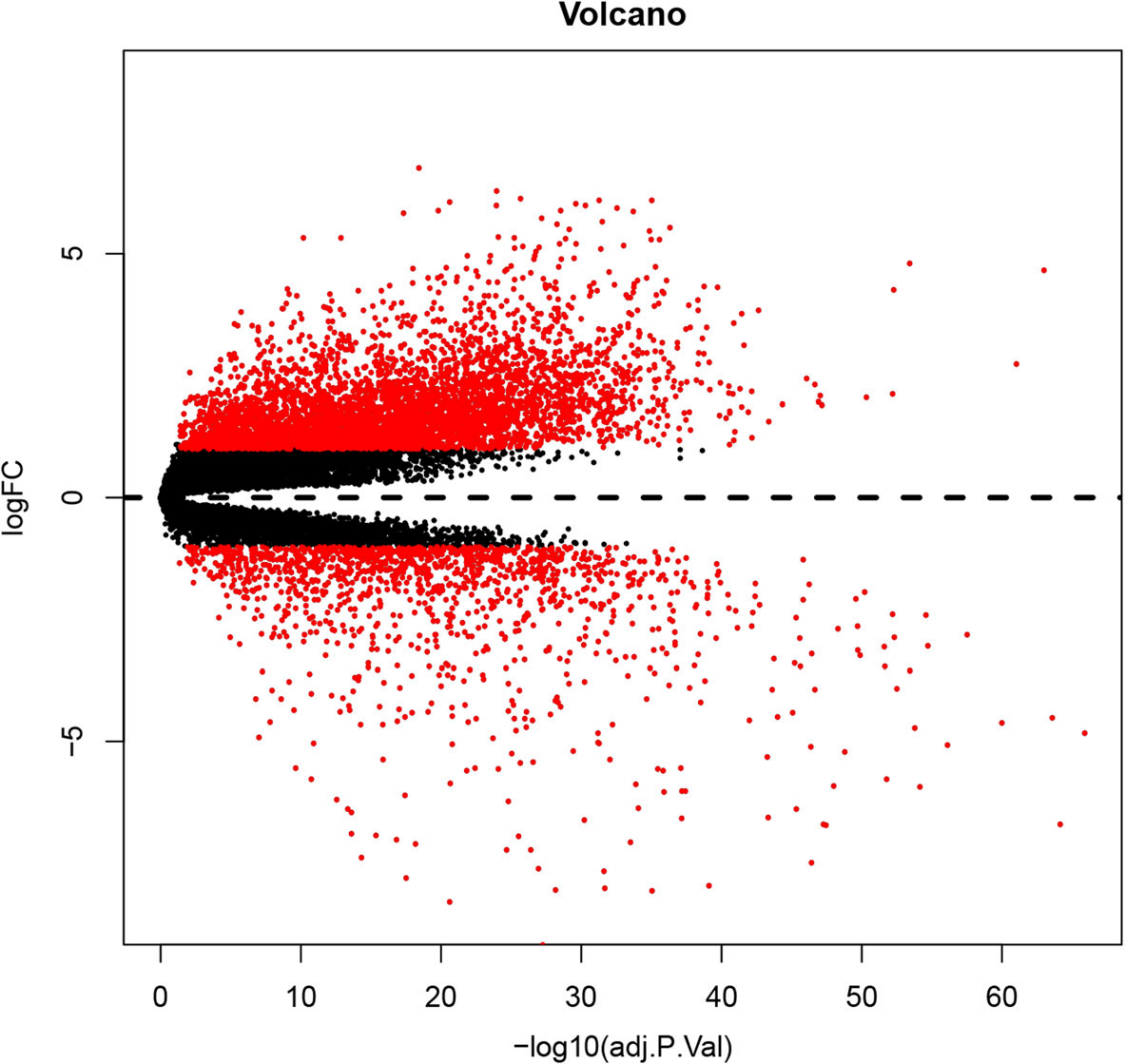
Volcanic diagram of DEGs. Red represents DEGs with fold change > 2, and black represents DEGs with fold change < 2

The study found 1932 genes (1505 up-regulated and 427 down-regulated genes) from RMS samples and normal striated muscle samples (*P* < 0.01 and | log2 FC | ≥ 2; Additional file 2: Table S2). All DEG volcano diagrams are shown in Fig. 1.

### Functional enrichment of DEGs

To understand the changes in DEGs function, we mapped all of the genes to the DAVID database. GO functional analysis of RMS and normal striated muscle DEGs revealed that up-regulated genes in BP are largely enriched in cell or nuclear division, G2/M transition in cell cycle and transcription. The DEGs were abundantly involved in CC, including nucleus, nucleoplasm, membrane and cytoplasm, and MF, including protein binding, DNA binding, nucleic acid binding and poly (A) RNA binding. Down-regulated DEGs in BP were mainly involved in muscle filament sliding, muscle contraction, cardiac muscle contraction, sarcomere organisation and muscle organ development. The genes were mainly involved in CC, including Z disc, M band, sarcomere and actin cytoskeleton, and MF, including structural constituent of muscles, actin binding, actin filament binding and calmodulin binding (Table 1). Functional analysis showed that up-regulated genes mainly affect the growth of tumour cell whereas down-regulated genes are mainly participated in muscle contraction.

**Table 1:** Gene ontology analysis of differentially expressed genes associated with RMS

Expression	Category	Term/gene function	Gene count	%	*P* Value
Up-regulated	GOTERM_BP_DIRECT	GO:0006351~transcription, DNA-templated	193	13.23731	1.13E-05
	GOTERM_BP_DIRECT	GO:0006355~regulation of transcription, DNA-templated	160	10.97394	1.19E-06
	GOTERM_BP_DIRECT	GO:0051301~cell division	104	7.133059	4.72E-36
	GOTERM_BP_DIRECT	GO:0007067~mitotic nuclear division	65	4.458162	1.67E-19
	GOTERM_BP_DIRECT	GO:0000086~G2/M transition of mitotic cell cycle	40	2.743484	7.88E-14
	GOTERM_CC_DIRECT	GO:0005634~nucleus	533	36.55693	1.64E-19
	GOTERM_CC_DIRECT	GO:0005737~cytoplasm	462	31.68724	2.38E-08
	GOTERM_CC_DIRECT	GO:0005654~nucleoplasm	316	21.67353	7.16E-19
	GOTERM_CC_DIRECT	GO:0005829~cytosol	287	19.6845	1.93E-04
	GOTERM_CC_DIRECT	GO:0016020~membrane	208	14.26612	1.29E-05
	GOTERM_MF_DIRECT	GO:0005515~protein binding	763	52.33196	8.43E-14
	GOTERM_MF_DIRECT	GO:0046872~metal ion binding	201	13.78601	8.80E-06
	GOTERM_MF_DIRECT	GO:0003677~DNA binding	187	12.82579	1.09E-09
	GOTERM_MF_DIRECT	GO:0003676~nucleic acid binding	122	8.367627	5.09E-09
	GOTERM_MF_DIRECT	GO:0044822~poly(A) RNA binding	108	7.407407	0.002521
Down-regulated	GOTERM_BP_DIRECT	GO:0006936~muscle contraction	33	7.875895	4.20E-28
	GOTERM_BP_DIRECT	GO:0030049~muscle filament sliding	25	5.966587	3.00E-31
	GOTERM_BP_DIRECT	GO:0060048~cardiac muscle contraction	18	4.295943	1.81E-17
	GOTERM_BP_DIRECT	GO:0045214~sarcomere organization	14	3.341289	8.14E-15
	GOTERM_BP_DIRECT	GO:0007517~muscle organ development	14	3.341289	5.27E-08
	GOTERM_CC_DIRECT	GO:0005829~cytosol	105	25.05967	1.97E-06
	GOTERM_CC_DIRECT	GO:0030018~Z disc	42	10.02387	1.26E-39
	GOTERM_CC_DIRECT	GO:0015629~actin cytoskeleton	21	5.011933	2.35E-08
	GOTERM_CC_DIRECT	GO:0030017~sarcomere	17	4.057279	1.58E-17
	GOTERM_CC_DIRECT	GO:0031430~M band	16	3.818616	1.41E-20
	GOTERM_MF_DIRECT	GO:0003779~actin binding	39	9.307876	3.74E-20
	GOTERM_MF_DIRECT	GO:0042803~protein homodimerization activity	29	6.921241	0.001951
	GOTERM_MF_DIRECT	GO:0008307~structural constituent of muscle	24	5.727924	5.18E-28
	GOTERM_MF_DIRECT	GO:0005516~calmodulin binding	18	4.295943	6.18E-07
	GOTERM_MF_DIRECT	GO:0051015~actin filament binding	16	3.818616	1.39E-07

*GO* Gene Ontology, *BP* Biological process, *CC* Cell Component, *MF* Molecular function

### Pathway enrichment analysis of DEGs

We uploaded all DEGs to the KEGG pathway of the DAVID database and analysed the resulting gene enrichment pathways. Up-regulated genes (containing 23 enrichment pathways) were abundantly enriched in the cell cycle, p53, ECM-receptor interaction, cancer and viral cancerous pathways, whilst down-regulated genes (containing 31 enrichment pathways) were mainly enriched in the adrenergic cardiomyocyte, myocardial contraction and calcium signalling pathways (Table 2).

**Table 2:** KEGG pathway analysis of differentially expressed genes associated with RMS

Pathway ID	Name	Count	%	*P* Value	Genes
Up-regulated DEGs					
hsa04110	Cell cycle	38	2.60631	3.62E-15	E2F3, E2F5, DBF4, TTK, CHEK1, CHEK2, PTTG1, CCNE2, CDC45, ORC6, CCNA2, CDC7, CDK1, CDC6, ANAPC4, SKP2, CDC23, ESPL1, CDC20, CDK6, MCM2, ATR, MCM3, CDK4, MCM5, SMC3, CDC25B, CCNB1, CCND1, HDAC2, CCNB2, MAD2L1, HDAC1, CCND2, PCNA, YWHAQ, BUB1B, GADD45A
hsa04115	p53 signaling pathway	22	1.508916	1.41E-09	CDK1, ZMAT3, CHEK1, CDK6, ATR, CHEK2, PMAIP1, CDK4, CCNG2, GTSE1, CCNE2, CCNB1, TP53I3, CASP3, CCND1, CCNB2, CCND2, RRM2, BAX, DDB2, IGFBP3, GADD45A
hsa04512	ECM-receptor interaction	22	1.508916	2.27E-07	COL4A2, COL4A1, TNC, COL3A1, COL2A1, ITGA4, COL5A2, COL5A1, HMMR, CD47, LAMA1, SDC1, LAMA5, COL6A3, COL1A2, COL1A1, SV2A, LAMB1, THBS2, COL11A1, SPP1, FN1
hsa05200	Pathways in cancer	42	2.880658	0.003722	WNT5A, CKS1B, E2F3, GNAI3, GNAI1, PGF, FGF9, STK36, LPAR4, TCF7L2, EDNRA, CCNE2, FOS, CASP3, CXCR4, LAMB1, TRAF5, FN1, COL4A2, CTBP2, COL4A1, MSH2, SKP2, BRCA2, CDK6, BIRC5, FZD2, CDK4, DAPK1, LAMA1, NRAS, CCND1, HSP90B1, HIF1A, HDAC2, HDAC1, LAMA5, GNB1, BAX, CKS2, GNB4, F2R
hsa05203	Viral carcinogenesis	26	1.783265	0.003088	CDK1, EGR3, EGR2, SKP2, GTF2H3, CDC20, CHEK1, CDK6, PMAIP1, CDK4, SCRIB, CCNE2, NRAS, CASP3, CCND1, HDAC2, HDAC1, CCND2, SND1, BAX, YWHAQ, RBPJ, TRAF5, HIST1H4J, CCNA2, RASA2
Down-regulated DEGs					
hsa04261	Adrenergic signaling in cardiomyocytes	24	5.727924	2.18E-13	ATP1B1, CACNA2D1, PPP2R3A, SCN1B, MYL2, MYL3, TNNC1, ATP1B4, CACNB1, MYH7, MYH6, ATP1A2, TPM2, CACNG1, TNNI3, CACNA1S, TPM3, ADRB2, MAPK12, PLN, PPP1R1A, CAMK2B, CALML6, CAMK2A
hsa04260	Cardiac muscle contraction	17	4.057279	1.03E-11	ATP1B1, CACNA2D1, MYL2, COX7A1, MYL3, TNNC1, ATP1B4, CACNB1, MYH7, ATP1A2, MYH6, CACNG1, TNNI3, TPM2, CACNA1S, TPM3, COX6A2
hsa05410	Hypertrophic cardiomyopathy (HCM)	13	3.102625	2.09E-07	CACNA2D1, MYL2, MYL3, TNNC1, CACNB1, CACNG1, TNNI3, TTN, TPM2, CACNA1S, TPM3, SGCG, PRKAA2
hsa05414	Dilated cardiomyopathy	13	3.102625	4.81E-07	CACNA2D1, MYL2, MYL3, TNNC1, CACNB1, CACNG1, TNNI3, TTN, TPM2, CACNA1S, TPM3, SGCG, PLN
hsa04020	Calcium signaling pathway	18	4.295943	8.11E-07	TNNC2, SLC25A4, TNNC1, PHKG1, MYLK4, PHKA1, MYLK2, CACNA1S, VDAC1, GNAL, ADRB2, PLN, ATP2A1, RYR1, PLCD4, CAMK2B, CALML6, CAMK2A

### Network construction and module analysis of PPI

**Fig. 2:**
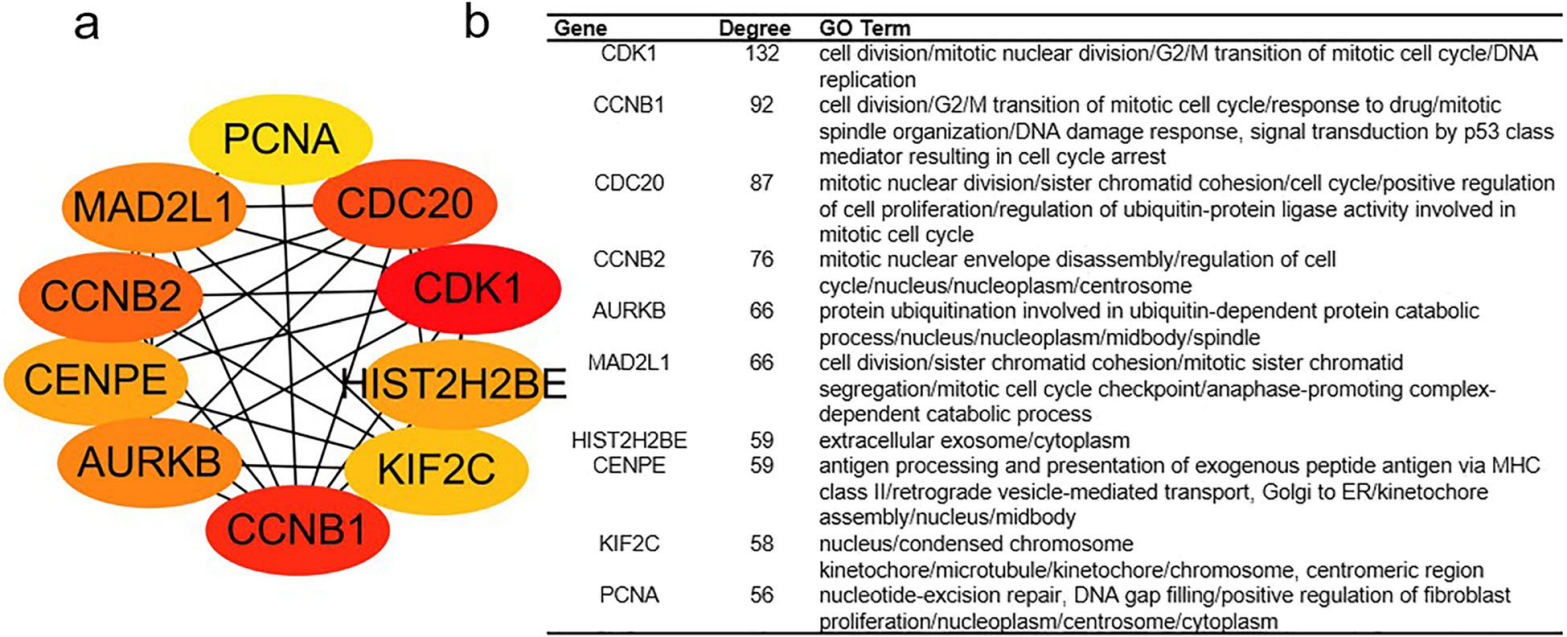
Top 10 hub genes identified in CytoHubba plug-in for DEGs from RMS and normal specimens. **a** Red indicates high enrichment, and yellow indicates low enrichment. **b** Degree and GO term of the hub genes

**Fig. 3:**
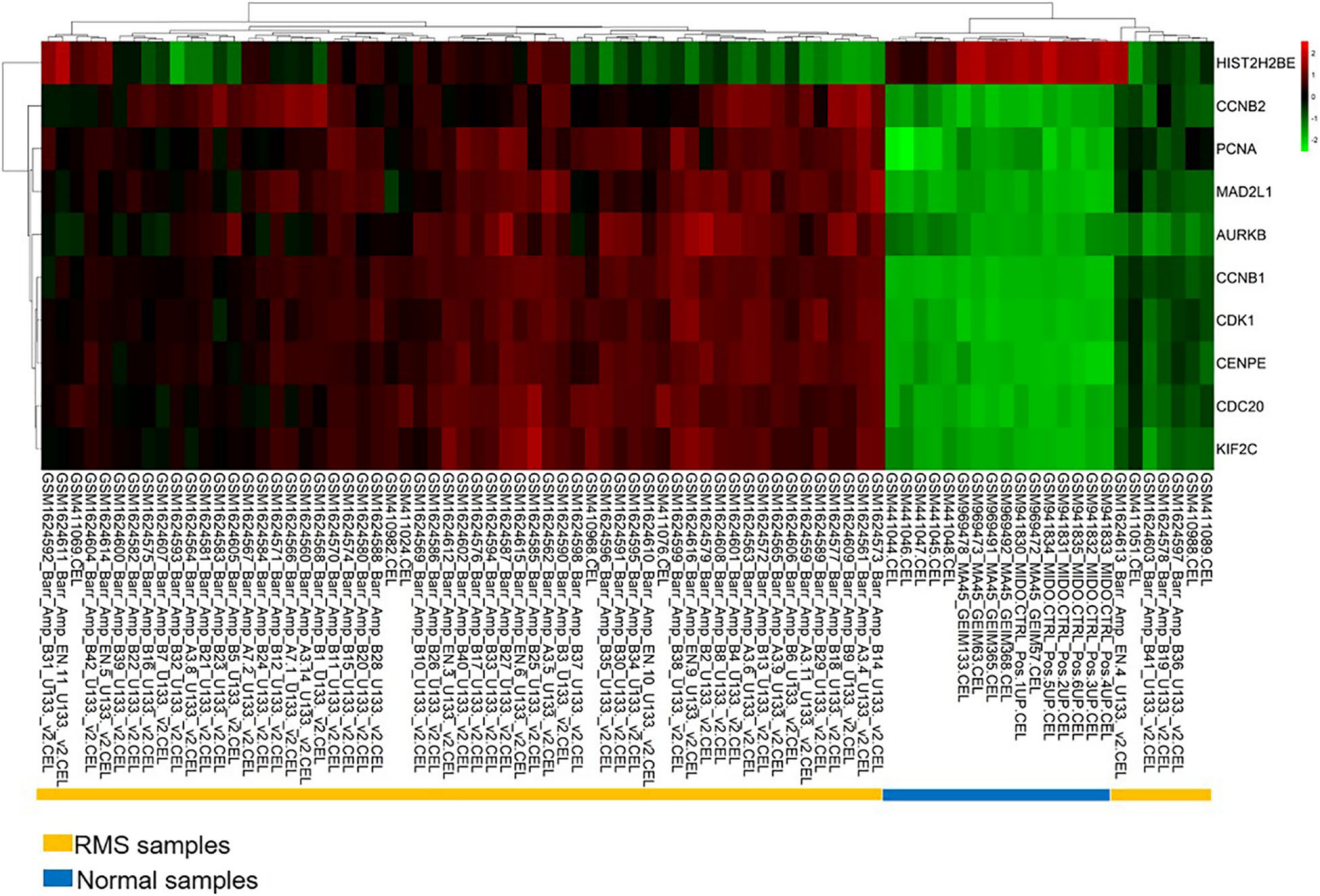
Pheatmap of the 10 hub genes. Red: up-regulation; Green: down-regulation

**Fig. 4:**
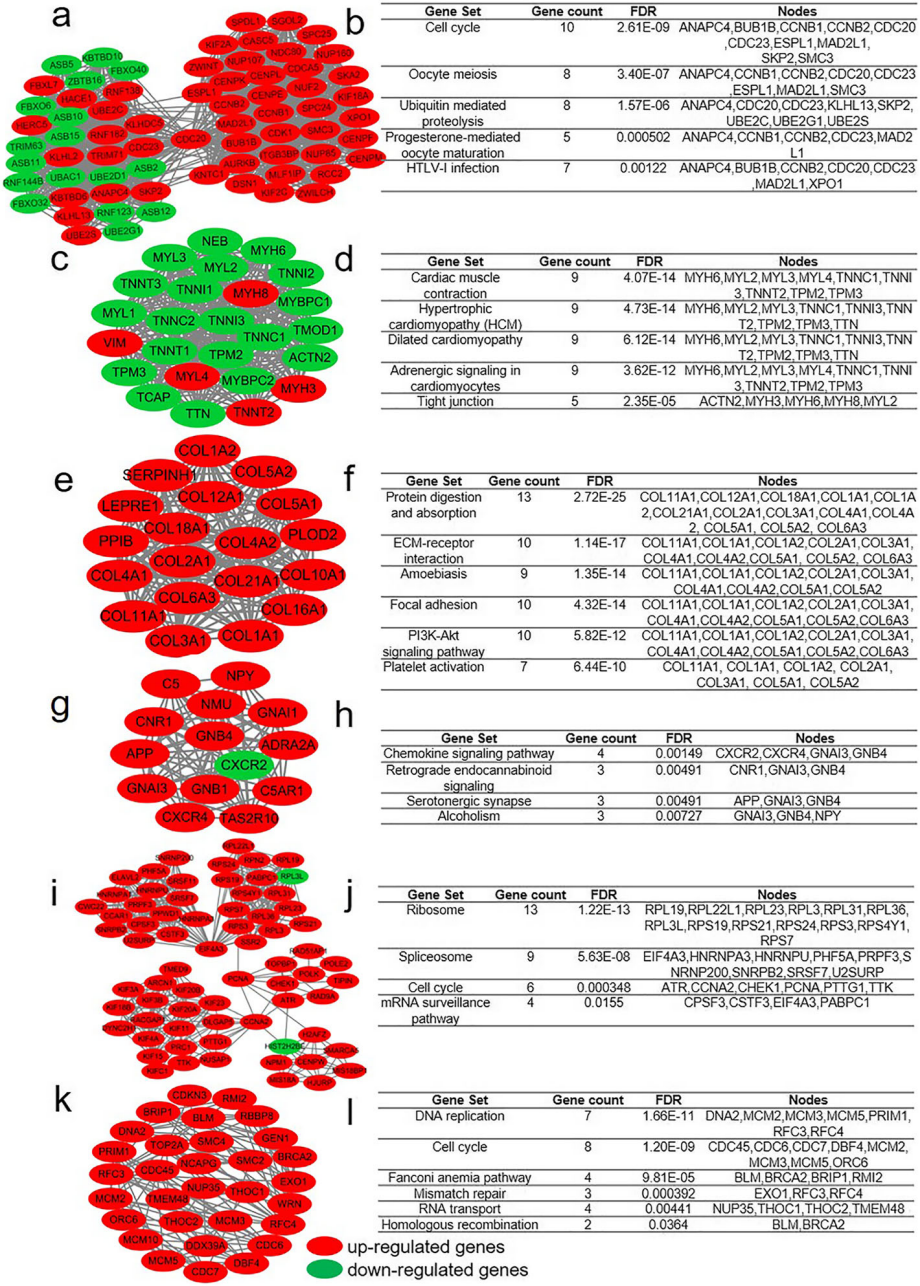
Top 6 modules with enrichment pathways identified from PPI of DEGs. **a** Module 1; **b** Enrichment of Module 1; **c** Module 2; **d** Enrichment of Module 2; **e** Module 3; **f** Enrichment of Module 3; **g** Module 4; **h** Enrichment of Module 4; **i** Module 5; **j** Enrichment of Module 5; **k** Module 6; **l** Enrichment of Module 6. Red nodes were up-regulated genes, green nodes were down-regulated genes, and gray nodes were interactions between nodes

The protein relationship amongst the DEGs was analyzed using the STRING tool. We selected a confidence score greater than 0.9 to build a PPI network for DEGs analysis in RMS and normal controls. One thousand seven hundred eighty-four nodes and 5067 eDEGs are displayed in PPI (Additional file 3: Figure S1). The greater the connection value of the node and the higher the degree of network connectivity, the greater the extent to which the disease occurs. Therefore, we screened the top 10 hub genes (degree > 55) using the cytoHubba plug-in for Cytoscape (Fig. 2a) and analysed their functional enrichment (Fig. 2b). Hub genes were abundantly involved in cell cycle, p53 signalling and mismatch repair paths. Fig. 3 shows the pheatmap of the hub gene in RMS and normal striated muscle. Amongst these genes, HIST2H2BE was down-regulated whereas the rest were up-regulated in RMS. To obtain highly enriched modules, we selected the MCODE plug-in for Cytoscape for high-cluster screening. The first seven central modules were selected. They are connected nodes > 31 and node scores > 9 (Fig. 4). The modules obtained were abundantly concentrated in the cell cycle, biological and molecular synthesis, ECM-receptor interaction, amoebiasis, PI3K-Akt signalling, chemokine signalling, DNA repair and other pathways. Central module 3 revealed no enrichment pathway.

### Survival analysis

**Fig. 5:**
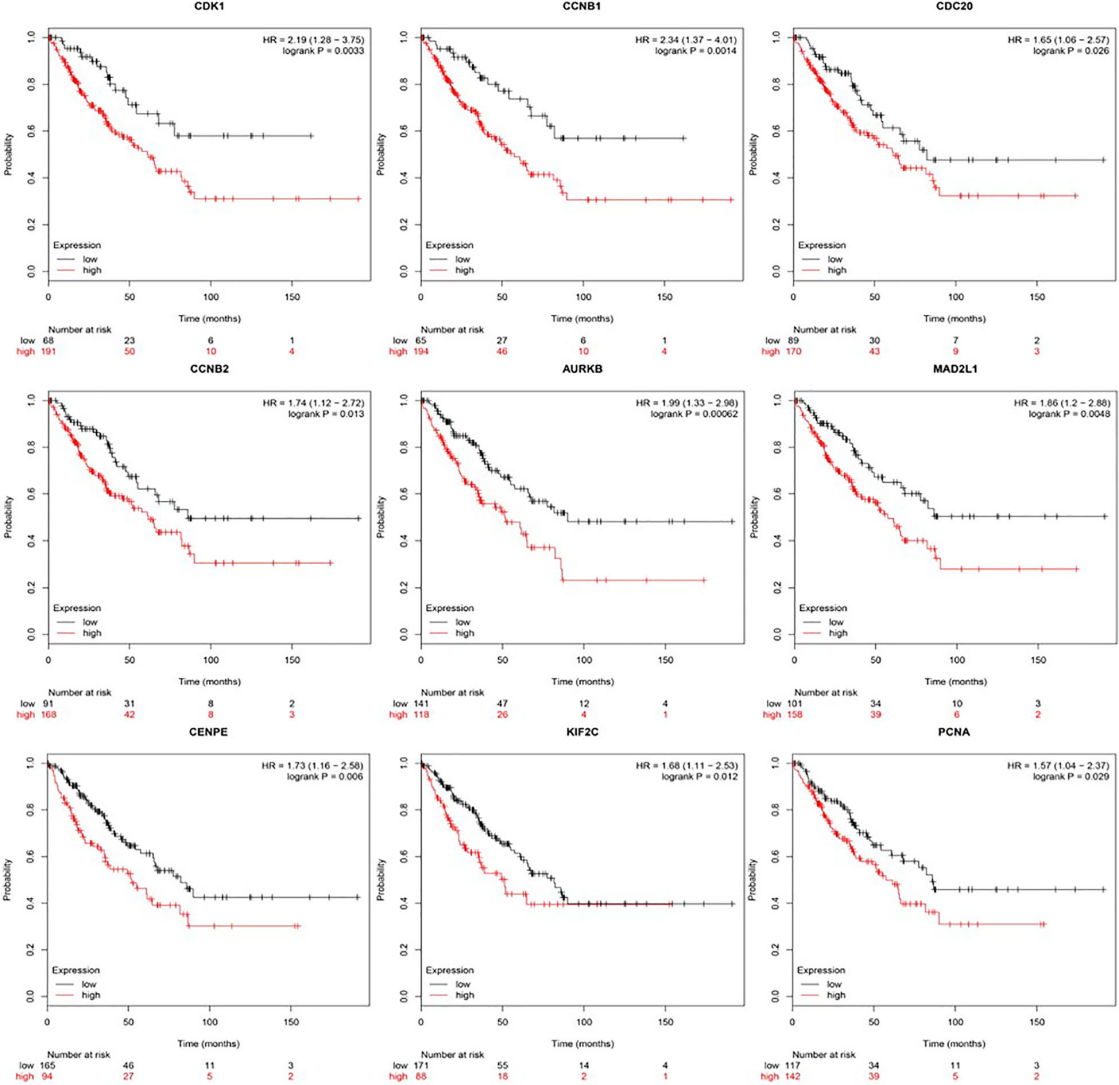
Kaplan–Meier plotter of 9 hub genes in sarcoma specimens. Overall survival (OS) by low and high CDK1, CCNB1, CDC20, CCNB2, AURKB, MAD2L1, CENPE, KIF2C, and PCNA expression

We used KM plotter, which contains 259 sarcoma samples, including Ewing’s sarcoma, synovial sarcoma, RMS and other sarcomas, to analyse the prognostic impact of hub genes identified. Except for HIST2H2BE, the remaining genes were highly expressed and associated with poor prognosis (*P* < 0.05) (Fig. 5). The experiments further revealed that, except for HIST2H2BE, the remaining genes were highly expressed in RMS. These studies indirectly imply that overexpression of these genes may have a negative impact on the prognosis of RMS patients.

### RT-PCR validation of the mRNA expression of hub genes

**Fig. 6:**
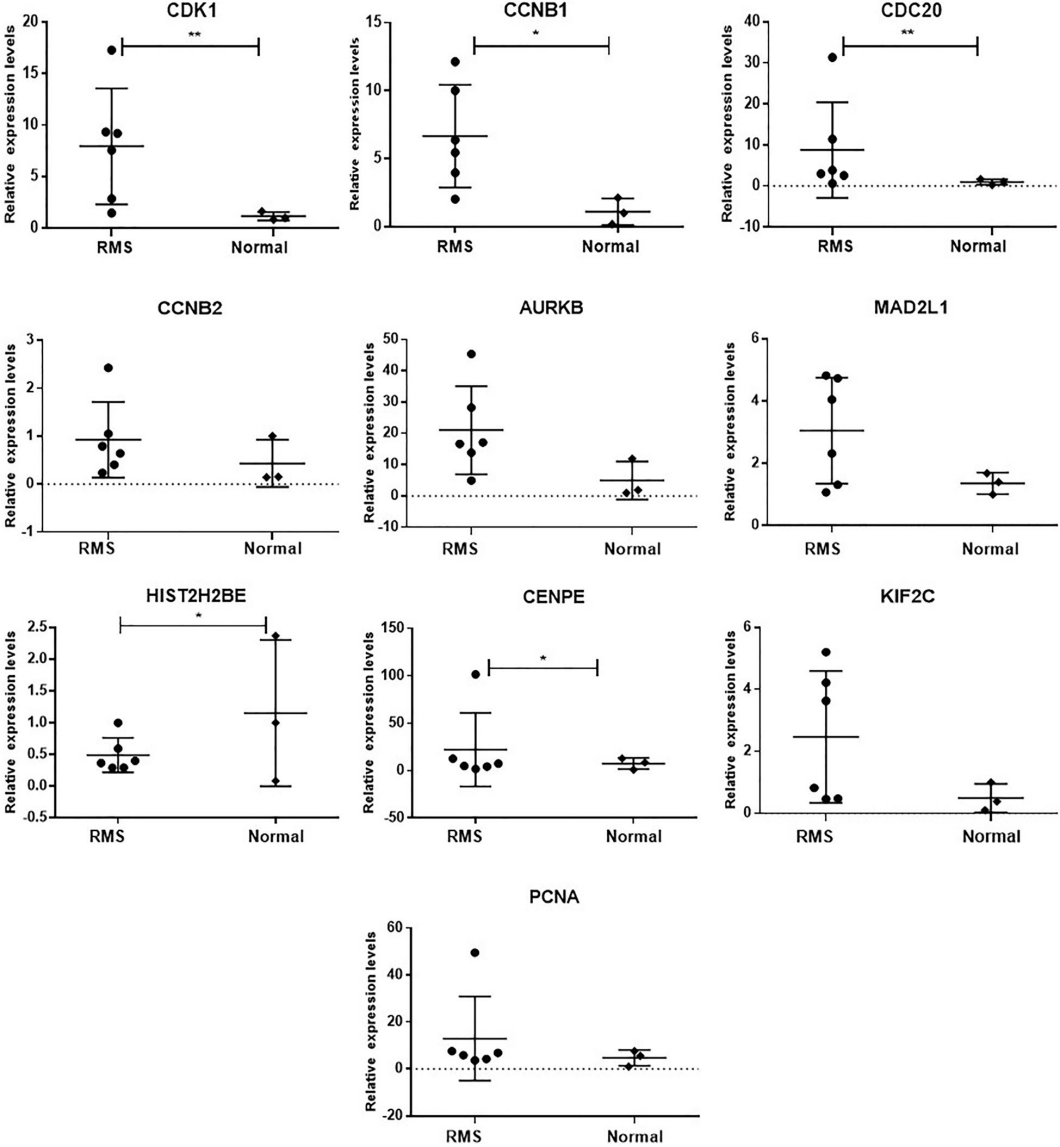
Validation of 10 hub genes by RT-PCR. mRNA expression levels of 10 hub genes were detected in RMS patient tissue and normal striated muscle tissue. Each point represents an individual subject. *p < 0.05; **p < 0.01

To test the results of the previous analysis, we further used RT-PCR to detect the mRNA expression of the hub gene. The 10 hub genes contain nine up-regulated genes (CDK1, CCNB1, CDC20, CCNB2, AURKB, MAD2L1, CENPE, KIF2C, and PCNA) and one down-regulated gene (HIST2H2BE). The RT-PCR data showed that although the trend of expression patterns of these 10 hub genes were consistent with the sequencing results, among these up-regulated genes, only CDK1, CCNB1, CDC20 and CENPE were significantly up-regulated in RMS. In addition, the expression of HIST2H2BE was reduced in RMS patients (Fig. 6).

### CDK1 was highly expressed in RMS patients but has no significant association with clinical features

**Fig. 7:**
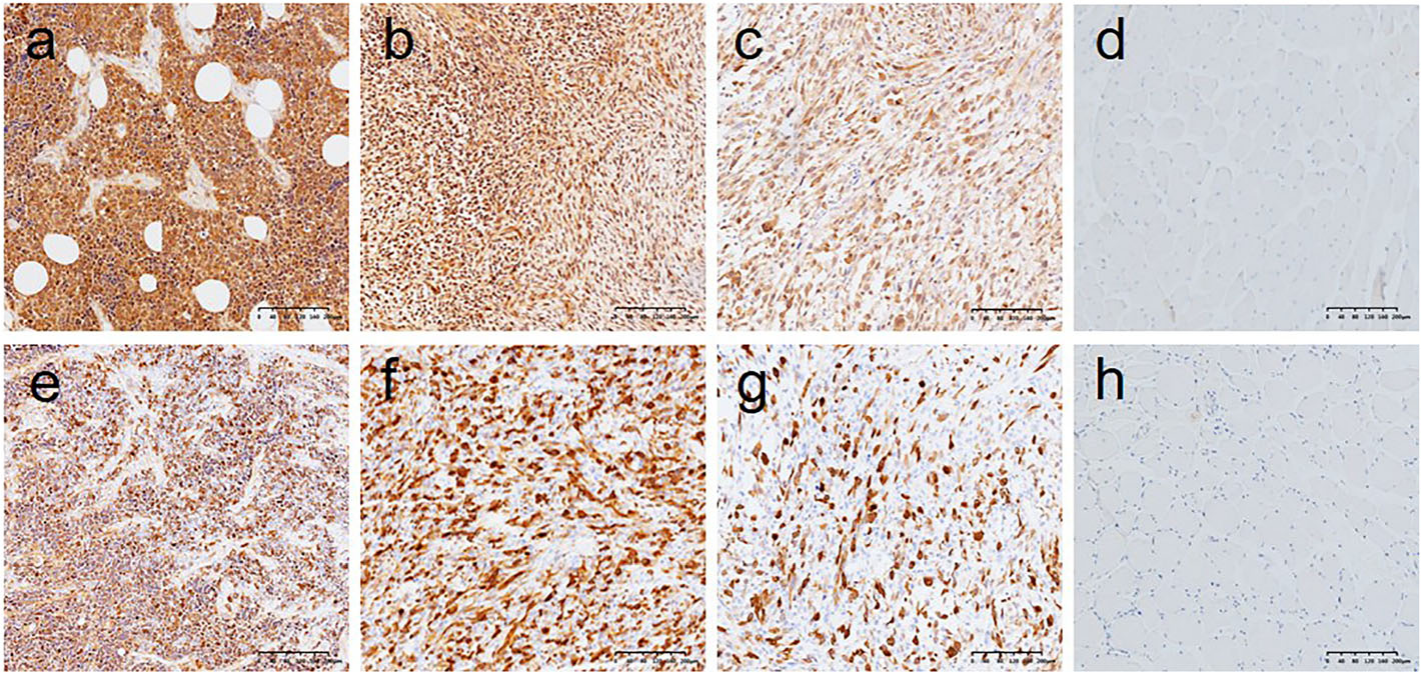
Immunohistochemical staining of CDK1and CCNB1 expression in rhabdomyosarcoma and normal muscle tissues. Immunohistochemical staining for CDK1 demonstrated strong nuclear expression in RMS. **a**-**c** RMS (× 100); **d** Normal (× 100). Immunohistochemical staining for CCNB1 demonstrated nuclear or cytoplasm expression in RMS. **e**-**g** RMS (× 100); h Normal (× 100)

To understand the potential functions of hub genes in RMS, we selected hub genes based on their degree of connectivity. Amongst the hub genes showing remarkable changes in the RMS samples, we selected two up-regulated genes (CDK1 and CCNB1) for further protein expression testing. The main staining part of CDK1 mainly showed staining in the nucleus and revealed an expression rate of 87.5% (28/32) in 32 RMS cases (Fig. 7a, b and c are ARMS, ERMS and PRMS sample tissues, respectively). The expression rate of CDK1 in normal striated muscle samples was 11.1% (1/9), which is relatively low (Fig. 7d). The expression rate of CDK1 was notably superior in RMS than in the normal controls (*P* = 0.000). No statistically significant differences between CDK1 expression and clinical date were observed (Table 3).

**Table 3:** Association between CDK1and CCNB1 protein expression and clinical features

		CDK1		*p*-value	CCNB1		*p*-value
Gender	n	≤2	>3	1.000	≤3	>3	0.196
Male	18	2(11.11)	16(88.89)		5(27.78)	13(72.22)	
Female	14	2(14.29)	12(85.71)		1(7.14)	13(92.86)	
Age (years)	n			1.000			^a^0.018
≤5	16	2(87.50)	14(12.50)		6(37.50)	10(62.50)	
> 5	16	2(87.50)	14(12.50)		0(0.00)	16(100.00)	
Tumor diameter	n			0.601			0.066
≤5 cm	20	2(10.00)	18(90.00)		6(30.00)	14(70.00)	
> 5 cm	11	2(18.18)	9(81.82)		0(0.00)	11(100.00)	
Ethnicity	n			1.000			1.000
Han	11	1(9.09)	10(90.91)		2(18.18)	9(81.82)	
^b^Other minorities	20	3(15.00)	17(85.00)		4(20.00)	16(80.00)	
Location	n			0.488			^a^0.036
Head and neck	13	1(7.69)	12(92.31)		3(23.08)	10(76.92)	
Torso and limbs	8	1(12.50)	7(87.50)		0(0.00)	8(100.00)	
Urinary	5	1(20.00)	4(80.00)		3(60.00)	2(40.00)	
Abdominal or retroperitoneal	6	1(16.67)	5(83.34)		0(0.00)	6(100.00)	

*P* < 0.05 indicates a significant association among the variables

^a^Significant difference

^b^including Uygur (*n* = 18), Kazak (*n* = 1) and Hui (*n* = 1)

### CCNB1 was highly expressed in RMS patients and was related with age and location

CCNB1 is mainly stained in the nucleus or cytoplasm of RMS, however only in the cytoplasmic staining of normal striated muscle. CCNB1 showed expression rates of 81.25% (26/32) in RMS cases (Fig. 7e, f and g are ARMS, ERMS and PRMS tissue samples, respectively) and 0% (0/9) in normal striated muscle samples (Fig. 7h). Consistent with previous analyses, CCNB1 was highly expressed in RMS patients compared to normal controls (P = 0.000). CCNB1 expression was related with years (*P* = 0.018) and tumour location (*P* = 0.036) in RMS patients (Table 3).

## Discussion

RMS is a malignant tumour that becomes invasive when cells fail to differentiate fully [24]. Although RMS is rare compared to other cancer types, it may develop in any part of the body, such as the head, neck, genitourinary tract or limbs [25]. Considering the poor prognosis of RMS, understanding the specific biomarkers of the disease is essential for early diagnosis and therapy to reduce morbidity and death rates.

We analysed the DGEs of RMS and striated muscle samples. Amongst 10 hub genes identified, that of CDK1 exhibited the highest degree of connectivity, followed by CCNB1. CDK1, a protein encoding the Ser / Thr protein kinase, is a cell cycle-dependent kinase. CCNB1-encoded proteins regulate cell mitosis. The maturation promoting factor formed by the binding of this gene product to CDK1 is required for G1 / S and G2 / M transformation in the cell cycle. We found that increased expression of CDK1 and CCNB1 in RMS is related with negative prognosis in sarcoma sufferers. Data analysis of IHC also showed that high levels of CCNB1 occurs in sufferers older than 5 years. Tumours occur mostly in the head and neck and are rare in the trunk or limbs. CDK1 and CCNB1 have been reported to promote the development of glioblastoma malignancies [9] and are considered potential prognostic biomarkers or therapeutic targets for patients with hepatocellular carcinoma [10, 11]. In RMS, E-3-(4′-hydroxy-3′-adamantylbiphenyl-4-yl) acrylic acid reduces CDK1 protein level and inhibited RMS cell activity [26]. The CCNB1 signature presents a promising diagnostic tool for identifying sufferers with non-invasive bladder tumour with a higher risk of recurrence and predicting responses to IVT [12]. RMS in heterozygous p53 mice exhibits higher CCNB1 expression than RMS in heterozygous Patched1 (Ptch1) mice [27]. Based on these previous reports, CDK1 and CCNB1 promote the occurrence or are potential biomarkers of various diseases and may play the same roles in RMS.

Previous studies report that the cell cycle of RMS is shortened and that incompletely differentiated muscles cause tumour development [28, 29]. The present study found that high-expression genes were heavily enriched in cell formation and cell cycle transition whereas low-expression genes were mainly enriched in formation of muscles, development of muscle organs and regulation of muscle protein binding. This phenomenon demonstrates that the DGEs determined in this study, especially the cell cycle-related factors CDK1 and CCNB1, may play a considerable role in tumourigenesis.

Amongst the eight remaining hub genes, CDC20, CCNB2, AURKB, MAD2L1, CENPE, KIF2C, and PCNA were highly expressed and related with negative prognosis of sarcoma. CDC20 regulates cyclins and interacts with various proteins. Guo et al. found that CDC20 is regulated by lncRNA SPRY4-IT1 to induce cell growing and invasion in pancreatic tumour [30]. Wang et al. reported that CDC20 exhibits cancer-promoting effects. Thus, targeting CDC20 in cancer may be a potential treatment strategy [31]. Wang et al. reported that CDC20 serves as a meaningful prognostic biomarker in most cases of human solid tumours and that its high expression reduces patient survival [32]. HMGA-induced CCNB2 (a molecule of the cyclin clan) plays an important role in the development of mouse or human pituitary tumours [33]. Up-regulation of CCNB2 has a negative effect on the outcome of sufferers with non-small cell lung carcinoma [34]. AURKB, an aurora kinase encoding a serine/threonine kinase, binds to microtubules and is involved in chromosomal regulation during mitosis and meiosis. Al-Khafaji et al. reported that paclitaxel promotes responses via AURKB in non-small cell lung tumour [35]. MAD2L1, one of the components of the mitotic spindle assembly checkpoint, appropriately aligns all chromosomes on the medium plate. Zhong et al. found that MAD1L1 and MAD2L1 increases the likelihood of colorectal cancer amongst smokers [36]. Zhu et al. reported that CDK1 and MAD2L1 are markers of poor prognosis in lung adenocarcinoma [37]. HIST2H2BE (H2B), an element of the histone family, are responsible for the chromosomal fibre nucleosome structure in eukaryotes. Spolverini et al. reported that let-7b / c microRNA inhibits cell migration by regulating HIST2H2BE deubiquitination to ubiquitination [38]. CENPE (centroid-associated protein E), a cell cycle-driven motor protein, acts on the early stages of chromosome alignment. Kung et al. reported that CENPE may play a treatment mark role in sufferers with triple-negative/basal a-type breast tumour [39]. KIF2C encodes a protein with a kinesin function through the movement of microtubule-dependent molecules. Duan et al. reported that KIF-2C expression affects the bad ending of males with surgical oesophageal squamous cell carcinoma [40].

The ubiquitination of PCNA involves DNA repair. Wang et al. reported that miR-363-3p controls the growth of lung adenocarcinoma by reducing the expression of PCNA [41]. Such studies reveal that these genes are associated with the prognosis of a variety of tumours and may become biomarkers of RMS in the future.

The occurrence of tumours is affected by many aspects, and the roles of signalling pathways in tumours have become a popular topic of scientific research. In this study, P53, ECM-receptor interaction, cancer, viral cancelation, and PI3K-Akt signaling pathways were identified. Inactivation of the P53 pathway could induce the development of RMS [42]. Hawkins et al. [43] found that ECM mainly acts on tumour metastasis. As the PI3K-Akt signalling is active in RMS, targeting this pathway could inhibit the activation of hypoxia-inducible factor-1α, thereby inducing the formation of apoptosis-sensitive cells [44]. Studies on these pathways to provide feasible ideas for the pathogenesis of the disease present a future research direction for RMS.

## Conclusions

The study applied a comprehensive analysis of DEGs in RMS and normal striated muscles and discovered a total of 1932 DEGs related with RMS development. Some genes were involved in changes in the cell cycle, P53 and PI3K-Akt signalling pathways. We also found that the cell cycle regulatory molecules CDK1 and CCNB1 are the most highly connected genes amongst the hub genes and highly expressed in RMS. The increased expression of these genes may contribute to the growth of RMS tumour cells. Therefore, CDK1 and CCNB1 may be biomarkers for the potential diagnosis and therapy in RMS disease. However, our results require further validation using larger cohorts.

## Supplementary information

**Additional file 1: Table S1.** Primer information for RT-PCR in this study.

**Additional file 2: Table S2.** DEGs between RMS and normal striated muscle samples.

**Additional file 3: Figure S1.** PPI network of DEGs. Each node expresses a protein, and each line represents the relationship of proteins to each other. Nodes of different colors have different score values.

## Data Availability

The authors declare that the data supporting the results of this study are provided in this paper. The original data for this study are from the datasets. GSE16382 [https://www.ncbi.nlm.nih.gov/geo/query/acc.cgi?acc=GSE16382], GSE66533 [https://www.ncbi.nlm.nih.gov/geo/query/acc.cgi?acc=GSE66533], GSE39454 [https://www.ncbi.nlm.nih.gov/geo/query/acc.cgi?acc=GSE39454], GSE17674 [https://www.ncbi.nlm.nih.gov/geo/query/acc.cgi?acc=GSE17674] and GSE38417 [https://www.ncbi.nlm.nih.gov/geo/query/acc.cgi?acc=GSE38417]. The datasets generated during the current study are available in the GEO Database Series GSE141690 repository, [https://www.ncbi.nlm.nih.gov/geo/query/acc.cgi?acc=GSE141690].

## Abbreviations

ARMS: Alveolar RMS
BP: Biological process
CC: Cell composition
DEGs: Differentially expressed genes
ERMS: Embryonal RMS
GEO: Gene Expression Omnibus
GO: Gene ontology
IHC: Immunohistochemistry
KEGG: Kyoto Encyclopedia of Genes and Genomes
KM plotter: Kaplan–Meier plotter
MCODE: Molecular Complex Detection
MF: Molecular function
PPI: Protein–protein interaction
PRMS: Pleomorphic RMS
RMS: Rhabdomyosarcoma
SRMS: Spindle cell/sclerosing RMS
STRING: Search tool for the retrieval of interacting genes
