# Supplementary material for: CDK1 and CCNB1 as potential diagnostic markers of rhabdomyosarcoma: validation following bioinformatics analysis
Source: BMC Med Genomics. 2019 Dec 23;12:198. doi: 10.1186/s12920-019-0645-x (PMC6929508; doi:10.1186/s12920-019-0645-x)
Supplement: Supplementary file 1 — Additional file 1: Table S1. Primer information for RT-PCR in this study. [file 12920_2019_645_MOESM1_ESM.docx]

**Table S1.** Primer information for RT-PCR in this study.

| **Gene name** |  | **Primer sequence** |
| --- | --- | --- |
| CDK1 | Forward | 5'-GCGGAATAATAAGCCGGGAT -3' |
|  | Reverse | 5'-CAACTCCATAGGTACCTTCTCCA-3' |
| CCNB1 | Forward | 5'-GTTATGCAGCACCTG-3' |
|  | Reverse | 5'-CTTGGCTAAATCTTGAACT-3' |
| CDC20 | Forward | 5′- GACCACTCCTAGCAAACCTGG -3′ |
|  | Reverse | 5′-GGG CGT CTG GCT GTT TTC A-3′ |
| CCNB2 | Forward | 5′-GCGTTGGCATTATGGATCG-3′ |
|  | Reverse | 5′-TCTTCCGGGAAACTGGCTG-3′ |
| AURKB | Forward | 5’- GTCACCCCATCTGCACTTGT-3' |
|  | Reverse | 5’-ACGCCCAATCTCAAAGTCAT-3' |
| MAD2L1 | Forward | 5' - AGCTCCTTTTGACCTTCATTTC-3' |
|  | Reverse | 5'- TCCATTGCTTCATAGGTTCAAG-3' |
| KIF2C | Forward | 5'-GATGGAAGCCTGCTCTAACG-3' |
|  | Reverse | 5'-GAGCAGATTCCGCTTTGTTC-3' |
| PCNA | Forward | 5'-ACCGCTGCGACCGCAATTTG-3' |
|  | Reverse | 5'-ACGTGCAAATTCACCAGAAGGCATC-3' |
| CENPE | Forward | 5'-AAGACCGAGCTTTCTTACAAGA-3' |
|  | Reverse | 5'-CTACAGTTTGCAGCGTAGAATC-3' |
| HIST2H2BE | Forward | 5'-GAGCTACTCCATCTACGTGTAC-3' |
|  | Reverse | 5'-GTTGACGAAGGAGTTCATGATG-3' |
| GAPDH | Forward | 5'- GACAGTCAGCCGCATCTTCT-3' |
|  | Reverse | 5'- TTAAAAGCAGCCCTGGTGAC-3' |
