# Supplementary material for: CDK1 and CCNB1 as potential diagnostic markers of rhabdomyosarcoma: validation following bioinformatics analysis
Source: BMC Med Genomics. 2019 Dec 23;12:198. doi: 10.1186/s12920-019-0645-x (PMC6929508; doi:10.1186/s12920-019-0645-x)
Supplement: Supplementary file 2 — Additional file 2: Table S2. DEGs between RMS and normal striated muscle samples. [file 12920_2019_645_MOESM2_ESM.docx]

**Table S2.** Differentially expressed genes between RMS and normal striated muscle samples.

| Gene Name | logFC | P.Value | FDR |
| --- | --- | --- | --- |
| C3orf43 | -4.82982 | 6.13E-71 | 1.26E-66 |
| PEBP4 | -6.70668 | 6.61E-69 | 6.76E-65 |
| KCNA7 | -4.50223 | 3.70E-68 | 2.52E-64 |
| RPL3 | 4.664811 | 1.96E-67 | 1.00E-63 |
| ZNF486 | 2.733586 | 2.23E-65 | 9.14E-62 |
| LOC101929592 | -4.61001 | 3.05E-64 | 1.04E-60 |
| PCDHGB5 | -2.80836 | 1.01E-61 | 2.96E-58 |
| IDI2 | -5.06526 | 2.82E-60 | 7.21E-57 |
| ASB10 | -3.03885 | 8.34E-59 | 1.90E-55 |
| DLG3-AS1 | -2.40586 | 1.33E-58 | 2.72E-55 |
| PPP1R3A | -5.92013 | 3.93E-58 | 7.32E-55 |
| ASB11 | -4.71816 | 9.86E-58 | 1.68E-54 |
| UCP3 | -3.55174 | 2.28E-57 | 3.59E-54 |
| HMGN1 | 4.800907 | 2.57E-57 | 3.75E-54 |
| CDNF | -3.9138 | 2.33E-56 | 3.18E-53 |
| C15orf27 | -2.87115 | 3.84E-56 | 4.91E-53 |
| PABPC3 | 4.263288 | 4.15E-56 | 4.99E-53 |
| ACR | -2.397 | 5.41E-56 | 6.15E-53 |
| NACAP1 | 2.125383 | 5.71E-56 | 6.15E-53 |
| RPL3L | -5.77698 | 1.66E-55 | 1.70E-52 |
| PPP1R27 | -3.46313 | 2.23E-55 | 2.18E-52 |
| SLC2A4 | -3.05983 | 2.56E-55 | 2.38E-52 |
| PABPC1 | 2.057993 | 5.07E-54 | 4.51E-51 |
| LOC646736 | -3.2304 | 1.60E-53 | 1.31E-50 |
| CNKSR1 | -3.12718 | 2.27E-53 | 1.79E-50 |
| YBX2 | -2.63822 | 2.57E-53 | 1.95E-50 |
| LINC01304 | -2.07609 | 3.67E-53 | 2.68E-50 |
| FBXO40 | -5.21429 | 2.30E-52 | 1.63E-49 |
| FEM1A | -2.68373 | 7.25E-52 | 4.95E-49 |
| PPP1R1A | -5.91051 | 1.49E-51 | 9.86E-49 |
| NRAP | -6.70994 | 5.53E-51 | 3.53E-48 |
| FBP2 | -6.70658 | 8.98E-51 | 5.57E-48 |
| RPL19 | 2.08797 | 1.54E-50 | 8.99E-48 |
| RPS19 | 2.317942 | 3.85E-50 | 2.12E-47 |
| C10orf71-AS1 | -3.92944 | 3.94E-50 | 2.12E-47 |
| PPM1J | -3.20325 | 7.05E-50 | 3.70E-47 |
| LOC100507537 | -7.47914 | 7.48E-50 | 3.83E-47 |
| MBNL1-AS1 | -5.10013 | 8.04E-50 | 4.01E-47 |
| TCP10 | 2.441898 | 1.72E-49 | 8.18E-47 |
| CLCN1 | -2.08759 | 3.05E-49 | 1.42E-46 |
| C20orf166 | -3.45401 | 5.49E-49 | 2.44E-46 |
| UPK3A | -2.88377 | 5.77E-49 | 2.51E-46 |
| TCAP | -6.39095 | 1.05E-48 | 4.48E-46 |
| SPN | -2.46046 | 1.11E-48 | 4.65E-46 |
| LDHD | -3.38652 | 1.43E-48 | 5.84E-46 |
| FHL3 | -4.40102 | 2.08E-48 | 8.33E-46 |
| S100A1 | -4.49454 | 2.52E-47 | 9.72E-45 |
| KLHL34 | -3.30824 | 4.92E-47 | 1.86E-44 |
| ADCK3 | -3.92372 | 6.74E-47 | 2.51E-44 |
| LRRC2 | -6.54962 | 1.25E-46 | 4.50E-44 |
| FSD2 | -5.32322 | 1.46E-46 | 5.14E-44 |
| IL17C | -2.19048 | 5.48E-46 | 1.90E-43 |
| NOTCH2NL | 3.843382 | 6.83E-46 | 2.33E-43 |
| CPEB3 | -2.02073 | 1.22E-45 | 4.04E-43 |
| CAMK2A | -2.35285 | 1.71E-45 | 5.54E-43 |
| LOC101930114 | -2.63563 | 2.26E-45 | 7.11E-43 |
| PCDHB12 | 2.185292 | 2.31E-45 | 7.17E-43 |
| MSS51 | -4.56302 | 3.25E-45 | 9.91E-43 |
| NPM1 | 3.12512 | 8.25E-45 | 2.45E-42 |
| NGFRAP1 | 3.765555 | 1.24E-44 | 3.58E-42 |
| RPS3 | 2.235051 | 1.66E-44 | 4.73E-42 |
| MYOC | -2.67066 | 2.63E-44 | 7.36E-42 |
| NEK10 | -2.31962 | 3.40E-44 | 9.41E-42 |
| WBP5 | 3.573472 | 5.18E-44 | 1.38E-41 |
| RPS7 | 2.128064 | 6.02E-44 | 1.58E-41 |
| GOLM1 | 2.140414 | 1.13E-43 | 2.83E-41 |
| UBE2D1 | -2.26725 | 1.16E-43 | 2.86E-41 |
| KHDRBS1 | 2.289538 | 1.23E-43 | 2.99E-41 |
| LOC642852 | 2.358905 | 5.00E-43 | 1.18E-40 |
| ZNF559 | 4.316563 | 8.92E-43 | 1.98E-40 |
| LOC102723721 | -2.88653 | 1.10E-42 | 2.39E-40 |
| ZNF765 | 2.209326 | 3.19E-42 | 6.87E-40 |
| RAP1GDS1 | 2.783148 | 3.35E-42 | 7.13E-40 |
| MYH7 | -7.9604 | 3.57E-42 | 7.54E-40 |
| AGMAT | -2.37129 | 4.11E-42 | 8.58E-40 |
| KIAA1161 | -2.05757 | 5.24E-42 | 1.05E-39 |
| SLC25A6 | 3.495154 | 5.49E-42 | 1.09E-39 |
| SCN1B | -3.75188 | 7.32E-42 | 1.44E-39 |
| FAT1 | 4.339374 | 8.54E-42 | 1.66E-39 |
| YWHAQ | 2.950092 | 9.62E-42 | 1.86E-39 |
| IKBIP | 3.261668 | 1.14E-41 | 2.18E-39 |
| KIF3B | 2.356956 | 1.49E-41 | 2.81E-39 |
| MYH4 | -4.19862 | 1.59E-41 | 2.96E-39 |
| TNNI3 | -2.79226 | 1.74E-41 | 3.20E-39 |
| MBOAT2 | 2.962399 | 2.37E-41 | 4.33E-39 |
| TUBA1A | 3.849277 | 2.42E-41 | 4.38E-39 |
| P2RY2 | -3.20913 | 2.50E-41 | 4.50E-39 |
| ZC3HAV1L | 2.735294 | 2.58E-41 | 4.59E-39 |
| PRR11 | 4.048214 | 2.81E-41 | 4.95E-39 |
| PHKG1 | -2.18954 | 3.34E-41 | 5.85E-39 |
| TUBA1B | 2.399316 | 3.45E-41 | 5.98E-39 |
| MYOZ3 | -2.24225 | 4.19E-41 | 7.20E-39 |
| CLIC5 | -3.43254 | 5.09E-41 | 8.69E-39 |
| CKMT2-AS1 | 3.485843 | 1.03E-40 | 1.70E-38 |
| LSMEM1 | -3.90355 | 1.15E-40 | 1.89E-38 |
| ZSWIM6 | 3.955039 | 1.34E-40 | 2.16E-38 |
| SND1 | 2.379557 | 1.94E-40 | 3.10E-38 |
| SPIN1 | 3.450327 | 2.26E-40 | 3.54E-38 |
| ABRA | -6.02028 | 2.43E-40 | 3.75E-38 |
| COX6A2 | -6.01481 | 4.40E-40 | 6.58E-38 |
| MYL3 | -6.56783 | 4.63E-40 | 6.87E-38 |
| TXNDC12 | 2.333431 | 5.19E-40 | 7.64E-38 |
| HSPB6 | -5.53583 | 5.65E-40 | 8.25E-38 |
| PWP1 | 2.222914 | 5.95E-40 | 8.58E-38 |
| PABPC1L | 3.064753 | 9.84E-40 | 1.37E-37 |
| ALDH18A1 | 3.173266 | 1.02E-39 | 1.41E-37 |
| ADSSL1 | -3.49861 | 1.16E-39 | 1.59E-37 |
| NEK4 | 2.076603 | 1.38E-39 | 1.86E-37 |
| LRRC20 | -2.96908 | 1.44E-39 | 1.92E-37 |
| DENND2C | -3.02703 | 1.54E-39 | 2.04E-37 |
| IDI2-AS1 | -2.75437 | 1.66E-39 | 2.19E-37 |
| TMED9 | 2.392125 | 1.73E-39 | 2.27E-37 |
| C1orf94 | -2.31622 | 2.14E-39 | 2.79E-37 |
| FN3KRP | 2.260135 | 2.17E-39 | 2.81E-37 |
| PERM1 | -2.52837 | 2.29E-39 | 2.95E-37 |
| GAS5 | 2.676357 | 2.41E-39 | 3.09E-37 |
| SOX9-AS1 | -2.202 | 3.65E-39 | 4.58E-37 |
| RACGAP1 | 5.540679 | 3.74E-39 | 4.66E-37 |
| NDUFB10 | -2.49397 | 3.77E-39 | 4.67E-37 |
| POGLUT1 | 2.76109 | 4.33E-39 | 5.31E-37 |
| TYRP1 | -3.84143 | 4.33E-39 | 5.31E-37 |
| LOC101928954 | 2.585447 | 4.61E-39 | 5.61E-37 |
| STK36 | 2.461287 | 6.08E-39 | 7.23E-37 |
| SNTA1 | -3.20384 | 6.81E-39 | 8.06E-37 |
| CENPW | 4.459007 | 6.87E-39 | 8.08E-37 |
| PTMA | 2.850514 | 1.08E-38 | 1.25E-36 |
| EEF1A2 | -6.03393 | 1.09E-38 | 1.25E-36 |
| LINC00342 | 4.194296 | 1.15E-38 | 1.32E-36 |
| HHATL | -5.58638 | 1.29E-38 | 1.44E-36 |
| MAP2K6 | -3.09903 | 1.43E-38 | 1.59E-36 |
| ATG4C | 3.172842 | 1.49E-38 | 1.64E-36 |
| RBBP8 | 4.229982 | 1.53E-38 | 1.68E-36 |
| FKBP11 | 2.99083 | 1.82E-38 | 1.97E-36 |
| SPIN4 | 5.289954 | 2.42E-38 | 2.60E-36 |
| GSDMC | -2.68186 | 2.89E-38 | 3.07E-36 |
| PPIP5K2 | 3.981417 | 2.97E-38 | 3.11E-36 |
| RIPK2 | 2.431484 | 2.98E-38 | 3.11E-36 |
| TMEM52 | -5.55475 | 3.38E-38 | 3.45E-36 |
| ZNF468 | 4.164345 | 4.19E-38 | 4.21E-36 |
| CASP6 | 2.387627 | 4.97E-38 | 4.95E-36 |
| TUG1 | 2.242418 | 4.98E-38 | 4.95E-36 |
| PXDN | 4.740516 | 5.20E-38 | 5.14E-36 |
| PTTG1IP | 3.000087 | 5.48E-38 | 5.36E-36 |
| FUBP3 | 2.399634 | 5.86E-38 | 5.71E-36 |
| LINC00116 | -3.60462 | 5.99E-38 | 5.81E-36 |
| HSPB11 | 2.670122 | 6.12E-38 | 5.91E-36 |
| TBCA | 2.72475 | 6.39E-38 | 6.14E-36 |
| DSN1 | 3.931976 | 6.45E-38 | 6.17E-36 |
| CUTC | -3.12101 | 6.92E-38 | 6.59E-36 |
| MRPL33 | -2.24498 | 6.99E-38 | 6.62E-36 |
| LPCAT1 | 3.2368 | 7.62E-38 | 7.12E-36 |
| WDR53 | 2.059996 | 8.33E-38 | 7.75E-36 |
| MYLK2 | -2.69795 | 8.96E-38 | 8.26E-36 |
| CASQ1 | -8.06056 | 9.55E-38 | 8.76E-36 |
| KIAA0101 | 6.103882 | 1.04E-37 | 9.45E-36 |
| ZWINT | 5.303183 | 1.14E-37 | 1.03E-35 |
| LOC389199 | -2.03338 | 1.19E-37 | 1.07E-35 |
| SLC25A4 | -3.4979 | 1.37E-37 | 1.23E-35 |
| RCN1 | 3.311837 | 1.44E-37 | 1.28E-35 |
| PNMAL1 | 5.469285 | 1.46E-37 | 1.29E-35 |
| TUBA1C | 2.618078 | 1.62E-37 | 1.42E-35 |
| FAM122B | 2.964458 | 2.29E-37 | 2.00E-35 |
| MYPN | -4.12548 | 2.53E-37 | 2.20E-35 |
| CHSY1 | 4.499346 | 2.60E-37 | 2.25E-35 |
| SEC23B | 2.97123 | 5.18E-37 | 4.39E-35 |
| DROSHA | 2.651826 | 7.77E-37 | 6.51E-35 |
| CA4 | -2.04537 | 7.87E-37 | 6.56E-35 |
| METTL3 | 2.416751 | 8.37E-37 | 6.90E-35 |
| HIF1A | 3.375823 | 8.37E-37 | 6.90E-35 |
| ARL6IP1 | 3.426174 | 9.29E-37 | 7.54E-35 |
| CLIP2 | 3.301125 | 9.56E-37 | 7.73E-35 |
| PGAM2 | -6.36819 | 1.05E-36 | 8.36E-35 |
| PAPD7 | 3.031718 | 1.09E-36 | 8.68E-35 |
| SNAI2 | 4.460178 | 1.16E-36 | 9.22E-35 |
| TYMS | 3.849108 | 1.23E-36 | 9.67E-35 |
| TMEM87B | 2.923451 | 1.24E-36 | 9.75E-35 |
| KRBOX4 | 2.600974 | 1.55E-36 | 1.21E-34 |
| ANO5 | -5.88192 | 1.61E-36 | 1.25E-34 |
| GLA | 2.249525 | 1.69E-36 | 1.31E-34 |
| RCC2 | 2.548981 | 2.16E-36 | 1.65E-34 |
| TAF1D | 4.388108 | 2.17E-36 | 1.66E-34 |
| FAM166B | -3.26411 | 2.21E-36 | 1.68E-34 |
| TMSB10 | 2.969814 | 2.37E-36 | 1.79E-34 |
| RAI14 | 4.310902 | 2.37E-36 | 1.79E-34 |
| PNN | 2.428541 | 2.52E-36 | 1.88E-34 |
| CKS2 | 5.877147 | 2.53E-36 | 1.88E-34 |
| KIAA0895L | 4.201867 | 2.53E-36 | 1.88E-34 |
| CMTR1 | 2.067747 | 2.90E-36 | 2.14E-34 |
| RNF144B | -2.58458 | 2.96E-36 | 2.17E-34 |
| UGDH | 3.133452 | 3.53E-36 | 2.56E-34 |
| ARMCX2 | 3.763714 | 3.57E-36 | 2.58E-34 |
| GOT2 | -2.23427 | 3.63E-36 | 2.62E-34 |
| REST | 2.022772 | 4.16E-36 | 2.99E-34 |
| SLC38A6 | 4.11311 | 4.36E-36 | 3.11E-34 |
| MYOZ1 | -7.06085 | 4.38E-36 | 3.11E-34 |
| MRPS21 | 2.706482 | 5.00E-36 | 3.53E-34 |
| ASAP2 | 3.790532 | 5.44E-36 | 3.81E-34 |
| UHRF2 | 2.416984 | 5.68E-36 | 3.97E-34 |
| EPM2A | -2.30605 | 5.71E-36 | 3.97E-34 |
| RPL36 | 2.58786 | 6.44E-36 | 4.45E-34 |
| ZWILCH | 3.564702 | 6.49E-36 | 4.46E-34 |
| ZFAS1 | 3.493513 | 6.60E-36 | 4.51E-34 |
| ATP13A1 | 2.472737 | 6.83E-36 | 4.64E-34 |
| GALNT7 | 4.105592 | 7.16E-36 | 4.84E-34 |
| PDCD6 | 2.35805 | 7.20E-36 | 4.85E-34 |
| GOT1 | -3.64996 | 7.24E-36 | 4.86E-34 |
| TAF2 | 2.525243 | 8.07E-36 | 5.38E-34 |
| C5orf34 | 4.159936 | 9.23E-36 | 6.11E-34 |
| COQ10A | -3.25818 | 1.05E-35 | 6.88E-34 |
| TTC31 | 2.559099 | 1.26E-35 | 8.24E-34 |
| SHQ1 | 2.199372 | 1.33E-35 | 8.64E-34 |
| MITD1 | 2.881927 | 1.33E-35 | 8.64E-34 |
| SLC36A4 | 2.463134 | 1.45E-35 | 9.36E-34 |
| SHCBP1 | 5.166254 | 1.48E-35 | 9.55E-34 |
| EFHC1 | 2.360588 | 1.68E-35 | 1.07E-33 |
| AKR1A1 | 2.065943 | 1.76E-35 | 1.11E-33 |
| IFT20 | 2.614472 | 1.93E-35 | 1.22E-33 |
| FAM193A | 2.182463 | 2.01E-35 | 1.26E-33 |
| PGM3 | 2.478816 | 2.04E-35 | 1.28E-33 |
| SLC33A1 | 2.063804 | 2.32E-35 | 1.44E-33 |
| ZNF439 | 3.383762 | 2.33E-35 | 1.45E-33 |
| OFD1 | 2.510139 | 2.38E-35 | 1.48E-33 |
| FMR1 | 3.51594 | 2.71E-35 | 1.67E-33 |
| NACA | 2.655673 | 3.03E-35 | 1.86E-33 |
| CASP3 | 3.33099 | 3.71E-35 | 2.25E-33 |
| IPO13 | -2.00591 | 4.61E-35 | 2.76E-33 |
| RAD51AP1 | 5.948973 | 4.71E-35 | 2.80E-33 |
| ARL13B | 3.376878 | 4.90E-35 | 2.91E-33 |
| C18orf54 | 2.066304 | 5.34E-35 | 3.16E-33 |
| ZNF518A | 2.743031 | 5.42E-35 | 3.19E-33 |
| ANP32B | 2.341401 | 5.55E-35 | 3.24E-33 |
| TRIM7 | -3.33252 | 5.97E-35 | 3.48E-33 |
| DNAJB11 | 2.560231 | 5.99E-35 | 3.48E-33 |
| KIAA0753 | 2.382667 | 6.52E-35 | 3.78E-33 |
| DAPK1 | 3.179661 | 7.14E-35 | 4.11E-33 |
| FLVCR1 | 4.371734 | 7.20E-35 | 4.14E-33 |
| NDUFC1 | -2.04148 | 7.64E-35 | 4.36E-33 |
| MYADML2 | -2.42303 | 8.28E-35 | 4.72E-33 |
| ZNF320 | 3.44179 | 8.33E-35 | 4.73E-33 |
| C2CD5 | 3.27571 | 8.70E-35 | 4.92E-33 |
| WDR75 | 2.619349 | 8.86E-35 | 4.99E-33 |
| HIBADH | -2.32182 | 9.33E-35 | 5.25E-33 |
| KIF2A | 3.159576 | 9.78E-35 | 5.48E-33 |
| POLK | 2.194874 | 1.03E-34 | 5.77E-33 |
| EGF | -4.64789 | 1.06E-34 | 5.90E-33 |
| RAD23A | -2.54307 | 1.17E-34 | 6.44E-33 |
| RNF2 | 2.100084 | 1.23E-34 | 6.73E-33 |
| HEATR5B /// HEATR5B | 2.055754 | 1.26E-34 | 6.89E-33 |
| CAPN3 | -5.36492 | 1.62E-34 | 8.75E-33 |
| OBSCN | -3.153 | 1.76E-34 | 9.47E-33 |
| ECH1 | -2.12008 | 1.77E-34 | 9.47E-33 |
| POLI | 3.062071 | 1.99E-34 | 1.06E-32 |
| RPL22L1 | 4.623346 | 2.01E-34 | 1.07E-32 |
| FAF2 | 2.007541 | 2.03E-34 | 1.07E-32 |
| DUT | 2.277801 | 2.16E-34 | 1.13E-32 |
| DHFRL1 | 2.104335 | 2.35E-34 | 1.22E-32 |
| ZNF26 | 2.727122 | 2.54E-34 | 1.31E-32 |
| BTG3 | 2.139758 | 2.70E-34 | 1.39E-32 |
| FAM172A | 2.858635 | 2.89E-34 | 1.48E-32 |
| PGBD1 | 2.983546 | 3.01E-34 | 1.53E-32 |
| ASB15 | -2.7899 | 3.25E-34 | 1.64E-32 |
| KIF20B | 3.254531 | 3.29E-34 | 1.66E-32 |
| GPD1 | -2.77519 | 3.66E-34 | 1.84E-32 |
| RBM10 | 2.038458 | 4.15E-34 | 2.07E-32 |
| PYGM | -8.0037 | 4.30E-34 | 2.13E-32 |
| HSPA5 | 2.290625 | 4.37E-34 | 2.16E-32 |
| ARPC3 | 2.026411 | 4.41E-34 | 2.18E-32 |
| ANXA5 | 2.76567 | 4.99E-34 | 2.44E-32 |
| C8orf22 | -7.66334 | 5.03E-34 | 2.45E-32 |
| ZNF784 | -2.20681 | 5.21E-34 | 2.53E-32 |
| ALMS1 | 2.965246 | 5.22E-34 | 2.53E-32 |
| AGO3 | 2.13074 | 5.32E-34 | 2.56E-32 |
| TNAP | 3.435172 | 5.99E-34 | 2.87E-32 |
| ANKRD36B | 5.657584 | 6.62E-34 | 3.14E-32 |
| USP3 | 2.456604 | 6.67E-34 | 3.16E-32 |
| MED17 | 2.184362 | 6.74E-34 | 3.18E-32 |
| LOC101929177 | 2.008253 | 7.06E-34 | 3.32E-32 |
| RPS24 | 2.439484 | 7.92E-34 | 3.70E-32 |
| SAMD4A | -2.28192 | 8.19E-34 | 3.82E-32 |
| PPP4R1 | 2.921585 | 8.57E-34 | 3.97E-32 |
| C9orf173 | -2.18745 | 8.77E-34 | 4.06E-32 |
| WASF1 | 5.101793 | 8.83E-34 | 4.08E-32 |
| SRSF11 | 2.156613 | 9.94E-34 | 4.57E-32 |
| BUB1B | 6.103299 | 1.11E-33 | 5.06E-32 |
| TECRL | -5.04069 | 1.16E-33 | 5.23E-32 |
| ITGB3BP | 3.253138 | 1.19E-33 | 5.34E-32 |
| TCTEX1D2 | 3.462366 | 1.24E-33 | 5.57E-32 |
| SLC25A12 | -2.9484 | 1.36E-33 | 6.06E-32 |
| ATAT1 | 2.777575 | 1.41E-33 | 6.28E-32 |
| ANKRD2 | -5.02483 | 1.43E-33 | 6.35E-32 |
| DHRS7C | -4.83215 | 1.44E-33 | 6.39E-32 |
| ZFP82 | 2.590782 | 1.54E-33 | 6.78E-32 |
| KIAA1586 | 3.184421 | 1.59E-33 | 7.00E-32 |
| F2R | 2.967866 | 1.59E-33 | 7.00E-32 |
| LOC100291323 | 4.238771 | 1.65E-33 | 7.23E-32 |
| SPARC | 2.826063 | 1.74E-33 | 7.59E-32 |
| NDRG2 | -3.1916 | 1.90E-33 | 8.31E-32 |
| CAP1 | 2.835769 | 1.97E-33 | 8.55E-32 |
| ADAR | 2.799772 | 2.00E-33 | 8.65E-32 |
| ATR | 2.279717 | 2.31E-33 | 9.97E-32 |
| PADI2 | -2.23745 | 2.39E-33 | 1.03E-31 |
| ETAA1 | 2.423214 | 3.04E-33 | 1.29E-31 |
| ABRACL | 4.407266 | 3.18E-33 | 1.34E-31 |
| LINC00310 | -2.17315 | 3.53E-33 | 1.49E-31 |
| ZNF677 | 2.508534 | 3.74E-33 | 1.57E-31 |
| FANCD2 | 2.007411 | 4.01E-33 | 1.68E-31 |
| SAR1B | -2.17305 | 4.17E-33 | 1.74E-31 |
| RAB13 | 2.723534 | 4.17E-33 | 1.74E-31 |
| TRIP13 | 4.235911 | 4.61E-33 | 1.92E-31 |
| HDAC1 | 2.531703 | 4.78E-33 | 1.98E-31 |
| ZNF83 | 4.340753 | 5.71E-33 | 2.36E-31 |
| SLC25A5 | 2.978983 | 5.80E-33 | 2.39E-31 |
| GNL2 | 2.943234 | 5.82E-33 | 2.39E-31 |
| SLC30A7 | 2.0125 | 5.83E-33 | 2.40E-31 |
| LDLRAD3 | 4.147412 | 5.94E-33 | 2.44E-31 |
| ZNF813 | 3.101218 | 6.29E-33 | 2.57E-31 |
| CYFIP1 | 2.357392 | 6.39E-33 | 2.60E-31 |
| NUP85 | 2.726448 | 6.42E-33 | 2.61E-31 |
| SLC35A5 | 3.064742 | 7.70E-33 | 3.09E-31 |
| MKNK2 | -2.49838 | 8.15E-33 | 3.26E-31 |
| SLBP | 2.772156 | 8.87E-33 | 3.53E-31 |
| ZNF721 | 2.48031 | 1.06E-32 | 4.19E-31 |
| HPS5 | 2.525656 | 1.17E-32 | 4.60E-31 |
| PTPLAD1 | 2.710033 | 1.17E-32 | 4.60E-31 |
| MGC70870 | 3.034033 | 1.20E-32 | 4.71E-31 |
| NPHP3 | 2.051273 | 1.23E-32 | 4.81E-31 |
| RAB4B | -2.10706 | 1.23E-32 | 4.82E-31 |
| GAMT | -2.85056 | 1.26E-32 | 4.91E-31 |
| EBLN2 | 2.113197 | 1.27E-32 | 4.96E-31 |
| USP13 | -2.67808 | 1.28E-32 | 5.00E-31 |
| TOP2A | 5.998116 | 1.30E-32 | 5.05E-31 |
| RPN2 | 2.456565 | 1.43E-32 | 5.49E-31 |
| SND1-IT1 | 2.782541 | 1.46E-32 | 5.59E-31 |
| CHCHD10 | -3.77773 | 1.52E-32 | 5.79E-31 |
| CMYA5 | -6.60233 | 1.64E-32 | 6.24E-31 |
| RPL7L1 | 2.032927 | 1.64E-32 | 6.24E-31 |
| PPP2R3A | -3.02802 | 1.76E-32 | 6.64E-31 |
| ZNF665 | 2.001038 | 1.98E-32 | 7.45E-31 |
| CEP95 | 2.344327 | 2.41E-32 | 8.98E-31 |
| ACER3 | 2.222501 | 2.43E-32 | 9.02E-31 |
| NCAPG2 | 3.659395 | 2.68E-32 | 9.92E-31 |
| THG1L | 2.517986 | 2.71E-32 | 1.00E-30 |
| ZNF329 | 3.137623 | 2.74E-32 | 1.01E-30 |
| METTL4 | 2.490458 | 2.76E-32 | 1.02E-30 |
| MAPK12 | -2.12475 | 2.80E-32 | 1.03E-30 |
| MARCKS | 3.020955 | 2.81E-32 | 1.03E-30 |
| LOC200609 | 2.179485 | 2.83E-32 | 1.04E-30 |
| HS2ST1 | 2.407997 | 2.87E-32 | 1.05E-30 |
| PAPSS1 | 3.110459 | 2.92E-32 | 1.06E-30 |
| ZNF141 | 2.047498 | 3.02E-32 | 1.10E-30 |
| LMO1 | -2.90611 | 3.53E-32 | 1.28E-30 |
| XBP1 | 2.116152 | 3.69E-32 | 1.33E-30 |
| ZNF587 | 2.697351 | 4.08E-32 | 1.46E-30 |
| BBS10 | 2.487666 | 4.86E-32 | 1.73E-30 |
| HDAC2 | 2.394707 | 4.96E-32 | 1.76E-30 |
| SFXN3 | 2.548669 | 5.54E-32 | 1.96E-30 |
| AFAP1 | 2.993207 | 5.69E-32 | 2.01E-30 |
| OSTC | 2.303099 | 6.13E-32 | 2.16E-30 |
| GGH | 5.212192 | 6.34E-32 | 2.23E-30 |
| CEP44 | 2.380291 | 6.61E-32 | 2.31E-30 |
| TMEM123 | 3.100313 | 6.87E-32 | 2.40E-30 |
| RRM2 | 6.02672 | 7.07E-32 | 2.46E-30 |
| SSR2 | 2.413223 | 7.42E-32 | 2.58E-30 |
| SNRPB2 | 2.405564 | 8.08E-32 | 2.80E-30 |
| ELOVL6 | 2.811271 | 9.10E-32 | 3.14E-30 |
| MXRA5 | 4.919373 | 9.37E-32 | 3.22E-30 |
| SLC25A13 | 2.004024 | 9.45E-32 | 3.24E-30 |
| EML4 | 2.515454 | 1.02E-31 | 3.49E-30 |
| SLX4IP | 2.89728 | 1.06E-31 | 3.62E-30 |
| CKAP4 | 2.356698 | 1.07E-31 | 3.62E-30 |
| LINC01420 | 2.33254 | 1.08E-31 | 3.66E-30 |
| LOC100132891 | 4.105301 | 1.09E-31 | 3.67E-30 |
| ZBTB16 | -5.1873 | 1.10E-31 | 3.72E-30 |
| NBPF20 | 2.978008 | 1.18E-31 | 3.96E-30 |
| EIF4A3 | 2.453315 | 1.26E-31 | 4.21E-30 |
| SSBP1 | 2.017587 | 1.36E-31 | 4.52E-30 |
| PSMC3IP | 2.175196 | 1.48E-31 | 4.92E-30 |
| MIS18A | 3.26944 | 1.56E-31 | 5.13E-30 |
| HAUS1 | 3.898374 | 1.57E-31 | 5.15E-30 |
| SASS6 | 2.734594 | 1.72E-31 | 5.63E-30 |
| GAK | 2.018404 | 1.73E-31 | 5.66E-30 |
| BMP2K | 2.206625 | 1.73E-31 | 5.66E-30 |
| OR7E47P | -2.3104 | 1.75E-31 | 5.71E-30 |
| BNIP3L | 2.705871 | 1.75E-31 | 5.71E-30 |
| ZDHHC13 | 2.308195 | 1.80E-31 | 5.83E-30 |
| COX7A2L | 2.206297 | 1.81E-31 | 5.85E-30 |
| PGM1 | -3.18466 | 2.01E-31 | 6.49E-30 |
| LOC100134445 | 3.400393 | 2.06E-31 | 6.64E-30 |
| PTP4A1 | -2.04768 | 2.13E-31 | 6.83E-30 |
| CCNB1 | 5.49965 | 2.13E-31 | 6.83E-30 |
| ADNP2 | 2.468866 | 2.29E-31 | 7.31E-30 |
| FBXO32 | -3.80606 | 2.34E-31 | 7.45E-30 |
| HMGXB4 | 2.512489 | 2.38E-31 | 7.57E-30 |
| KPNA2 | 3.944656 | 2.65E-31 | 8.40E-30 |
| SMARCAD1 | 2.194616 | 2.74E-31 | 8.67E-30 |
| CHN1 | 3.422478 | 2.88E-31 | 9.10E-30 |
| PHYH | -3.34942 | 3.20E-31 | 1.00E-29 |
| RBM3 | 2.886796 | 3.47E-31 | 1.09E-29 |
| CYSTM1 | -3.60791 | 3.48E-31 | 1.09E-29 |
| ZNF512 | 2.320997 | 3.51E-31 | 1.10E-29 |
| MAPK7 | 2.003859 | 3.68E-31 | 1.14E-29 |
| THOC1 | 2.327612 | 3.80E-31 | 1.18E-29 |
| SACM1L | 2.54007 | 3.97E-31 | 1.23E-29 |
| GATC | 2.35583 | 4.19E-31 | 1.29E-29 |
| EZH2 | 5.305021 | 4.26E-31 | 1.31E-29 |
| TUBB | 2.514272 | 4.37E-31 | 1.34E-29 |
| GAS2L3 | 3.710538 | 4.48E-31 | 1.37E-29 |
| MED29 | 2.487037 | 4.59E-31 | 1.40E-29 |
| TMF1 | 2.064576 | 4.60E-31 | 1.41E-29 |
| FLJ35934 | 2.245527 | 4.94E-31 | 1.50E-29 |
| XXYLT1 | 2.483725 | 5.09E-31 | 1.55E-29 |
| DPYSL2 | 2.757083 | 5.16E-31 | 1.57E-29 |
| VDAC1 | -2.0009 | 5.52E-31 | 1.67E-29 |
| SUCO | 3.763871 | 5.95E-31 | 1.79E-29 |
| DDX39A | 3.503199 | 6.29E-31 | 1.88E-29 |
| TSEN34 | 2.156838 | 6.33E-31 | 1.89E-29 |
| ZNF430 | 2.721118 | 6.42E-31 | 1.91E-29 |
| ZNF680 | 3.47164 | 6.43E-31 | 1.91E-29 |
| CEP78 | 2.739711 | 6.44E-31 | 1.91E-29 |
| KLHL42 | 2.471333 | 7.05E-31 | 2.08E-29 |
| RPL31 | 2.735875 | 7.44E-31 | 2.19E-29 |
| SFXN1 | 2.384473 | 7.50E-31 | 2.20E-29 |
| PGAM1 | 2.768612 | 7.62E-31 | 2.23E-29 |
| VCPKMT | 2.206581 | 7.76E-31 | 2.27E-29 |
| DEXI | -2.14894 | 9.22E-31 | 2.68E-29 |
| PRTFDC1 | 4.895648 | 9.36E-31 | 2.71E-29 |
| VGLL3 | 5.886036 | 9.67E-31 | 2.80E-29 |
| SMTNL1 | -4.28585 | 9.90E-31 | 2.86E-29 |
| RFC4 | 3.356455 | 1.14E-30 | 3.27E-29 |
| TRIM54 | -3.30548 | 1.16E-30 | 3.32E-29 |
| PPIB | 3.009696 | 1.18E-30 | 3.36E-29 |
| PET100 | 2.102636 | 1.20E-30 | 3.41E-29 |
| ZFP69 | 2.525119 | 1.24E-30 | 3.54E-29 |
| RASA2 | 2.84621 | 1.25E-30 | 3.55E-29 |
| NDC80 | 5.213689 | 1.25E-30 | 3.56E-29 |
| ANKRD36 | 3.207784 | 1.27E-30 | 3.59E-29 |
| PPTC7 | -2.20587 | 1.29E-30 | 3.66E-29 |
| WDR36 | 2.224587 | 1.36E-30 | 3.84E-29 |
| CEP192 | 2.449591 | 1.38E-30 | 3.89E-29 |
| TTC14 | 2.78732 | 1.39E-30 | 3.90E-29 |
| PRC1 | 4.728364 | 1.41E-30 | 3.96E-29 |
| ACHE | -2.58916 | 1.44E-30 | 4.03E-29 |
| VRK1 | 3.709788 | 1.45E-30 | 4.05E-29 |
| KNTC1 | 4.077149 | 1.55E-30 | 4.31E-29 |
| STIL | 3.782106 | 1.58E-30 | 4.39E-29 |
| NIPSNAP3B | -4.1958 | 1.59E-30 | 4.40E-29 |
| MASTL | 3.874927 | 1.59E-30 | 4.40E-29 |
| ASAP1-IT2 | 2.732371 | 1.63E-30 | 4.49E-29 |
| TMTC3 | 2.260815 | 1.65E-30 | 4.54E-29 |
| ASAP1-IT1 | 2.320884 | 1.70E-30 | 4.66E-29 |
| PRPSAP2 | 2.073517 | 1.81E-30 | 4.95E-29 |
| UGGT2 | 2.128294 | 1.83E-30 | 4.99E-29 |
| SMC4 | 3.617791 | 1.95E-30 | 5.31E-29 |
| ATP2A1 | -4.09619 | 2.00E-30 | 5.43E-29 |
| RPS21 | 2.325141 | 2.01E-30 | 5.46E-29 |
| GINS1 | 5.618679 | 2.03E-30 | 5.49E-29 |
| ASB12 | -2.2345 | 2.08E-30 | 5.62E-29 |
| CDC25B | 3.178048 | 2.26E-30 | 6.10E-29 |
| MID1 | 3.884959 | 2.27E-30 | 6.12E-29 |
| CNTRL | 2.557625 | 2.28E-30 | 6.13E-29 |
| COL3A1 | 4.281936 | 2.31E-30 | 6.21E-29 |
| MYH1 | -8.04991 | 2.47E-30 | 6.61E-29 |
| DBF4 | 4.419515 | 2.47E-30 | 6.61E-29 |
| C5orf28 | 2.51832 | 2.59E-30 | 6.90E-29 |
| PRKAA2 | -4.16628 | 2.66E-30 | 7.06E-29 |
| CELF2 | 2.982329 | 2.78E-30 | 7.35E-29 |
| PON2 | 2.680372 | 2.81E-30 | 7.42E-29 |
| PTGFRN | 3.655533 | 3.01E-30 | 7.90E-29 |
| RBPJ | 2.898206 | 3.09E-30 | 8.11E-29 |
| CLIC1 | 2.594967 | 3.09E-30 | 8.11E-29 |
| COL5A2 | 4.302556 | 3.13E-30 | 8.19E-29 |
| GLT8D1 | 2.285621 | 3.18E-30 | 8.32E-29 |
| EXTL1 | -2.10215 | 3.25E-30 | 8.49E-29 |
| SMA4 | 3.877996 | 3.26E-30 | 8.51E-29 |
| ACADSB | -2.00407 | 3.34E-30 | 8.70E-29 |
| ZNF558 | 2.731918 | 3.42E-30 | 8.88E-29 |
| CALR | 2.079899 | 3.93E-30 | 1.02E-28 |
| GAR1 | 2.26443 | 4.85E-30 | 1.24E-28 |
| HMGCS1 | 2.64669 | 4.95E-30 | 1.26E-28 |
| TRAF5 | 2.079783 | 5.03E-30 | 1.28E-28 |
| VIM | 3.51828 | 5.06E-30 | 1.29E-28 |
| RORC | -2.06413 | 5.22E-30 | 1.33E-28 |
| SERINC5 | 3.730206 | 5.39E-30 | 1.37E-28 |
| RAPGEF6 | 2.610209 | 5.75E-30 | 1.46E-28 |
| RSPO3 | -4.44755 | 6.07E-30 | 1.53E-28 |
| CYP4B1 | -2.12407 | 6.68E-30 | 1.68E-28 |
| TSHZ3 | 3.473916 | 6.92E-30 | 1.73E-28 |
| COL1A2 | 4.345472 | 7.04E-30 | 1.76E-28 |
| AP3S1 | 2.136124 | 7.32E-30 | 1.83E-28 |
| SNX30 | 2.340233 | 7.54E-30 | 1.88E-28 |
| CDK1 | 4.102303 | 7.66E-30 | 1.90E-28 |
| PAIP2B | -3.07664 | 7.76E-30 | 1.92E-28 |
| ZNF260 | 3.405683 | 7.85E-30 | 1.94E-28 |
| ZNF85 | 2.357669 | 8.06E-30 | 1.99E-28 |
| TMEM159 | -3.12627 | 8.60E-30 | 2.11E-28 |
| PCNA | 3.204968 | 8.87E-30 | 2.17E-28 |
| ZCCHC7 | 2.672465 | 9.49E-30 | 2.31E-28 |
| DUSP12 | 2.449994 | 1.03E-29 | 2.51E-28 |
| APEX1 | 2.078321 | 1.29E-29 | 3.11E-28 |
| RAD9A | 2.185783 | 1.29E-29 | 3.12E-28 |
| HSPB7 | -3.13799 | 1.30E-29 | 3.13E-28 |
| MDM1 | 2.81193 | 1.30E-29 | 3.13E-28 |
| CXCR4 | 4.292822 | 1.43E-29 | 3.44E-28 |
| RFWD3 | 3.438047 | 1.48E-29 | 3.55E-28 |
| ATAD2 | 4.348772 | 1.50E-29 | 3.60E-28 |
| FBXO5 | 4.371087 | 1.51E-29 | 3.61E-28 |
| TCERG1 | 2.647366 | 1.55E-29 | 3.68E-28 |
| FANCI | 3.406523 | 1.68E-29 | 3.97E-28 |
| MINPP1 | 2.491848 | 1.73E-29 | 4.10E-28 |
| PGAP1 | 2.972981 | 1.79E-29 | 4.24E-28 |
| RECQL | 2.315481 | 1.92E-29 | 4.52E-28 |
| N4BP2 | 3.28219 | 1.95E-29 | 4.59E-28 |
| PRPS2 | 3.377788 | 1.96E-29 | 4.61E-28 |
| ZNF43 | 4.498645 | 2.19E-29 | 5.11E-28 |
| ZNF137P | 2.734418 | 2.21E-29 | 5.15E-28 |
| WDR11 | 2.700878 | 2.26E-29 | 5.24E-28 |
| CNTLN | 2.258095 | 2.35E-29 | 5.43E-28 |
| RNF123 | -2.28416 | 2.43E-29 | 5.60E-28 |
| MYH2 | -9.16461 | 2.43E-29 | 5.60E-28 |
| CCDC138 | 2.891592 | 2.45E-29 | 5.64E-28 |
| SPDL1 | 2.992441 | 2.70E-29 | 6.15E-28 |
| SNAI3 | -2.30017 | 2.80E-29 | 6.38E-28 |
| HSP90B1 | 2.584907 | 2.82E-29 | 6.41E-28 |
| CENPK | 5.73224 | 2.89E-29 | 6.56E-28 |
| RAP1B | 2.626155 | 3.05E-29 | 6.92E-28 |
| ZNF506 | 2.151875 | 3.18E-29 | 7.16E-28 |
| NBPF1 | 3.775224 | 3.23E-29 | 7.25E-28 |
| SPC24 | 2.220201 | 3.30E-29 | 7.39E-28 |
| BCO2 | -2.16482 | 3.32E-29 | 7.44E-28 |
| LINC00621 | 2.139352 | 3.36E-29 | 7.51E-28 |
| DYNC2H1 | 2.151428 | 3.55E-29 | 7.91E-28 |
| PDIA4 | 2.276705 | 3.92E-29 | 8.70E-28 |
| UBAC1 | -2.08732 | 3.96E-29 | 8.77E-28 |
| THOC2 | 2.261745 | 4.25E-29 | 9.38E-28 |
| APP | 2.452226 | 4.35E-29 | 9.58E-28 |
| CEL | 2.710003 | 4.36E-29 | 9.60E-28 |
| TGIF2 | 2.879991 | 4.63E-29 | 1.02E-27 |
| PFAS | 2.850801 | 4.66E-29 | 1.02E-27 |
| KIF20A | 5.129845 | 4.66E-29 | 1.02E-27 |
| NKTR | 2.027442 | 4.70E-29 | 1.03E-27 |
| H2AFZ | 2.235904 | 5.03E-29 | 1.09E-27 |
| AMPD1 | -7.60672 | 5.23E-29 | 1.14E-27 |
| DNMT1 | 3.458203 | 5.52E-29 | 1.19E-27 |
| C8orf59 | 2.993819 | 6.13E-29 | 1.32E-27 |
| BRCA2 | 2.989603 | 6.14E-29 | 1.32E-27 |
| SNORA72 | 3.595632 | 6.25E-29 | 1.34E-27 |
| ZNF217 | 3.769582 | 6.43E-29 | 1.38E-27 |
| CTBP2 | 2.388056 | 6.72E-29 | 1.44E-27 |
| SLC37A3 | 2.970763 | 7.15E-29 | 1.52E-27 |
| C14orf93 | 2.192713 | 7.45E-29 | 1.58E-27 |
| SGOL2 | 5.058164 | 7.75E-29 | 1.64E-27 |
| ZNF266 | 2.659225 | 7.89E-29 | 1.66E-27 |
| MUM1 | 2.27502 | 8.24E-29 | 1.74E-27 |
| TAF1B | 2.253634 | 8.54E-29 | 1.80E-27 |
| CCAR1 | 2.243413 | 8.58E-29 | 1.80E-27 |
| ALG8 | 2.296572 | 8.67E-29 | 1.82E-27 |
| ZMYM1 | 2.309602 | 8.84E-29 | 1.85E-27 |
| C16orf80 | 2.159131 | 8.93E-29 | 1.87E-27 |
| ANLN | 4.962196 | 1.00E-28 | 2.08E-27 |
| HSD17B7 | 2.056334 | 1.03E-28 | 2.14E-27 |
| DPY19L3 | 3.65909 | 1.06E-28 | 2.19E-27 |
| HDGFRP3 | 2.103816 | 1.06E-28 | 2.20E-27 |
| DIS3L2 | 2.204858 | 1.07E-28 | 2.21E-27 |
| ECT2 | 2.511746 | 1.09E-28 | 2.26E-27 |
| ZNF521 | 4.89368 | 1.14E-28 | 2.36E-27 |
| ETNK1 | 2.212904 | 1.18E-28 | 2.42E-27 |
| CEP162 | 2.072252 | 1.24E-28 | 2.55E-27 |
| MARCKSL1 | 3.518334 | 1.24E-28 | 2.55E-27 |
| CENPM | 3.047464 | 1.26E-28 | 2.58E-27 |
| ADPRHL1 | -2.61068 | 1.27E-28 | 2.59E-27 |
| PCDHB9 | 2.557689 | 1.27E-28 | 2.60E-27 |
| CCNL1 | 2.185598 | 1.30E-28 | 2.65E-27 |
| ART3 | -5.41426 | 1.36E-28 | 2.77E-27 |
| KIF3A | 2.147134 | 1.37E-28 | 2.77E-27 |
| CACNA1S | -4.55321 | 1.44E-28 | 2.92E-27 |
| TMEM136 | 2.168601 | 1.45E-28 | 2.93E-27 |
| CENPL | 3.202842 | 1.59E-28 | 3.21E-27 |
| CAND1 | 2.867149 | 1.60E-28 | 3.22E-27 |
| CENPU | 3.876376 | 1.62E-28 | 3.24E-27 |
| PAN3 | 3.016301 | 1.63E-28 | 3.26E-27 |
| CEP152 | 2.562199 | 1.63E-28 | 3.26E-27 |
| LMOD2 | -7.22789 | 1.91E-28 | 3.81E-27 |
| ALKBH2 | 2.352393 | 1.93E-28 | 3.84E-27 |
| CENPE | 4.605694 | 1.97E-28 | 3.91E-27 |
| USP1 | 3.031794 | 2.11E-28 | 4.17E-27 |
| LIG1 | 2.810871 | 2.40E-28 | 4.73E-27 |
| SRSF7 | 2.037001 | 2.61E-28 | 5.13E-27 |
| SHKBP1 | 2.081184 | 2.63E-28 | 5.16E-27 |
| COL5A1 | 3.950333 | 2.73E-28 | 5.35E-27 |
| SHISA4 | -2.41243 | 3.12E-28 | 6.09E-27 |
| USP11 | 2.015914 | 3.32E-28 | 6.46E-27 |
| ETFDH | -2.3793 | 3.39E-28 | 6.59E-27 |
| IRF2BPL | 2.016619 | 3.79E-28 | 7.31E-27 |
| SCAMP1-AS1 | 2.042166 | 3.90E-28 | 7.51E-27 |
| LOC100132352 | 2.175723 | 4.03E-28 | 7.77E-27 |
| FXYD1 | -4.38332 | 4.05E-28 | 7.79E-27 |
| TTC13 | 2.690809 | 4.06E-28 | 7.81E-27 |
| ADHFE1 | -2.40253 | 4.08E-28 | 7.84E-27 |
| NIPBL | 2.177389 | 4.15E-28 | 7.97E-27 |
| NIP7 | 2.385102 | 4.19E-28 | 8.03E-27 |
| ARHGEF18 | 2.293761 | 4.26E-28 | 8.16E-27 |
| XIRP1 | -4.69629 | 4.28E-28 | 8.19E-27 |
| CSK | 2.423295 | 4.29E-28 | 8.20E-27 |
| MBTD1 | 2.751716 | 4.35E-28 | 8.30E-27 |
| CPSF3 | 2.905483 | 4.94E-28 | 9.35E-27 |
| ZNF785 | 2.480227 | 4.97E-28 | 9.39E-27 |
| XRCC4 | 2.604 | 5.11E-28 | 9.65E-27 |
| SCGB1D2 | -2.46249 | 5.18E-28 | 9.76E-27 |
| CCDC69 | -4.52786 | 5.54E-28 | 1.04E-26 |
| SNRNP200 | 2.762404 | 5.93E-28 | 1.11E-26 |
| YBX3 | -2.43847 | 6.08E-28 | 1.14E-26 |
| POPDC2 | -3.32523 | 6.13E-28 | 1.15E-26 |
| MIS18BP1 | 2.46457 | 6.18E-28 | 1.16E-26 |
| SPRED1 | 2.956943 | 6.26E-28 | 1.17E-26 |
| C2orf43 | 2.617242 | 6.35E-28 | 1.18E-26 |
| ERI1 | 2.608759 | 6.55E-28 | 1.22E-26 |
| CEP76 | 2.973155 | 6.56E-28 | 1.22E-26 |
| IDH2 | -2.51018 | 6.76E-28 | 1.26E-26 |
| DNMT3B | 3.643724 | 6.82E-28 | 1.27E-26 |
| GTF2H3 | 2.301562 | 7.66E-28 | 1.42E-26 |
| HELLS | 3.62222 | 7.74E-28 | 1.43E-26 |
| PTTG1 | 4.514788 | 7.86E-28 | 1.45E-26 |
| NCAPG | 5.160134 | 8.16E-28 | 1.50E-26 |
| CCDC15 | 3.693155 | 8.89E-28 | 1.63E-26 |
| INTS2 | 2.544486 | 8.91E-28 | 1.64E-26 |
| WDFY2 | 2.60739 | 8.94E-28 | 1.64E-26 |
| AMMECR1L | 2.498997 | 9.22E-28 | 1.69E-26 |
| MIR34A | 3.43439 | 9.46E-28 | 1.73E-26 |
| PSAT1 | 4.098754 | 9.79E-28 | 1.79E-26 |
| LOC101928054 | 2.142117 | 9.79E-28 | 1.79E-26 |
| MAP4K4 | 3.147006 | 1.00E-27 | 1.82E-26 |
| MAP9 | 2.477365 | 1.03E-27 | 1.87E-26 |
| PTTG3P | 2.558853 | 1.13E-27 | 2.05E-26 |
| PLK4 | 3.025955 | 1.14E-27 | 2.06E-26 |
| PBK | 6.137825 | 1.20E-27 | 2.16E-26 |
| XPOT | 2.329777 | 1.24E-27 | 2.23E-26 |
| CD81 | 2.4651 | 1.25E-27 | 2.25E-26 |
| TP53I3 | 3.150599 | 1.28E-27 | 2.30E-26 |
| KCNJ11 | -2.11774 | 1.29E-27 | 2.30E-26 |
| TRIM63 | -5.43996 | 1.29E-27 | 2.31E-26 |
| PFKM | -3.07951 | 1.29E-27 | 2.32E-26 |
| NUDT5 | 2.362524 | 1.30E-27 | 2.33E-26 |
| UBE2G1 | -2.20059 | 1.37E-27 | 2.43E-26 |
| ZNF292 | 2.127812 | 1.37E-27 | 2.44E-26 |
| DBN1 | 2.62004 | 1.41E-27 | 2.50E-26 |
| OPTN | -3.9493 | 1.44E-27 | 2.55E-26 |
| SMC6 | 2.921084 | 1.54E-27 | 2.73E-26 |
| ALDOA | -2.73183 | 1.55E-27 | 2.74E-26 |
| RALGPS2 | 3.237974 | 1.64E-27 | 2.89E-26 |
| PLN | -6.93994 | 1.70E-27 | 2.99E-26 |
| CFDP1 | 2.459739 | 1.76E-27 | 3.09E-26 |
| DZIP1 | 3.313695 | 1.83E-27 | 3.19E-26 |
| ANKRD50 | 2.436953 | 1.85E-27 | 3.23E-26 |
| ARL5B | 2.459728 | 1.89E-27 | 3.28E-26 |
| ZNF121 | 3.12186 | 1.90E-27 | 3.31E-26 |
| TPM2 | -2.58873 | 1.94E-27 | 3.38E-26 |
| FBL | 2.741851 | 1.98E-27 | 3.43E-26 |
| HNRNPA3 | 2.491906 | 2.04E-27 | 3.52E-26 |
| TMEM98 | 3.169226 | 2.14E-27 | 3.68E-26 |
| PRKX | 3.844835 | 2.15E-27 | 3.70E-26 |
| FAM13B | 2.471241 | 2.20E-27 | 3.77E-26 |
| CBX3 | 3.07077 | 2.32E-27 | 3.97E-26 |
| GNB4 | 2.249284 | 2.45E-27 | 4.19E-26 |
| DTNA | -2.58346 | 2.65E-27 | 4.51E-26 |
| DMTF1 | 2.59387 | 2.71E-27 | 4.61E-26 |
| TSPAN6 | 2.888276 | 2.71E-27 | 4.62E-26 |
| FABP3 | -4.77956 | 2.73E-27 | 4.63E-26 |
| TOPBP1 | 2.452525 | 2.74E-27 | 4.66E-26 |
| ZNF12 | 2.399082 | 2.78E-27 | 4.71E-26 |
| ASAP1 | 2.411072 | 2.81E-27 | 4.75E-26 |
| STK39 | 3.57604 | 2.82E-27 | 4.76E-26 |
| HNRNPA1 | 2.541298 | 2.83E-27 | 4.78E-26 |
| NOL11 | 2.256905 | 2.87E-27 | 4.84E-26 |
| PTPN3 | -2.73635 | 2.95E-27 | 4.97E-26 |
| POGZ | 2.512341 | 3.04E-27 | 5.11E-26 |
| PARP4 | 2.227471 | 3.06E-27 | 5.15E-26 |
| MAGED1 | 2.439375 | 3.11E-27 | 5.23E-26 |
| TDG | 3.47375 | 3.29E-27 | 5.51E-26 |
| DTL | 5.123976 | 3.29E-27 | 5.52E-26 |
| FOXM1 | 4.476214 | 3.32E-27 | 5.56E-26 |
| PTGES3L | -3.34328 | 3.42E-27 | 5.71E-26 |
| ARHGEF10 | 2.884548 | 3.49E-27 | 5.81E-26 |
| KBTBD12 | -4.52419 | 3.51E-27 | 5.84E-26 |
| KIF11 | 5.338751 | 3.62E-27 | 6.02E-26 |
| DDX55 | 2.243798 | 3.63E-27 | 6.04E-26 |
| XPO1 | 2.402897 | 3.66E-27 | 6.08E-26 |
| ACTL6A | 2.873951 | 3.72E-27 | 6.16E-26 |
| ANAPC4 | 2.195448 | 3.72E-27 | 6.16E-26 |
| SULF2 | 4.283524 | 3.94E-27 | 6.51E-26 |
| C8orf88 | 3.774526 | 3.97E-27 | 6.56E-26 |
| NCAPD3 | 2.512443 | 4.04E-27 | 6.66E-26 |
| SAT1 | 3.244501 | 4.17E-27 | 6.86E-26 |
| FANCG | 2.886619 | 4.32E-27 | 7.10E-26 |
| UBE2QL1 | -4.23834 | 4.32E-27 | 7.10E-26 |
| RAD18 | 2.12252 | 4.45E-27 | 7.30E-26 |
| NASP | 2.74646 | 4.93E-27 | 8.03E-26 |
| NDC1 | 2.447666 | 4.97E-27 | 8.10E-26 |
| TPD52L1 | -4.16276 | 5.30E-27 | 8.61E-26 |
| SYNM | -5.24056 | 5.68E-27 | 9.20E-26 |
| SETD2 | 2.142534 | 5.75E-27 | 9.31E-26 |
| HIST1H4J | 2.177766 | 6.04E-27 | 9.75E-26 |
| TPX2 | 4.758681 | 6.18E-27 | 9.97E-26 |
| CSTF3 | 2.50635 | 6.66E-27 | 1.07E-25 |
| GAL3ST4 | 2.313058 | 6.89E-27 | 1.11E-25 |
| SSB | 2.45686 | 7.39E-27 | 1.18E-25 |
| MBIP | 2.26412 | 7.83E-27 | 1.25E-25 |
| WRN | 3.227733 | 7.91E-27 | 1.26E-25 |
| SLC25A11 | -2.12094 | 7.95E-27 | 1.27E-25 |
| SKA2 | 2.620896 | 8.18E-27 | 1.30E-25 |
| TMEM167A | 2.150156 | 9.12E-27 | 1.44E-25 |
| DMXL2 | 2.694977 | 9.88E-27 | 1.56E-25 |
| JPH1 | -3.80597 | 9.88E-27 | 1.56E-25 |
| SRI | 2.221768 | 9.97E-27 | 1.57E-25 |
| PRKCQ | -4.37071 | 1.00E-26 | 1.57E-25 |
| CDCA7 | 3.762958 | 1.01E-26 | 1.59E-25 |
| LINC01405 | -6.22557 | 1.06E-26 | 1.66E-25 |
| SNORA21 | 3.284069 | 1.07E-26 | 1.68E-25 |
| CETN3 | 2.051086 | 1.09E-26 | 1.71E-25 |
| ZNF382 | 2.014558 | 1.11E-26 | 1.73E-25 |
| SLC20A1 | 3.102336 | 1.20E-26 | 1.87E-25 |
| MIRLET7D | 2.512598 | 1.21E-26 | 1.88E-25 |
| CCNG2 | 2.675539 | 1.23E-26 | 1.91E-25 |
| CCDC88A | 2.466359 | 1.30E-26 | 2.01E-25 |
| CEP55 | 4.679274 | 1.31E-26 | 2.02E-25 |
| TRDN | -7.22993 | 1.36E-26 | 2.10E-25 |
| GORAB | 3.157323 | 1.38E-26 | 2.13E-25 |
| SPAG5 | 4.032926 | 1.38E-26 | 2.13E-25 |
| FAM216A | 2.949569 | 1.41E-26 | 2.18E-25 |
| NPY6R | -3.50196 | 1.44E-26 | 2.21E-25 |
| EED | 2.773096 | 1.55E-26 | 2.38E-25 |
| LOC283737 | -2.85855 | 1.66E-26 | 2.54E-25 |
| MIB1 | 3.059689 | 1.73E-26 | 2.64E-25 |
| POLE2 | 3.376225 | 1.80E-26 | 2.73E-25 |
| AMPD2 | 2.694524 | 1.80E-26 | 2.74E-25 |
| GYG1 | -2.89238 | 1.87E-26 | 2.83E-25 |
| KIF23 | 4.64462 | 1.87E-26 | 2.84E-25 |
| NHLRC3 | 2.019839 | 2.04E-26 | 3.09E-25 |
| SOX4 | 2.611029 | 2.27E-26 | 3.42E-25 |
| RAD54L | 3.07203 | 2.58E-26 | 3.87E-25 |
| NUP160 | 2.188376 | 2.67E-26 | 3.99E-25 |
| LOC284023 | 2.078548 | 2.69E-26 | 4.02E-25 |
| PCDH7 | 3.037914 | 2.82E-26 | 4.21E-25 |
| MTMR4 | 2.363954 | 2.83E-26 | 4.22E-25 |
| CXCR2 | -2.05786 | 2.98E-26 | 4.42E-25 |
| DUSP3 | -2.78642 | 3.03E-26 | 4.49E-25 |
| ZNF616 | 2.231049 | 3.14E-26 | 4.66E-25 |
| FBXL7 | 2.729233 | 3.29E-26 | 4.86E-25 |
| DDX27 | 2.048668 | 3.37E-26 | 4.96E-25 |
| LAMB1 | 3.92632 | 3.38E-26 | 4.98E-25 |
| AHSA2 | 2.376817 | 3.61E-26 | 5.31E-25 |
| PGM2 | 2.253284 | 3.78E-26 | 5.55E-25 |
| PHF5A | 2.02188 | 3.80E-26 | 5.57E-25 |
| WDR34 | 2.083061 | 3.82E-26 | 5.59E-25 |
| LOC101060691 | 2.644874 | 3.87E-26 | 5.66E-25 |
| EXT2 | 2.219045 | 3.90E-26 | 5.70E-25 |
| SEC24D | 3.098696 | 3.91E-26 | 5.71E-25 |
| ZNF667 | 2.599508 | 4.02E-26 | 5.86E-25 |
| PINK1 | -2.52801 | 4.43E-26 | 6.41E-25 |
| GINS3 | 2.163453 | 4.67E-26 | 6.73E-25 |
| TIPIN | 2.767283 | 4.84E-26 | 6.96E-25 |
| ANKRD13A | 2.992395 | 4.90E-26 | 7.04E-25 |
| CCNT2 | 2.072281 | 4.93E-26 | 7.08E-25 |
| CHD1 | 2.475566 | 5.11E-26 | 7.33E-25 |
| BTG1 | 2.045681 | 5.27E-26 | 7.54E-25 |
| ACTN3 | -5.5521 | 5.58E-26 | 7.94E-25 |
| TTK | 5.342579 | 5.64E-26 | 8.00E-25 |
| GMCL1 | 2.075704 | 5.74E-26 | 8.13E-25 |
| SRD5A1 | 2.321993 | 6.14E-26 | 8.65E-25 |
| EXOC1 | 2.070242 | 6.25E-26 | 8.79E-25 |
| ZNF788 /// ZNF788 | 2.192213 | 6.27E-26 | 8.81E-25 |
| NAP1L1 | 2.433924 | 6.48E-26 | 9.09E-25 |
| CDC7 | 4.422775 | 6.49E-26 | 9.09E-25 |
| HACE1 | 2.928222 | 6.72E-26 | 9.41E-25 |
| PDK2 | -2.12828 | 6.96E-26 | 9.74E-25 |
| CCZ1B | 2.41015 | 7.01E-26 | 9.80E-25 |
| TANC1 | 2.000017 | 7.34E-26 | 1.02E-24 |
| TRIP4 | 2.104914 | 7.37E-26 | 1.03E-24 |
| TCF12 | 2.332862 | 7.41E-26 | 1.03E-24 |
| MEST | 6.293321 | 7.46E-26 | 1.04E-24 |
| CLK2 | 2.177516 | 7.75E-26 | 1.07E-24 |
| SSBP3-AS1 | 3.081134 | 7.87E-26 | 1.09E-24 |
| CHEK2 | 2.838477 | 7.95E-26 | 1.10E-24 |
| SMIM15 | 2.06287 | 8.09E-26 | 1.12E-24 |
| UBTD2 | 2.50099 | 8.26E-26 | 1.14E-24 |
| NUF2 | 5.99527 | 8.54E-26 | 1.17E-24 |
| ZNF781 | 3.082247 | 8.96E-26 | 1.23E-24 |
| CKAP2 | 2.019477 | 9.01E-26 | 1.23E-24 |
| CDC20 | 4.133373 | 9.03E-26 | 1.24E-24 |
| COMMD2 | 2.256085 | 9.13E-26 | 1.25E-24 |
| BDP1 | 2.442151 | 9.19E-26 | 1.26E-24 |
| LOC728392 | 2.827168 | 9.20E-26 | 1.26E-24 |
| BBX | 2.300293 | 9.37E-26 | 1.28E-24 |
| MYH10 | 3.394354 | 9.43E-26 | 1.28E-24 |
| PPIL4 | 2.62228 | 9.50E-26 | 1.29E-24 |
| ZNF251 | 2.211052 | 9.85E-26 | 1.34E-24 |
| NUMB | 2.261773 | 1.04E-25 | 1.41E-24 |
| POT1 | 2.419955 | 1.06E-25 | 1.43E-24 |
| HERPUD2 | 2.009622 | 1.09E-25 | 1.47E-24 |
| MNS1 | 3.234275 | 1.14E-25 | 1.53E-24 |
| KDELC1 | 2.993595 | 1.17E-25 | 1.57E-24 |
| PRKCQ-AS1 | -2.24133 | 1.22E-25 | 1.64E-24 |
| C11orf80 | 2.357902 | 1.29E-25 | 1.73E-24 |
| MICAL1 | 3.709152 | 1.32E-25 | 1.77E-24 |
| NAT14 | 2.856946 | 1.35E-25 | 1.81E-24 |
| UGCG | 3.787682 | 1.36E-25 | 1.82E-24 |
| ZNF738 | 3.484011 | 1.44E-25 | 1.92E-24 |
| LMOD3 | -4.93596 | 1.47E-25 | 1.95E-24 |
| ZNF566 | 2.591791 | 1.50E-25 | 1.99E-24 |
| ORC6 | 3.735543 | 1.55E-25 | 2.05E-24 |
| DCAF13 | 2.064613 | 1.56E-25 | 2.06E-24 |
| UBLCP1 | 2.778576 | 1.56E-25 | 2.06E-24 |
| PARVB | -2.12329 | 1.59E-25 | 2.10E-24 |
| ACADVL | -2.16999 | 1.69E-25 | 2.23E-24 |
| ARID2 | 2.623182 | 1.69E-25 | 2.23E-24 |
| MTF2 | 2.380867 | 1.81E-25 | 2.38E-24 |
| WDR54 | 2.373895 | 1.83E-25 | 2.40E-24 |
| PHF14 | 2.315085 | 1.87E-25 | 2.45E-24 |
| RNF24 | 2.584257 | 1.88E-25 | 2.46E-24 |
| LOC284825 | -2.27256 | 1.92E-25 | 2.52E-24 |
| GOLGA8A | 2.713481 | 1.99E-25 | 2.59E-24 |
| CCDC104 | 2.609998 | 2.00E-25 | 2.61E-24 |
| LOC389765 | 2.66901 | 2.01E-25 | 2.63E-24 |
| GNB1 | 2.113267 | 2.05E-25 | 2.66E-24 |
| LUC7L | 2.113248 | 2.13E-25 | 2.77E-24 |
| CTNNA3 | -2.11714 | 2.17E-25 | 2.81E-24 |
| AADAT | 2.910866 | 2.17E-25 | 2.81E-24 |
| BC033323 /// CEP290 | 2.050624 | 2.24E-25 | 2.91E-24 |
| SMCHD1 | 2.061537 | 2.30E-25 | 2.98E-24 |
| LMNB1 | 4.955862 | 2.37E-25 | 3.06E-24 |
| NME1 | 2.799995 | 2.37E-25 | 3.06E-24 |
| DAP | 2.048172 | 2.42E-25 | 3.12E-24 |
| GTF2H2B | 3.349994 | 2.53E-25 | 3.25E-24 |
| KIF14 | 4.849434 | 2.71E-25 | 3.46E-24 |
| MELK | 3.857068 | 2.87E-25 | 3.66E-24 |
| CCDC34 | 2.252211 | 2.91E-25 | 3.72E-24 |
| UBE2C | 4.421828 | 3.07E-25 | 3.90E-24 |
| UBXN7 | 2.368596 | 3.16E-25 | 4.01E-24 |
| IL17RD | 4.148346 | 3.19E-25 | 4.05E-24 |
| TMEM243 | 2.566187 | 3.30E-25 | 4.19E-24 |
| TAF1A | 3.065891 | 3.35E-25 | 4.24E-24 |
| RBM12 | 2.985671 | 3.38E-25 | 4.28E-24 |
| PLIN4 | -3.10696 | 3.59E-25 | 4.52E-24 |
| MSANTD2 | 2.910019 | 3.71E-25 | 4.66E-24 |
| AGPAT4 | 2.378931 | 3.72E-25 | 4.67E-24 |
| ZNF84 | 2.649402 | 4.23E-25 | 5.28E-24 |
| COL1A1 | 2.977205 | 4.29E-25 | 5.35E-24 |
| H2BFXP | 2.269595 | 4.43E-25 | 5.51E-24 |
| LRP11 | 3.538227 | 4.54E-25 | 5.64E-24 |
| AGAP4 | 2.537623 | 4.73E-25 | 5.88E-24 |
| PTP4A3 | -2.44158 | 4.75E-25 | 5.89E-24 |
| FAM46C | -3.05043 | 4.92E-25 | 6.10E-24 |
| C3orf14 | 2.306923 | 5.46E-25 | 6.75E-24 |
| GNAI3 | 2.042689 | 5.51E-25 | 6.80E-24 |
| RBM12B | 2.945401 | 5.78E-25 | 7.12E-24 |
| DNAJB5 | -2.63174 | 6.09E-25 | 7.48E-24 |
| PGF | 2.368748 | 6.10E-25 | 7.48E-24 |
| LEPREL4 | 2.443687 | 6.25E-25 | 7.66E-24 |
| GPM6B | 4.144934 | 6.41E-25 | 7.85E-24 |
| MAD2L1 | 3.99409 | 7.23E-25 | 8.81E-24 |
| TUBB6 | 3.480929 | 7.28E-25 | 8.87E-24 |
| GMPR | -3.71499 | 7.53E-25 | 9.16E-24 |
| KLHL31 | -3.1428 | 7.58E-25 | 9.21E-24 |
| PPAT | 2.523805 | 7.59E-25 | 9.21E-24 |
| ZNF268 | 2.111134 | 7.77E-25 | 9.42E-24 |
| TOP1 | 2.882885 | 8.03E-25 | 9.71E-24 |
| SERP1 | 2.69457 | 8.12E-25 | 9.81E-24 |
| SMARCA5 | 2.179701 | 8.42E-25 | 1.02E-23 |
| NFE2L1 | -2.26406 | 8.60E-25 | 1.04E-23 |
| CALML6 | -3.62811 | 9.11E-25 | 1.10E-23 |
| ZNF431 | 2.782814 | 9.84E-25 | 1.18E-23 |
| ZNF700 | 2.825051 | 9.98E-25 | 1.19E-23 |
| PAXIP1 | 2.797229 | 1.01E-24 | 1.21E-23 |
| LEMD3 | 2.049296 | 1.06E-24 | 1.27E-23 |
| PPAPDC1B | 3.009886 | 1.07E-24 | 1.27E-23 |
| DCAF16 | 2.276115 | 1.09E-24 | 1.29E-23 |
| GPR137B | 3.440702 | 1.09E-24 | 1.30E-23 |
| TWSG1 | 2.730557 | 1.10E-24 | 1.31E-23 |
| DYNLL1 | 2.264415 | 1.17E-24 | 1.38E-23 |
| BTAF1 | 2.540107 | 1.25E-24 | 1.48E-23 |
| C1orf112 | 3.170023 | 1.28E-24 | 1.51E-23 |
| LOC102724356 | 3.685483 | 1.32E-24 | 1.56E-23 |
| ZNF850 | 3.794858 | 1.34E-24 | 1.57E-23 |
| RFC3 | 3.260346 | 1.34E-24 | 1.58E-23 |
| EHBP1 | 2.108212 | 1.36E-24 | 1.60E-23 |
| B3GALNT1 | 3.047943 | 1.36E-24 | 1.60E-23 |
| IMPACT | 2.316249 | 1.42E-24 | 1.66E-23 |
| RUSC1-AS1 | 2.042083 | 1.43E-24 | 1.68E-23 |
| KIF4A | 3.915569 | 1.45E-24 | 1.70E-23 |
| ARCN1 | 2.118983 | 1.54E-24 | 1.80E-23 |
| GMFB | 2.461877 | 1.55E-24 | 1.80E-23 |
| PTPRD | 3.940545 | 1.55E-24 | 1.81E-23 |
| TMPO | 2.505751 | 1.61E-24 | 1.88E-23 |
| BCL11B | 2.879507 | 1.72E-24 | 2.00E-23 |
| PAPD4 | 2.002091 | 1.76E-24 | 2.04E-23 |
| TNFRSF10B | 2.298635 | 1.80E-24 | 2.08E-23 |
| KRT31 | -2.67772 | 1.82E-24 | 2.11E-23 |
| FANCL | 3.205476 | 1.85E-24 | 2.14E-23 |
| TRO | 2.344736 | 1.94E-24 | 2.23E-23 |
| UHRF1 | 4.547911 | 1.96E-24 | 2.25E-23 |
| CYB5R1 | -2.12549 | 1.99E-24 | 2.29E-23 |
| HSD17B11 | 2.932758 | 2.05E-24 | 2.35E-23 |
| CRY1 | 2.419356 | 2.16E-24 | 2.47E-23 |
| ZNF426 | 2.242982 | 2.35E-24 | 2.67E-23 |
| C15orf61 | -2.33131 | 2.44E-24 | 2.77E-23 |
| TGIF1 | 2.142152 | 2.57E-24 | 2.91E-23 |
| NRAS | 2.179829 | 2.61E-24 | 2.95E-23 |
| ZFYVE16 | 2.027585 | 2.80E-24 | 3.16E-23 |
| FAM83D | 4.639971 | 2.80E-24 | 3.16E-23 |
| IL17D | -4.52001 | 2.81E-24 | 3.17E-23 |
| FBXL2 | 2.836117 | 2.83E-24 | 3.19E-23 |
| C5 | 2.444245 | 2.85E-24 | 3.21E-23 |
| ZNF267 | 2.235181 | 2.92E-24 | 3.28E-23 |
| CHD9 | 2.270765 | 2.96E-24 | 3.33E-23 |
| DNAJA1 | 2.019117 | 3.01E-24 | 3.37E-23 |
| ZC2HC1A | 2.184085 | 3.01E-24 | 3.37E-23 |
| GPD1L | -3.20125 | 3.04E-24 | 3.41E-23 |
| ZNF623 | 3.260101 | 3.05E-24 | 3.41E-23 |
| PRPF38B | 2.401947 | 3.15E-24 | 3.52E-23 |
| EP400 | 2.29352 | 3.16E-24 | 3.53E-23 |
| NINL | 3.245113 | 3.16E-24 | 3.53E-23 |
| DEPDC1 | 3.687022 | 3.16E-24 | 3.53E-23 |
| TXLNB | -5.53859 | 3.33E-24 | 3.71E-23 |
| ANKRA2 | 2.028879 | 3.51E-24 | 3.90E-23 |
| BRI3BP | 3.495544 | 3.60E-24 | 3.99E-23 |
| DONSON | 2.228157 | 3.61E-24 | 4.00E-23 |
| TMEM263 | 2.48315 | 3.71E-24 | 4.10E-23 |
| POP1 | 2.244997 | 3.78E-24 | 4.18E-23 |
| CASC5 | 2.368752 | 3.79E-24 | 4.19E-23 |
| ZNF202 | 2.36017 | 4.00E-24 | 4.42E-23 |
| RPGR | 2.219177 | 4.37E-24 | 4.80E-23 |
| SOAT1 | 2.542281 | 4.52E-24 | 4.95E-23 |
| LINC01355 | 2.231592 | 4.63E-24 | 5.06E-23 |
| TIGD2 | 2.372539 | 4.71E-24 | 5.14E-23 |
| ZNF682 | 3.131875 | 4.81E-24 | 5.23E-23 |
| CORO1C | 2.779066 | 4.89E-24 | 5.32E-23 |
| DTD2 | 2.003734 | 5.12E-24 | 5.54E-23 |
| TMEM194A | 3.136232 | 5.44E-24 | 5.87E-23 |
| PRRC2C | 2.13022 | 5.74E-24 | 6.17E-23 |
| SRBD1 | 2.054868 | 5.81E-24 | 6.24E-23 |
| PPAPDC3 | -2.85201 | 5.87E-24 | 6.30E-23 |
| E2F3 | 2.49427 | 5.96E-24 | 6.39E-23 |
| TSPAN5 | 3.142192 | 6.15E-24 | 6.58E-23 |
| CCNA2 | 3.594005 | 6.22E-24 | 6.64E-23 |
| SRGAP1 | 2.279657 | 6.29E-24 | 6.70E-23 |
| HILPDA | 2.421249 | 6.57E-24 | 6.98E-23 |
| MYD88 | 2.393937 | 6.62E-24 | 7.03E-23 |
| CLCN5 | 4.301795 | 6.82E-24 | 7.22E-23 |
| HMMR | 3.97473 | 6.88E-24 | 7.29E-23 |
| TSPYL4 | 2.514085 | 7.09E-24 | 7.49E-23 |
| PSRC1 | 3.866834 | 7.29E-24 | 7.69E-23 |
| MCM5 | 2.334354 | 7.43E-24 | 7.83E-23 |
| ANKHD1 | 3.292129 | 7.43E-24 | 7.83E-23 |
| N4BP3 | 2.353852 | 8.17E-24 | 8.57E-23 |
| COL18A1 | 2.90352 | 8.36E-24 | 8.76E-23 |
| RAD54B | 3.180588 | 8.59E-24 | 8.99E-23 |
| PHKA1 | -3.52647 | 8.62E-24 | 9.01E-23 |
| ARL4C | 2.529835 | 9.30E-24 | 9.68E-23 |
| LOC102724275 | 2.769124 | 9.87E-24 | 1.02E-22 |
| TMEM68 | 2.418887 | 9.90E-24 | 1.03E-22 |
| GAS1 | 2.98167 | 1.05E-23 | 1.08E-22 |
| MYOM1 | -4.59628 | 1.11E-23 | 1.15E-22 |
| AJUBA | 3.248089 | 1.12E-23 | 1.15E-22 |
| BAG3 | -2.84143 | 1.13E-23 | 1.16E-22 |
| CCDC112 | 2.332279 | 1.13E-23 | 1.16E-22 |
| CLTCL1 | -2.36949 | 1.13E-23 | 1.17E-22 |
| MGME1 | 2.244167 | 1.19E-23 | 1.22E-22 |
| DCK | 2.541451 | 1.20E-23 | 1.23E-22 |
| CDC23 | 2.144863 | 1.20E-23 | 1.24E-22 |
| ZNF273 | 2.349864 | 1.30E-23 | 1.33E-22 |
| TRIM59 | 4.971771 | 1.32E-23 | 1.34E-22 |
| SOWAHC | 2.697709 | 1.38E-23 | 1.41E-22 |
| E2F7 | 2.635169 | 1.39E-23 | 1.41E-22 |
| BORA | 3.126128 | 1.39E-23 | 1.42E-22 |
| FHL1 | -5.59659 | 1.46E-23 | 1.48E-22 |
| KIF15 | 4.695468 | 1.54E-23 | 1.56E-22 |
| ZBED8 | 2.822472 | 1.62E-23 | 1.63E-22 |
| OLFML2B | 4.085184 | 1.63E-23 | 1.64E-22 |
| CCDC77 | 2.590123 | 1.70E-23 | 1.70E-22 |
| THAP9-AS1 | 3.034794 | 1.71E-23 | 1.71E-22 |
| ZNF678 | 2.409682 | 1.74E-23 | 1.74E-22 |
| CCDC8 | 2.670058 | 1.79E-23 | 1.79E-22 |
| FPGT | 2.193058 | 1.80E-23 | 1.80E-22 |
| LINGO1 | 2.87479 | 1.83E-23 | 1.83E-22 |
| ASB2 | -4.24623 | 1.89E-23 | 1.89E-22 |
| TBCK | 2.265059 | 1.90E-23 | 1.89E-22 |
| ZNF367 | 3.835353 | 1.91E-23 | 1.91E-22 |
| TIA1 | 2.33727 | 1.94E-23 | 1.94E-22 |
| ZNF585A | 2.05704 | 1.97E-23 | 1.96E-22 |
| SLC16A2 | 2.531383 | 1.98E-23 | 1.97E-22 |
| MMD | 2.125496 | 1.98E-23 | 1.97E-22 |
| PNPLA4 | -2.75718 | 2.01E-23 | 1.99E-22 |
| CKAP2L | 3.535793 | 2.02E-23 | 2.01E-22 |
| EPB41L4A-AS1 | 2.115316 | 2.18E-23 | 2.15E-22 |
| NUP35 | 2.739477 | 2.30E-23 | 2.27E-22 |
| FAM60A | 3.381096 | 2.35E-23 | 2.31E-22 |
| C10orf71 | -3.76267 | 2.35E-23 | 2.31E-22 |
| CKS1B | 2.820634 | 2.50E-23 | 2.46E-22 |
| LBR | 3.146156 | 2.63E-23 | 2.58E-22 |
| C4orf46 | 3.331659 | 2.80E-23 | 2.74E-22 |
| REV3L | 2.427656 | 2.84E-23 | 2.77E-22 |
| PI4KA | 2.344627 | 2.87E-23 | 2.80E-22 |
| TXN | 2.006669 | 2.88E-23 | 2.81E-22 |
| DNAJB6 | -2.57143 | 2.89E-23 | 2.82E-22 |
| SPATS2L | 3.169219 | 2.92E-23 | 2.85E-22 |
| VKORC1 | 2.236496 | 3.15E-23 | 3.06E-22 |
| E2F5 | 3.443874 | 3.27E-23 | 3.17E-22 |
| C6orf48 | 2.279675 | 3.31E-23 | 3.21E-22 |
| PAQR8 | 2.104016 | 3.36E-23 | 3.25E-22 |
| KMT2E | 2.044384 | 3.71E-23 | 3.57E-22 |
| BAZ1A | 3.012709 | 4.90E-23 | 4.66E-22 |
| COTL1 | 2.153358 | 5.10E-23 | 4.84E-22 |
| FLJ31306 | 2.352192 | 5.27E-23 | 4.99E-22 |
| ZNF512B | 2.34083 | 5.47E-23 | 5.18E-22 |
| PCDHB14 | 3.444517 | 6.23E-23 | 5.87E-22 |
| TIMP2 | 2.759527 | 6.24E-23 | 5.88E-22 |
| CACNB1 | -2.80935 | 6.93E-23 | 6.48E-22 |
| KIAA1731 | 2.333358 | 7.36E-23 | 6.87E-22 |
| ASPM | 4.529674 | 7.87E-23 | 7.30E-22 |
| VCAN | 4.429809 | 8.24E-23 | 7.62E-22 |
| CDH11 | 3.557316 | 8.27E-23 | 7.65E-22 |
| ZNF184 | 2.668964 | 8.30E-23 | 7.68E-22 |
| KLHL2 | 2.551565 | 8.47E-23 | 7.82E-22 |
| TMEM181 | 2.32136 | 8.65E-23 | 7.98E-22 |
| EFNA4 | 2.316403 | 8.94E-23 | 8.23E-22 |
| U2SURP | 2.206034 | 9.05E-23 | 8.33E-22 |
| MAPT | -2.16573 | 9.11E-23 | 8.38E-22 |
| SMC2 | 2.722431 | 9.57E-23 | 8.79E-22 |
| CEP83 | 2.061377 | 9.68E-23 | 8.89E-22 |
| VPS37B | 2.439605 | 9.70E-23 | 8.90E-22 |
| ATP13A3 | 2.063257 | 1.02E-22 | 9.32E-22 |
| PRMT6 | 2.673989 | 1.05E-22 | 9.63E-22 |
| CNOT6 | 2.013764 | 1.10E-22 | 1.00E-21 |
| TTC8 | 2.868208 | 1.12E-22 | 1.02E-21 |
| SLC39A10 | 3.22141 | 1.15E-22 | 1.05E-21 |
| ZNF432 | 2.84783 | 1.25E-22 | 1.14E-21 |
| ZEB1-AS1 | 2.62089 | 1.32E-22 | 1.19E-21 |
| SVIL | -2.06217 | 1.33E-22 | 1.20E-21 |
| CACNG1 | -3.49845 | 1.36E-22 | 1.23E-21 |
| RMI2 | 2.428601 | 1.37E-22 | 1.24E-21 |
| ZFP14 | 2.118768 | 1.38E-22 | 1.25E-21 |
| SQLE | 2.426517 | 1.40E-22 | 1.26E-21 |
| CHEK1 | 2.972713 | 1.42E-22 | 1.28E-21 |
| MCM3 | 2.112891 | 1.43E-22 | 1.29E-21 |
| NUPL1 | 2.134251 | 1.56E-22 | 1.40E-21 |
| TMEM38A | -3.69424 | 1.60E-22 | 1.43E-21 |
| THRB | -2.43847 | 1.63E-22 | 1.46E-21 |
| IFFO1 | 2.271633 | 1.67E-22 | 1.50E-21 |
| SLC2A10 | 3.072553 | 1.69E-22 | 1.51E-21 |
| ST3GAL4-AS1 | 2.172379 | 1.73E-22 | 1.54E-21 |
| ATP1A2 | -5.05592 | 1.75E-22 | 1.56E-21 |
| TRIP10 | -2.27295 | 1.77E-22 | 1.58E-21 |
| MIR133A1HG | -4.48101 | 1.80E-22 | 1.61E-21 |
| LRRC39 | -4.29181 | 1.87E-22 | 1.66E-21 |
| MCM10 | 3.467773 | 2.16E-22 | 1.91E-21 |
| AK1 | -2.78067 | 2.17E-22 | 1.92E-21 |
| SPP1 | 3.438106 | 2.19E-22 | 1.93E-21 |
| CIPC | -2.39052 | 2.27E-22 | 2.00E-21 |
| PACSIN3 | -2.63568 | 2.32E-22 | 2.03E-21 |
| CDCA3 | 3.066601 | 2.34E-22 | 2.05E-21 |
| CKMT2 | -5.85887 | 2.47E-22 | 2.16E-21 |
| MCM3AP-AS1 | 2.208454 | 2.53E-22 | 2.21E-21 |
| PLCL1 | -2.42598 | 2.68E-22 | 2.33E-21 |
| MYL2 | -8.28656 | 2.70E-22 | 2.35E-21 |
| SOX11 | 6.066012 | 2.79E-22 | 2.42E-21 |
| CAMK2B | -2.53558 | 2.80E-22 | 2.43E-21 |
| BAX | 2.287687 | 2.80E-22 | 2.43E-21 |
| CCNB2 | 2.556194 | 3.01E-22 | 2.60E-21 |
| FSCN1 | 2.668609 | 3.15E-22 | 2.72E-21 |
| PELI1 | 2.834523 | 3.23E-22 | 2.79E-21 |
| TMA16 | 2.619341 | 3.33E-22 | 2.87E-21 |
| ZNF519 | 2.456206 | 3.37E-22 | 2.90E-21 |
| GEN1 | 2.224165 | 3.51E-22 | 3.01E-21 |
| PPP1R3C | -3.44663 | 3.63E-22 | 3.10E-21 |
| JADE3 | 2.655479 | 3.67E-22 | 3.13E-21 |
| ZUFSP | 2.082284 | 3.71E-22 | 3.16E-21 |
| PKD2 | 2.427018 | 3.87E-22 | 3.29E-21 |
| TMOD1 | -3.62742 | 3.88E-22 | 3.30E-21 |
| GPATCH1 | 2.080516 | 4.00E-22 | 3.40E-21 |
| KCNQ5 | -2.65998 | 4.10E-22 | 3.48E-21 |
| IER2 | 3.145831 | 4.35E-22 | 3.68E-21 |
| LEPRE1 | 2.207175 | 4.51E-22 | 3.82E-21 |
| CDCA4 | 2.652115 | 4.64E-22 | 3.92E-21 |
| MAPKAPK3 | -2.39931 | 4.68E-22 | 3.95E-21 |
| HSPA13 | 2.836107 | 4.71E-22 | 3.98E-21 |
| CDK2AP1 | 2.329507 | 4.76E-22 | 4.02E-21 |
| GPR153 | 2.025938 | 4.91E-22 | 4.14E-21 |
| STRBP | 2.072643 | 4.95E-22 | 4.17E-21 |
| BCAT1 | 4.726027 | 4.99E-22 | 4.20E-21 |
| ZNF880 | 2.197739 | 5.05E-22 | 4.24E-21 |
| LRRC49 | 2.558644 | 5.05E-22 | 4.25E-21 |
| MPP7 | -2.09778 | 5.26E-22 | 4.41E-21 |
| DUSP13 | -3.40939 | 5.46E-22 | 4.58E-21 |
| ZNF117 | 2.085136 | 6.09E-22 | 5.08E-21 |
| VPS13B | 2.014664 | 6.36E-22 | 5.30E-21 |
| LOC100134822 | 2.502573 | 6.67E-22 | 5.54E-21 |
| ZSCAN16 | 2.219873 | 6.86E-22 | 5.69E-21 |
| EPC2 | 2.032601 | 7.12E-22 | 5.90E-21 |
| DEPDC1B | 4.40894 | 7.30E-22 | 6.04E-21 |
| CENPF | 3.626978 | 7.32E-22 | 6.05E-21 |
| SKA3 | 2.972075 | 7.33E-22 | 6.06E-21 |
| GTSE1 | 2.486436 | 7.40E-22 | 6.12E-21 |
| TM2D2 | 2.067708 | 7.43E-22 | 6.14E-21 |
| PARPBP | 2.163007 | 7.55E-22 | 6.23E-21 |
| MIF | 2.805134 | 7.62E-22 | 6.29E-21 |
| KIF2C | 3.215515 | 7.71E-22 | 6.35E-21 |
| BTBD18 | 2.081718 | 8.84E-22 | 7.24E-21 |
| FKBP7 | 2.242793 | 9.06E-22 | 7.42E-21 |
| CORO6 | -3.49655 | 1.00E-21 | 8.16E-21 |
| ADD3 | 2.347678 | 1.01E-21 | 8.27E-21 |
| MORC4 | 3.038424 | 1.10E-21 | 8.90E-21 |
| DPY19L4 | 2.459553 | 1.20E-21 | 9.64E-21 |
| KIF18A | 3.869425 | 1.22E-21 | 9.81E-21 |
| SYPL2 | -3.86559 | 1.24E-21 | 9.95E-21 |
| DLGAP5 | 4.53468 | 1.24E-21 | 9.97E-21 |
| MFSD1 | 2.55632 | 1.25E-21 | 1.00E-20 |
| ESPL1 | 2.763199 | 1.27E-21 | 1.02E-20 |
| FAM107B | 2.647712 | 1.31E-21 | 1.05E-20 |
| GLCE | 2.719117 | 1.32E-21 | 1.06E-20 |
| SCRN1 | 3.323515 | 1.49E-21 | 1.19E-20 |
| AQP4 | -3.6363 | 1.66E-21 | 1.32E-20 |
| FKBP1B | 3.622005 | 1.67E-21 | 1.33E-20 |
| HS6ST2 | 4.215976 | 1.87E-21 | 1.48E-20 |
| HMGCR | 2.081325 | 1.98E-21 | 1.56E-20 |
| LOC100506119 | 2.132939 | 2.01E-21 | 1.58E-20 |
| SNORD114-3 | 5.89888 | 2.01E-21 | 1.59E-20 |
| LOC100506473 /// RP11-332H14.2 | 2.004214 | 2.06E-21 | 1.62E-20 |
| CTHRC1 | 4.510822 | 2.11E-21 | 1.66E-20 |
| SERTAD2 | 2.457025 | 2.15E-21 | 1.69E-20 |
| MIR100HG | 3.651438 | 2.15E-21 | 1.69E-20 |
| PSTPIP2 | -2.52351 | 2.24E-21 | 1.75E-20 |
| MYO1B | 2.23056 | 2.27E-21 | 1.78E-20 |
| NPC1 | 2.077173 | 2.38E-21 | 1.86E-20 |
| ST6GALNAC2 | -3.05345 | 2.51E-21 | 1.96E-20 |
| MYH6 | -2.93078 | 3.00E-21 | 2.32E-20 |
| BIN1 | -2.81705 | 3.01E-21 | 2.33E-20 |
| CENPJ | 2.387373 | 3.12E-21 | 2.41E-20 |
| HLTF | 2.121423 | 3.20E-21 | 2.46E-20 |
| HAUS6 | 2.523579 | 3.25E-21 | 2.50E-20 |
| ZFP62 | 2.227964 | 3.25E-21 | 2.50E-20 |
| GNAL | -2.81953 | 3.41E-21 | 2.63E-20 |
| ADAM12 | 3.333755 | 3.60E-21 | 2.77E-20 |
| PLEKHA5 | 2.056678 | 3.73E-21 | 2.86E-20 |
| SEC24A | 2.012573 | 3.82E-21 | 2.93E-20 |
| NSUN6 | 2.827117 | 3.84E-21 | 2.94E-20 |
| NCAPD2 | 2.674608 | 3.89E-21 | 2.98E-20 |
| LOC101929787 | 2.850321 | 3.97E-21 | 3.03E-20 |
| PLAGL1 | 3.993656 | 4.06E-21 | 3.10E-20 |
| TRMT13 | 2.360962 | 4.14E-21 | 3.16E-20 |
| UBE2S | 3.071725 | 4.18E-21 | 3.18E-20 |
| DNAJB14 | 2.221928 | 4.18E-21 | 3.19E-20 |
| TUSC3 | 2.805992 | 4.22E-21 | 3.21E-20 |
| ZBTB34 | 2.000929 | 4.45E-21 | 3.38E-20 |
| TIMP4 | -2.52215 | 4.52E-21 | 3.43E-20 |
| NAP1L3 | 3.616852 | 4.56E-21 | 3.46E-20 |
| PRPF3 | 2.204357 | 5.00E-21 | 3.78E-20 |
| LOC100272216 | 2.879215 | 5.20E-21 | 3.92E-20 |
| TMEM14A | 2.376904 | 5.20E-21 | 3.92E-20 |
| EXOSC8 | 2.135202 | 5.31E-21 | 3.99E-20 |
| SLC26A9 | -2.06871 | 5.41E-21 | 4.06E-20 |
| GYS1 | -2.41215 | 5.46E-21 | 4.09E-20 |
| AEBP1 | 3.089665 | 5.75E-21 | 4.31E-20 |
| GXYLT2 | 3.878443 | 5.78E-21 | 4.33E-20 |
| AURKA | 2.205634 | 5.94E-21 | 4.45E-20 |
| TACC3 | 2.425022 | 6.06E-21 | 4.53E-20 |
| TPST1 | 2.116984 | 6.20E-21 | 4.63E-20 |
| MYOM2 | -4.20885 | 6.27E-21 | 4.68E-20 |
| RGS1 | 3.325828 | 6.28E-21 | 4.68E-20 |
| ADRBK2 | 2.267252 | 6.45E-21 | 4.80E-20 |
| TMEM260 | 2.956845 | 7.06E-21 | 5.25E-20 |
| FAM114A1 | 2.135357 | 7.18E-21 | 5.33E-20 |
| C17orf62 | 2.045189 | 7.29E-21 | 5.40E-20 |
| FN1 | 2.36206 | 7.60E-21 | 5.62E-20 |
| ANKRD23 | -2.26352 | 8.51E-21 | 6.26E-20 |
| SHD | 4.29507 | 8.73E-21 | 6.42E-20 |
| NMRK2 | -3.45978 | 9.22E-21 | 6.76E-20 |
| DDB2 | 2.115506 | 1.01E-20 | 7.36E-20 |
| EIF4E3 | -2.34239 | 1.01E-20 | 7.41E-20 |
| HSPA2 | -4.39231 | 1.13E-20 | 8.23E-20 |
| RNF138 | 2.068522 | 1.14E-20 | 8.27E-20 |
| LRIG3 | 4.501772 | 1.14E-20 | 8.29E-20 |
| BLM | 3.005836 | 1.25E-20 | 9.07E-20 |
| WIPF3 | -2.29168 | 1.32E-20 | 9.53E-20 |
| NUSAP1 | 3.77097 | 1.34E-20 | 9.67E-20 |
| LONRF2 | -2.93964 | 1.34E-20 | 9.69E-20 |
| LDB3 | -3.16941 | 1.40E-20 | 1.01E-19 |
| CEP128 | 2.036753 | 1.49E-20 | 1.07E-19 |
| MKI67 | 3.189852 | 1.50E-20 | 1.08E-19 |
| PNMA1 | 2.495764 | 1.51E-20 | 1.08E-19 |
| FCGBP | 2.976685 | 1.57E-20 | 1.12E-19 |
| IFIT5 | 2.217609 | 1.59E-20 | 1.14E-19 |
| DOCK10 | 2.80431 | 1.62E-20 | 1.16E-19 |
| RNASEH2A | 2.569008 | 1.64E-20 | 1.17E-19 |
| FAM64A | 3.409552 | 1.66E-20 | 1.18E-19 |
| CWC22 | 2.17272 | 1.84E-20 | 1.31E-19 |
| PPWD1 | 2.038262 | 1.86E-20 | 1.32E-19 |
| LOC102606465 | 2.612119 | 1.90E-20 | 1.35E-19 |
| TPM3 | -2.17082 | 1.94E-20 | 1.37E-19 |
| SRGAP2C | 2.096712 | 2.01E-20 | 1.42E-19 |
| ILF3 | 2.022284 | 2.04E-20 | 1.44E-19 |
| RAPH1 | 2.910655 | 2.17E-20 | 1.53E-19 |
| SMARCC1 | 2.169274 | 2.19E-20 | 1.54E-19 |
| FAM24B | 2.478102 | 2.31E-20 | 1.62E-19 |
| BRIP1 | 3.397755 | 2.31E-20 | 1.62E-19 |
| GJC1 | 2.020913 | 2.36E-20 | 1.66E-19 |
| DDIAS | 2.875944 | 2.39E-20 | 1.68E-19 |
| SLC16A4 | 2.293838 | 2.43E-20 | 1.71E-19 |
| PMS2P5 | 2.338962 | 2.57E-20 | 1.80E-19 |
| ATP2B1 | 2.628851 | 2.76E-20 | 1.92E-19 |
| KIAA1211 | 2.304563 | 2.79E-20 | 1.95E-19 |
| NUDCD1 | 2.341322 | 2.83E-20 | 1.97E-19 |
| SERPINH1 | 2.992742 | 2.93E-20 | 2.04E-19 |
| ZNF567 | 2.28676 | 3.13E-20 | 2.17E-19 |
| NOTCH2 | 2.171627 | 3.19E-20 | 2.21E-19 |
| TCF7L2 | 2.609993 | 3.51E-20 | 2.42E-19 |
| HHAT | 2.315326 | 3.55E-20 | 2.44E-19 |
| FKBP14 | 2.068467 | 3.56E-20 | 2.45E-19 |
| ZFAND2A | 2.07817 | 4.17E-20 | 2.85E-19 |
| SESTD1 | 2.296968 | 4.75E-20 | 3.24E-19 |
| MYOM3 | -2.82614 | 5.15E-20 | 3.49E-19 |
| LOC644656 | 2.111119 | 5.15E-20 | 3.49E-19 |
| LOC202181 | 3.331588 | 5.24E-20 | 3.55E-19 |
| SMC3 | 2.530794 | 5.32E-20 | 3.61E-19 |
| PEG3 | 6.763382 | 5.39E-20 | 3.65E-19 |
| SLC2A5 | -2.16878 | 5.43E-20 | 3.67E-19 |
| ELFN1 | 2.547512 | 5.70E-20 | 3.84E-19 |
| CDC6 | 2.849103 | 5.86E-20 | 3.95E-19 |
| ST5 | 2.393614 | 6.22E-20 | 4.18E-19 |
| AACS | 2.250549 | 6.24E-20 | 4.19E-19 |
| ATP1B1 | -3.08887 | 6.27E-20 | 4.21E-19 |
| GMNN | 2.358904 | 6.28E-20 | 4.21E-19 |
| ZNF713 | 2.10973 | 6.44E-20 | 4.31E-19 |
| GABPB1-AS1 | 2.533155 | 6.78E-20 | 4.53E-19 |
| SDF2L1 | 2.345687 | 6.91E-20 | 4.61E-19 |
| CCND2 | 2.658876 | 7.59E-20 | 5.04E-19 |
| TCF4 | 2.436708 | 7.82E-20 | 5.18E-19 |
| KIFC1 | 2.353046 | 7.90E-20 | 5.23E-19 |
| WDHD1 | 2.303977 | 8.62E-20 | 5.68E-19 |
| MB | -7.10118 | 1.02E-19 | 6.66E-19 |
| LOC730101 | 2.348208 | 1.09E-19 | 7.08E-19 |
| EGR2 | 3.71006 | 1.14E-19 | 7.37E-19 |
| RTN2 | -2.35568 | 1.17E-19 | 7.58E-19 |
| MCM2 | 2.453528 | 1.27E-19 | 8.21E-19 |
| ZNF227 | 2.240959 | 1.28E-19 | 8.26E-19 |
| POSTN | 3.679891 | 1.45E-19 | 9.30E-19 |
| EFHD2 | 2.154354 | 1.51E-19 | 9.71E-19 |
| ABAT | 4.701924 | 1.53E-19 | 9.83E-19 |
| SKP2 | 3.654637 | 1.54E-19 | 9.87E-19 |
| DDHD2 | 2.402693 | 1.55E-19 | 9.94E-19 |
| NAV1 | 2.256105 | 1.57E-19 | 1.01E-18 |
| NUDT11 | 4.402737 | 1.65E-19 | 1.06E-18 |
| ZNF667-AS1 | 2.056463 | 1.71E-19 | 1.09E-18 |
| BIRC5 /// EPR-1 | 2.625073 | 1.84E-19 | 1.17E-18 |
| COX7A1 | -4.39993 | 1.85E-19 | 1.17E-18 |
| FZD2 | 2.755139 | 1.88E-19 | 1.19E-18 |
| HRC | -3.64336 | 1.96E-19 | 1.24E-18 |
| JADE1 | 2.09351 | 1.98E-19 | 1.26E-18 |
| SLCO5A1 | -3.01904 | 2.14E-19 | 1.35E-18 |
| DLEU2 | 2.407961 | 2.18E-19 | 1.38E-18 |
| ROBO1 | 3.526478 | 2.29E-19 | 1.44E-18 |
| RPL23AP32 | 2.251729 | 2.38E-19 | 1.49E-18 |
| KCTD3 | 2.56281 | 2.38E-19 | 1.49E-18 |
| DDN | -2.09743 | 2.55E-19 | 1.59E-18 |
| DDX26B | 3.023695 | 2.59E-19 | 1.61E-18 |
| COL16A1 | 2.965394 | 2.63E-19 | 1.64E-18 |
| SNHG19 | 2.191853 | 2.92E-19 | 1.81E-18 |
| CCDC91 | 2.067415 | 2.93E-19 | 1.82E-18 |
| LOC102724718 | 2.030418 | 3.21E-19 | 1.99E-18 |
| KIAA1524 | 2.726487 | 3.32E-19 | 2.05E-18 |
| LUC7L3 | 2.210412 | 3.40E-19 | 2.09E-18 |
| TNFAIP8 | 2.408183 | 3.56E-19 | 2.19E-18 |
| SCG5 | 3.574508 | 3.86E-19 | 2.36E-18 |
| SALL2 | 2.0248 | 3.90E-19 | 2.38E-18 |
| MEGF10 | 3.554074 | 3.92E-19 | 2.40E-18 |
| VASH2 | 3.742889 | 3.99E-19 | 2.44E-18 |
| KIAA1551 | 2.248378 | 4.60E-19 | 2.80E-18 |
| NUDT15 | 2.359528 | 4.70E-19 | 2.86E-18 |
| FYN | 2.764169 | 4.96E-19 | 3.01E-18 |
| MYOT | -7.80421 | 4.96E-19 | 3.01E-18 |
| SSX2IP | 2.10482 | 5.01E-19 | 3.04E-18 |
| ZNF22 | 2.126689 | 5.16E-19 | 3.12E-18 |
| HS3ST3B1 | 2.360925 | 5.19E-19 | 3.14E-18 |
| ZNF790-AS1 | 2.437618 | 5.71E-19 | 3.44E-18 |
| TRMT11 | 2.613263 | 5.81E-19 | 3.50E-18 |
| MYBPC2 | -6.10845 | 6.07E-19 | 3.65E-18 |
| RBM4 | 2.025743 | 6.11E-19 | 3.67E-18 |
| APMAP | 2.229717 | 6.16E-19 | 3.69E-18 |
| ACTN2 | -4.49769 | 6.31E-19 | 3.78E-18 |
| KIAA1549 | 3.13369 | 6.76E-19 | 4.04E-18 |
| KBTBD6 | 2.691664 | 7.14E-19 | 4.26E-18 |
| LOC283357 | 2.392913 | 7.72E-19 | 4.59E-18 |
| SOX8 | 5.834384 | 7.92E-19 | 4.70E-18 |
| CCDC150 | 2.199434 | 8.10E-19 | 4.80E-18 |
| UBXN8 | 2.23992 | 8.19E-19 | 4.85E-18 |
| BAZ2B | 2.390069 | 8.49E-19 | 5.02E-18 |
| GPX8 | 3.713599 | 8.54E-19 | 5.04E-18 |
| TIMP1 | 3.601665 | 9.16E-19 | 5.39E-18 |
| RAB31 | 2.847721 | 9.98E-19 | 5.86E-18 |
| VAX2 | 2.143218 | 1.03E-18 | 6.05E-18 |
| TMEM45A | 4.000741 | 1.13E-18 | 6.59E-18 |
| MDK | 3.068393 | 1.18E-18 | 6.88E-18 |
| MCAM | 2.231388 | 1.18E-18 | 6.89E-18 |
| SRGAP2 | 2.123232 | 1.19E-18 | 6.94E-18 |
| GPALPP1 | 2.134337 | 1.24E-18 | 7.23E-18 |
| MEG9 | 3.50332 | 1.29E-18 | 7.47E-18 |
| EFS | 3.100276 | 1.34E-18 | 7.79E-18 |
| GLIDR | 2.340218 | 1.39E-18 | 8.08E-18 |
| DNAJA4 | -2.4226 | 1.40E-18 | 8.12E-18 |
| EXT1 | 2.376654 | 1.42E-18 | 8.23E-18 |
| MSH2 | 2.524021 | 1.46E-18 | 8.42E-18 |
| PLEKHA2 | 2.602113 | 1.47E-18 | 8.50E-18 |
| KIZ | 2.137372 | 1.53E-18 | 8.85E-18 |
| TMEM182 | -3.90056 | 1.55E-18 | 8.91E-18 |
| TAF5 | 2.305801 | 1.71E-18 | 9.79E-18 |
| DDIT4L | -4.33942 | 1.75E-18 | 1.00E-17 |
| FAM189A2 | -2.85984 | 1.77E-18 | 1.02E-17 |
| PNISR | 2.053837 | 1.90E-18 | 1.09E-17 |
| MYO10 | 2.382935 | 1.94E-18 | 1.11E-17 |
| PDE9A | 2.045596 | 2.14E-18 | 1.22E-17 |
| SPC25 | 3.684025 | 2.15E-18 | 1.23E-17 |
| LOC81691 | 2.068458 | 2.21E-18 | 1.26E-17 |
| TNNT3 | -4.58562 | 2.33E-18 | 1.32E-17 |
| LOC494150 | 2.070266 | 2.34E-18 | 1.32E-17 |
| MPHOSPH9 | 2.180248 | 2.39E-18 | 1.35E-17 |
| OCIAD2 | 2.387749 | 2.40E-18 | 1.36E-17 |
| SMTNL2 | -3.43359 | 2.42E-18 | 1.37E-17 |
| PHLDB2 | 2.20932 | 2.47E-18 | 1.40E-17 |
| TTI1 | 2.175494 | 2.47E-18 | 1.40E-17 |
| PTPRF | 2.124487 | 2.63E-18 | 1.48E-17 |
| XIRP2 | -7.00934 | 2.70E-18 | 1.52E-17 |
| COL4A1 | 2.581389 | 2.74E-18 | 1.54E-17 |
| MAOA | -2.9312 | 2.83E-18 | 1.59E-17 |
| TUBB2A | 2.810286 | 2.89E-18 | 1.62E-17 |
| HAUS3 | 2.103312 | 2.90E-18 | 1.62E-17 |
| NEIL3 | 2.264105 | 3.18E-18 | 1.77E-17 |
| LRRC8D | 2.145872 | 3.27E-18 | 1.82E-17 |
| DUSP26 | -2.60987 | 3.34E-18 | 1.86E-17 |
| GXYLT1 | 2.084457 | 3.68E-18 | 2.04E-17 |
| PCDHB16 | 3.984054 | 4.08E-18 | 2.25E-17 |
| MIR3682 | 2.365977 | 4.14E-18 | 2.28E-17 |
| HINT3 | -2.40373 | 4.46E-18 | 2.45E-17 |
| SDC2 | 2.730765 | 4.66E-18 | 2.55E-17 |
| NET1 | 2.85145 | 4.67E-18 | 2.55E-17 |
| SNHG17 | 2.229503 | 4.86E-18 | 2.65E-17 |
| GALNT16 | 2.049564 | 5.00E-18 | 2.73E-17 |
| CDK4 | 2.306559 | 5.13E-18 | 2.79E-17 |
| SNAPC1 | 2.102617 | 5.42E-18 | 2.95E-17 |
| LINC00597 | 2.662111 | 5.61E-18 | 3.04E-17 |
| STC2 | 2.987591 | 6.26E-18 | 3.38E-17 |
| ZNF711 | 3.900516 | 6.40E-18 | 3.45E-17 |
| CEP72 | 2.187272 | 7.05E-18 | 3.79E-17 |
| CDCA2 | 2.849116 | 7.10E-18 | 3.82E-17 |
| THY1 | 2.563356 | 8.17E-18 | 4.37E-17 |
| BCL11A | 4.342522 | 8.65E-18 | 4.61E-17 |
| ZBTB21 | 2.305902 | 9.13E-18 | 4.86E-17 |
| RGS2 | 3.335453 | 9.13E-18 | 4.86E-17 |
| LOC100507165 | 2.054801 | 9.54E-18 | 5.07E-17 |
| GPX7 | 2.375269 | 9.57E-18 | 5.09E-17 |
| PAN2 | 2.090941 | 9.89E-18 | 5.25E-17 |
| ENO3 | -2.85369 | 1.08E-17 | 5.73E-17 |
| CYP2J2 | -3.14036 | 1.12E-17 | 5.92E-17 |
| PROSER1 | 2.216433 | 1.17E-17 | 6.18E-17 |
| DAPK1-IT1 /// DAPK1-IT1 | 2.85994 | 1.18E-17 | 6.19E-17 |
| ZNF140 | 2.557283 | 1.20E-17 | 6.30E-17 |
| DTX4 | 2.721609 | 1.29E-17 | 6.77E-17 |
| ARHGEF26 | 2.145976 | 1.40E-17 | 7.31E-17 |
| SEMA6A | 2.65438 | 1.44E-17 | 7.53E-17 |
| CYB5R2 | 2.366192 | 1.51E-17 | 7.87E-17 |
| FAM178A | 2.508338 | 1.58E-17 | 8.24E-17 |
| PHOSPHO2 | 2.218464 | 1.63E-17 | 8.48E-17 |
| ZNF57 | 3.655734 | 1.66E-17 | 8.59E-17 |
| ITGA4 | 2.400896 | 1.94E-17 | 9.97E-17 |
| EFNA5 | 2.211494 | 1.94E-17 | 9.98E-17 |
| AGPAT5 | 2.731259 | 1.94E-17 | 9.99E-17 |
| SMIM17 | 2.414983 | 1.98E-17 | 1.02E-16 |
| KLHL40 | -3.78462 | 2.33E-17 | 1.19E-16 |
| PSD3 | 3.340607 | 2.36E-17 | 1.21E-16 |
| ARHGEF3 | 2.082489 | 2.39E-17 | 1.22E-16 |
| EFCAB7 | 2.277892 | 2.40E-17 | 1.22E-16 |
| PEG3-AS1 | 4.015884 | 2.63E-17 | 1.34E-16 |
| TNNI2 | -5.37078 | 2.71E-17 | 1.37E-16 |
| SGCG | -4.65202 | 2.81E-17 | 1.42E-16 |
| EBPL | 2.13336 | 2.85E-17 | 1.44E-16 |
| NAIP | 2.378181 | 2.89E-17 | 1.46E-16 |
| PCK2 | 2.016758 | 3.03E-17 | 1.53E-16 |
| SYNDIG1 | 2.826238 | 3.18E-17 | 1.60E-16 |
| TBC1D32 | 2.788684 | 3.20E-17 | 1.61E-16 |
| ZNF600 | 2.180271 | 3.24E-17 | 1.63E-16 |
| PCDHB6 | 2.078782 | 3.24E-17 | 1.63E-16 |
| BGN | 2.600248 | 3.32E-17 | 1.67E-16 |
| OXTR | 3.102019 | 3.48E-17 | 1.74E-16 |
| ENC1 | 2.917588 | 3.58E-17 | 1.79E-16 |
| LOC101929726 | 4.252846 | 3.59E-17 | 1.79E-16 |
| HJURP | 3.044045 | 3.69E-17 | 1.84E-16 |
| CMTR2 | 2.537108 | 3.74E-17 | 1.87E-16 |
| KCNH2 | 2.551256 | 3.84E-17 | 1.91E-16 |
| GEM | 3.377972 | 3.90E-17 | 1.94E-16 |
| SLC20A2 | -2.17288 | 4.07E-17 | 2.02E-16 |
| STRIP2 | -2.23063 | 4.25E-17 | 2.11E-16 |
| NUP107 | 2.647774 | 4.62E-17 | 2.28E-16 |
| CDKN3 | 2.973813 | 4.65E-17 | 2.30E-16 |
| CIART | -2.11234 | 4.69E-17 | 2.32E-16 |
| HBB | -4.28851 | 4.80E-17 | 2.37E-16 |
| CDK6 | 2.724097 | 5.01E-17 | 2.47E-16 |
| ZNF112 | 2.348997 | 5.30E-17 | 2.61E-16 |
| MND1 | 3.55159 | 5.38E-17 | 2.64E-16 |
| IRAK1BP1 | 2.509602 | 5.49E-17 | 2.70E-16 |
| DLGAP1-AS1 | 2.282734 | 5.51E-17 | 2.71E-16 |
| MLLT3 | 2.573426 | 6.01E-17 | 2.94E-16 |
| CFL2 | -2.58445 | 6.69E-17 | 3.26E-16 |
| G0S2 | -3.52232 | 6.87E-17 | 3.34E-16 |
| NEXN | -3.10377 | 7.37E-17 | 3.58E-16 |
| SH3BGRL | 2.176357 | 7.50E-17 | 3.64E-16 |
| DHFR | 2.696066 | 8.27E-17 | 4.00E-16 |
| ZNF883 | 2.787867 | 8.49E-17 | 4.10E-16 |
| PLA2G7 | 2.912258 | 8.90E-17 | 4.29E-16 |
| ANXA3 | -2.6307 | 9.02E-17 | 4.34E-16 |
| CKM | -6.91723 | 9.12E-17 | 4.39E-16 |
| SMAP2 | 2.040247 | 9.52E-17 | 4.58E-16 |
| MEX3B | 2.125618 | 1.00E-16 | 4.82E-16 |
| CSRP2 | 3.276165 | 1.02E-16 | 4.88E-16 |
| CCDC102B | 3.682816 | 1.03E-16 | 4.96E-16 |
| JPX | 3.10926 | 1.04E-16 | 5.00E-16 |
| PRKY | 2.096703 | 1.07E-16 | 5.12E-16 |
| HNRNPU-AS1 | 2.778046 | 1.10E-16 | 5.26E-16 |
| DFFB | 2.196479 | 1.16E-16 | 5.53E-16 |
| PPP1R16A | -2.27589 | 1.18E-16 | 5.61E-16 |
| TTC39C | 2.19568 | 1.36E-16 | 6.45E-16 |
| ACSL1 | -2.98047 | 1.48E-16 | 7.01E-16 |
| MEIS2 | 3.929981 | 1.55E-16 | 7.29E-16 |
| FKBP5 | -2.86339 | 1.58E-16 | 7.42E-16 |
| MGC24103 | 3.317527 | 1.58E-16 | 7.45E-16 |
| AGL | -2.83266 | 1.59E-16 | 7.48E-16 |
| RBP1 | 2.120294 | 1.66E-16 | 7.78E-16 |
| INTU | 3.068211 | 1.75E-16 | 8.17E-16 |
| LHFPL2 | 2.190356 | 1.84E-16 | 8.59E-16 |
| SHPRH | 2.216509 | 1.92E-16 | 8.93E-16 |
| HSPB8 | -3.10758 | 1.95E-16 | 9.05E-16 |
| TCFL5 | 2.302111 | 2.00E-16 | 9.32E-16 |
| TBC1D24 | 2.119514 | 2.06E-16 | 9.56E-16 |
| RNF165 | 2.557872 | 2.08E-16 | 9.63E-16 |
| SS18L1 | 2.149501 | 2.08E-16 | 9.65E-16 |
| PREX1 | 2.018946 | 2.39E-16 | 1.10E-15 |
| TIAM1 | 2.070769 | 2.45E-16 | 1.13E-15 |
| ZBTB41 | 2.209819 | 2.66E-16 | 1.22E-15 |
| RAMP1 | -2.41932 | 2.83E-16 | 1.30E-15 |
| SPA17 | 2.267192 | 2.92E-16 | 1.34E-15 |
| HOXC4 | 2.390167 | 2.97E-16 | 1.36E-15 |
| SMYD1 | -3.38669 | 3.30E-16 | 1.50E-15 |
| CYP27C1 | 3.032203 | 3.37E-16 | 1.53E-15 |
| PDE4DIP | -3.47835 | 3.39E-16 | 1.54E-15 |
| FAM184A | 2.277846 | 3.64E-16 | 1.65E-15 |
| PLOD2 | 2.98318 | 3.64E-16 | 1.65E-15 |
| ARX | -2.00728 | 3.68E-16 | 1.67E-15 |
| LAMA5 | 2.045672 | 3.73E-16 | 1.69E-15 |
| PUS7 | 2.190876 | 3.82E-16 | 1.73E-15 |
| PLS3 | 2.382155 | 3.87E-16 | 1.76E-15 |
| VANGL2 | 2.259575 | 3.93E-16 | 1.78E-15 |
| NEK2 | 2.386819 | 4.18E-16 | 1.89E-15 |
| LASP1 | 2.029481 | 4.23E-16 | 1.91E-15 |
| CDCA5 | 2.445509 | 5.02E-16 | 2.26E-15 |
| LOC389831 | 2.695862 | 5.22E-16 | 2.35E-15 |
| ZNF704 | 2.095734 | 5.46E-16 | 2.45E-15 |
| COL4A2 | 2.66931 | 6.16E-16 | 2.75E-15 |
| LOC100505501 | 2.66779 | 6.79E-16 | 3.02E-15 |
| CCNE2 | 2.676433 | 7.24E-16 | 3.21E-15 |
| HOTAIRM1 | 2.190857 | 7.39E-16 | 3.27E-15 |
| SGK494 | 2.045561 | 7.44E-16 | 3.29E-15 |
| PALM2 | 3.842366 | 7.73E-16 | 3.42E-15 |
| MFAP2 | 3.409427 | 7.76E-16 | 3.43E-15 |
| POLQ | 2.379906 | 1.08E-15 | 4.70E-15 |
| MYBPC1 | -7.37947 | 1.09E-15 | 4.75E-15 |
| SYNPO2 | -2.68347 | 1.12E-15 | 4.86E-15 |
| ADAM10 | 2.178968 | 1.15E-15 | 5.00E-15 |
| NR2F2-AS1 | 2.218815 | 1.18E-15 | 5.11E-15 |
| FAM134B | -2.07034 | 1.20E-15 | 5.20E-15 |
| MYF6 | -4.65142 | 1.23E-15 | 5.33E-15 |
| CPNE2 | 2.023122 | 1.29E-15 | 5.57E-15 |
| CDC45 | 2.334379 | 1.31E-15 | 5.65E-15 |
| LOC100506563 | -2.04143 | 1.40E-15 | 6.01E-15 |
| NCOA1 | 2.222855 | 1.43E-15 | 6.16E-15 |
| LOC285812 | -2.73689 | 1.59E-15 | 6.82E-15 |
| SLC38A1 | 2.136712 | 1.69E-15 | 7.20E-15 |
| FGFR4 | 2.291153 | 1.70E-15 | 7.25E-15 |
| DUSP27 | -3.67343 | 1.76E-15 | 7.49E-15 |
| DNA2 | 2.528166 | 1.79E-15 | 7.64E-15 |
| HS3ST3A1 | 4.238669 | 1.82E-15 | 7.77E-15 |
| APOD | -3.74274 | 1.87E-15 | 7.95E-15 |
| NIPSNAP3A | 2.095761 | 1.90E-15 | 8.06E-15 |
| MEDAG | 2.088752 | 1.92E-15 | 8.15E-15 |
| ATP10D | 2.54856 | 2.15E-15 | 9.09E-15 |
| EXO1 | 2.629808 | 2.51E-15 | 1.05E-14 |
| FDXR | 2.052527 | 2.86E-15 | 1.20E-14 |
| DACT1 | 3.171174 | 2.92E-15 | 1.22E-14 |
| EMC3-AS1 | 2.037491 | 2.95E-15 | 1.23E-14 |
| SLC7A7 | 2.307293 | 3.10E-15 | 1.29E-14 |
| AURKB | 2.218681 | 3.18E-15 | 1.32E-14 |
| ESF1 | 2.336532 | 3.29E-15 | 1.37E-14 |
| RRAD | -2.28581 | 3.31E-15 | 1.38E-14 |
| SH3BGR | -3.67827 | 3.34E-15 | 1.39E-14 |
| TMEM38B | -2.42066 | 3.47E-15 | 1.44E-14 |
| TTN | -2.83519 | 3.73E-15 | 1.54E-14 |
| ZMAT3 | 2.075355 | 4.09E-15 | 1.69E-14 |
| PRIM1 | 2.170855 | 4.32E-15 | 1.78E-14 |
| CRYAB | -3.9713 | 4.37E-15 | 1.80E-14 |
| DEPDC7 | 3.02409 | 5.16E-15 | 2.11E-14 |
| GPR65 | 2.371147 | 5.18E-15 | 2.12E-14 |
| RARRES2 | 3.073211 | 5.33E-15 | 2.18E-14 |
| PDLIM3 | -2.70847 | 5.50E-15 | 2.24E-14 |
| SLN | -6.46016 | 5.68E-15 | 2.32E-14 |
| DOK5 | -3.04226 | 5.79E-15 | 2.36E-14 |
| TNNC1 | -6.89021 | 5.81E-15 | 2.36E-14 |
| HELB | 2.061277 | 5.86E-15 | 2.38E-14 |
| ME1 | -2.28189 | 6.60E-15 | 2.67E-14 |
| SHROOM2 | 3.134294 | 6.93E-15 | 2.80E-14 |
| TSPAN8 | -4.35478 | 7.82E-15 | 3.14E-14 |
| APOBEC2 | -4.2621 | 8.93E-15 | 3.57E-14 |
| PABPC4L | 3.251804 | 9.20E-15 | 3.67E-14 |
| NMRAL1 | 2.049604 | 9.34E-15 | 3.73E-14 |
| LMOD1 | -2.09535 | 9.66E-15 | 3.85E-14 |
| DUSP6 | 3.313342 | 1.01E-14 | 4.01E-14 |
| NPM3 | 2.05123 | 1.05E-14 | 4.16E-14 |
| MICB | 2.496045 | 1.06E-14 | 4.23E-14 |
| CA3 | -6.39105 | 1.11E-14 | 4.40E-14 |
| C11orf96 | 3.043598 | 1.13E-14 | 4.46E-14 |
| PSPH | 2.015443 | 1.33E-14 | 5.23E-14 |
| SORCS2 | 2.012098 | 1.37E-14 | 5.40E-14 |
| NID2 | 3.205412 | 1.53E-14 | 5.98E-14 |
| PNMA2 | 2.074389 | 1.54E-14 | 6.04E-14 |
| SCRIB | 2.243815 | 1.60E-14 | 6.25E-14 |
| ALCAM | 2.360144 | 1.61E-14 | 6.30E-14 |
| RASSF4 | 2.550968 | 1.62E-14 | 6.30E-14 |
| PVRL3 | 3.03717 | 1.71E-14 | 6.65E-14 |
| ZNF124 | 2.045923 | 1.84E-14 | 7.14E-14 |
| HOMER2 | -2.14437 | 1.91E-14 | 7.39E-14 |
| MYLK4 | -2.7447 | 1.97E-14 | 7.62E-14 |
| CD47 | 2.060171 | 1.98E-14 | 7.64E-14 |
| TDRP | 2.04932 | 2.10E-14 | 8.10E-14 |
| LHX2 | 2.786534 | 2.11E-14 | 8.13E-14 |
| LPAR4 | 3.749099 | 2.43E-14 | 9.33E-14 |
| ATP1B4 | -4.11483 | 2.45E-14 | 9.38E-14 |
| CARNS1 | -2.61501 | 2.52E-14 | 9.67E-14 |
| AB074162 /// MIR181A2HG | 3.299116 | 2.56E-14 | 9.80E-14 |
| EGR1 | 3.219678 | 2.61E-14 | 9.96E-14 |
| SULF1 | 3.082651 | 2.97E-14 | 1.13E-13 |
| COL12A1 | 2.206146 | 3.29E-14 | 1.25E-13 |
| COL6A3 | 2.283938 | 3.42E-14 | 1.29E-13 |
| KANK4 | 5.336996 | 3.72E-14 | 1.40E-13 |
| PGBD5 | 3.358342 | 3.78E-14 | 1.42E-13 |
| TUBB2B | 3.86395 | 3.86E-14 | 1.45E-13 |
| LOC101930415 | 3.069571 | 3.96E-14 | 1.49E-13 |
| FBXO6 | -2.19292 | 4.20E-14 | 1.58E-13 |
| HYMAI | 2.351086 | 4.29E-14 | 1.61E-13 |
| FAM117A | 2.000325 | 4.30E-14 | 1.61E-13 |
| MLIP | -4.38518 | 4.38E-14 | 1.64E-13 |
| LOC728819 | 2.272564 | 4.40E-14 | 1.65E-13 |
| WIPI1 | -2.01802 | 4.50E-14 | 1.68E-13 |
| SGCE | 2.434516 | 4.57E-14 | 1.71E-13 |
| NRCAM | 3.213216 | 5.16E-14 | 1.92E-13 |
| PABPC5 | 2.458001 | 5.30E-14 | 1.97E-13 |
| S100A11 | 2.158312 | 5.68E-14 | 2.11E-13 |
| CABLES1 | 2.245443 | 5.86E-14 | 2.17E-13 |
| ZNF334 | 2.954975 | 6.08E-14 | 2.25E-13 |
| RGS17 | 3.13153 | 6.12E-14 | 2.26E-13 |
| CLSTN2 | 2.863018 | 6.29E-14 | 2.32E-13 |
| PLCD4 | -2.99192 | 6.40E-14 | 2.36E-13 |
| LINC00844 | -2.30406 | 6.69E-14 | 2.46E-13 |
| KIF18B | 2.497746 | 7.22E-14 | 2.65E-13 |
| CSRP3 | -6.19893 | 7.40E-14 | 2.71E-13 |
| ESM1 | 2.530362 | 7.65E-14 | 2.80E-13 |
| AHR | 2.754666 | 7.83E-14 | 2.87E-13 |
| FJX1 | 2.240205 | 8.43E-14 | 3.08E-13 |
| OLFML2A | 2.630307 | 8.46E-14 | 3.09E-13 |
| STC1 | 2.504968 | 8.79E-14 | 3.21E-13 |
| MEIS1 | 2.627538 | 9.92E-14 | 3.60E-13 |
| FRMD6 | 2.70809 | 1.01E-13 | 3.65E-13 |
| CDCA7L | 2.154513 | 1.02E-13 | 3.70E-13 |
| ANK1 | -2.72581 | 1.10E-13 | 3.97E-13 |
| C1orf106 | 3.380036 | 1.10E-13 | 4.00E-13 |
| SYTL2 | 2.187735 | 1.12E-13 | 4.04E-13 |
| RHPN2 | 2.232933 | 1.21E-13 | 4.38E-13 |
| DDX60 | 2.246575 | 1.40E-13 | 5.03E-13 |
| LOC102724927 | 3.000371 | 1.43E-13 | 5.12E-13 |
| IGFBP2 | 3.609473 | 1.59E-13 | 5.68E-13 |
| IP6K3 | -2.70596 | 1.62E-13 | 5.78E-13 |
| FTX | 2.047252 | 1.63E-13 | 5.82E-13 |
| ENOX1 | 2.845312 | 1.66E-13 | 5.91E-13 |
| COPG2IT1 | 4.035075 | 1.68E-13 | 5.99E-13 |
| GLT8D2 | 2.980056 | 1.68E-13 | 5.99E-13 |
| HFE2 | -4.05839 | 1.88E-13 | 6.67E-13 |
| FAT3 | 2.962841 | 1.89E-13 | 6.69E-13 |
| BOK | 2.207012 | 1.96E-13 | 6.94E-13 |
| XK | -2.23427 | 2.09E-13 | 7.37E-13 |
| CEBPB | -2.57176 | 2.15E-13 | 7.61E-13 |
| MEF2C | -2.58457 | 2.18E-13 | 7.71E-13 |
| TMEM200A | 3.039364 | 2.24E-13 | 7.89E-13 |
| NES | 2.46123 | 2.31E-13 | 8.13E-13 |
| THBS2 | 3.844841 | 2.32E-13 | 8.17E-13 |
| MST4 | 3.472717 | 2.33E-13 | 8.19E-13 |
| PEG10 | 4.18197 | 2.42E-13 | 8.52E-13 |
| LOXL3 | 2.028791 | 2.48E-13 | 8.70E-13 |
| CHRNA1 | 3.870145 | 2.64E-13 | 9.25E-13 |
| DCLK1 | 2.918643 | 2.89E-13 | 1.01E-12 |
| SV2A | 2.296445 | 2.93E-13 | 1.02E-12 |
| NR2F1 | 3.606502 | 3.01E-13 | 1.05E-12 |
| NCAPH | 2.110507 | 3.14E-13 | 1.09E-12 |
| ALDH2 | -2.36221 | 3.40E-13 | 1.18E-12 |
| KIAA1598 | 3.277868 | 3.85E-13 | 1.33E-12 |
| PDK4 | -2.94456 | 4.04E-13 | 1.39E-12 |
| GADD45A | 2.056049 | 4.38E-13 | 1.51E-12 |
| NRK | 3.893212 | 4.44E-13 | 1.53E-12 |
| PAG1 | 2.729193 | 4.67E-13 | 1.60E-12 |
| SYT17 | 3.153102 | 4.67E-13 | 1.60E-12 |
| NPNT | 2.576346 | 4.70E-13 | 1.61E-12 |
| MYH7B | -2.52181 | 4.85E-13 | 1.66E-12 |
| CFD | -3.23113 | 5.15E-13 | 1.76E-12 |
| MEG3 | 2.806609 | 5.18E-13 | 1.77E-12 |
| TNFAIP6 | 2.647328 | 5.21E-13 | 1.78E-12 |
| IL21R | 2.098735 | 5.85E-13 | 1.99E-12 |
| MREG | 2.086686 | 5.98E-13 | 2.03E-12 |
| ZNF404 | 2.670624 | 6.03E-13 | 2.05E-12 |
| CDH2 | 2.121188 | 6.14E-13 | 2.08E-12 |
| TGFB1I1 | 2.177707 | 6.28E-13 | 2.12E-12 |
| ELAVL2 | 3.915077 | 6.87E-13 | 2.32E-12 |
| PCDHB2 | 2.758542 | 7.53E-13 | 2.53E-12 |
| LUM | 3.026477 | 7.93E-13 | 2.66E-12 |
| LOX | 2.670469 | 8.04E-13 | 2.70E-12 |
| C1QTNF3 | 2.888766 | 8.44E-13 | 2.83E-12 |
| FOXO6 | 2.007211 | 8.67E-13 | 2.90E-12 |
| QPRT | 2.010364 | 9.07E-13 | 3.03E-12 |
| ERRFI1 | 2.918389 | 9.27E-13 | 3.09E-12 |
| RND3 | 2.601066 | 9.50E-13 | 3.17E-12 |
| CPXM1 | 2.613038 | 9.81E-13 | 3.27E-12 |
| DLGAP1-AS2 | 2.094962 | 1.01E-12 | 3.36E-12 |
| FAM169A | 2.490172 | 1.05E-12 | 3.50E-12 |
| LINC00883 | 2.640944 | 1.08E-12 | 3.57E-12 |
| LOXL1 | 2.673693 | 1.16E-12 | 3.83E-12 |
| CDK8 | 2.433918 | 1.18E-12 | 3.89E-12 |
| LIPA | 2.079769 | 1.26E-12 | 4.16E-12 |
| PRR16 | -2.57828 | 1.34E-12 | 4.39E-12 |
| PHLDA1 | 2.080971 | 1.35E-12 | 4.44E-12 |
| MAGEL2 | 2.843132 | 1.42E-12 | 4.66E-12 |
| LOC101927720 | 2.234299 | 1.47E-12 | 4.81E-12 |
| IGSF5 | 2.893928 | 1.48E-12 | 4.85E-12 |
| LPPR1 | 2.201183 | 1.54E-12 | 5.05E-12 |
| HENMT1 | 2.12768 | 1.55E-12 | 5.06E-12 |
| SEL1L3 | 2.202605 | 1.59E-12 | 5.20E-12 |
| TTTY15 | 2.429637 | 1.75E-12 | 5.70E-12 |
| HSPA4L | 2.344519 | 1.75E-12 | 5.71E-12 |
| ZNF106 | -2.08917 | 1.78E-12 | 5.79E-12 |
| RGS4 | 2.393487 | 1.83E-12 | 5.94E-12 |
| BNC2 | 2.059216 | 1.89E-12 | 6.11E-12 |
| TOX | 2.008027 | 1.91E-12 | 6.19E-12 |
| MGC12488 | 2.261482 | 1.95E-12 | 6.31E-12 |
| GJA1 | 2.735151 | 2.13E-12 | 6.86E-12 |
| TGFBI | 2.594067 | 2.17E-12 | 7.00E-12 |
| STOX2 | 2.075167 | 2.25E-12 | 7.23E-12 |
| TAS2R10 | 2.092273 | 2.30E-12 | 7.38E-12 |
| AGPAT9 | -2.75224 | 2.33E-12 | 7.48E-12 |
| NKAIN4 | 2.938902 | 2.62E-12 | 8.37E-12 |
| C14orf132 | 2.278356 | 2.62E-12 | 8.38E-12 |
| USP18 | 2.709306 | 2.74E-12 | 8.73E-12 |
| SP5 | 2.365828 | 3.00E-12 | 9.56E-12 |
| PMAIP1 | 3.107991 | 3.41E-12 | 1.08E-11 |
| SLC19A2 | -2.09191 | 3.45E-12 | 1.09E-11 |
| SLC2A3 | 2.375063 | 3.54E-12 | 1.12E-11 |
| KIAA0226L | 2.912627 | 3.58E-12 | 1.13E-11 |
| ITGB1BP2 | -2.55797 | 3.69E-12 | 1.16E-11 |
| SMPX | -5.02919 | 3.93E-12 | 1.24E-11 |
| ENPP5 | -2.4378 | 4.47E-12 | 1.40E-11 |
| FLJ45482 | 2.026365 | 4.75E-12 | 1.49E-11 |
| HES6 | 2.924756 | 5.33E-12 | 1.66E-11 |
| ZNF300 | 3.134361 | 5.37E-12 | 1.67E-11 |
| ACTA1 | -5.76237 | 5.60E-12 | 1.74E-11 |
| MYOZ2 | -4.02774 | 5.61E-12 | 1.74E-11 |
| RNF128 | -2.75145 | 6.30E-12 | 1.95E-11 |
| STARD4 | 2.194135 | 6.57E-12 | 2.03E-11 |
| KRT80 | 2.553631 | 6.84E-12 | 2.11E-11 |
| SYNPO2L | -3.61859 | 7.40E-12 | 2.27E-11 |
| ST6GAL2 | 3.307076 | 7.65E-12 | 2.35E-11 |
| CACNA2D1 | -2.47213 | 7.69E-12 | 2.36E-11 |
| DDX25 | 2.021301 | 7.91E-12 | 2.42E-11 |
| LRP8 | 2.054533 | 1.07E-11 | 3.23E-11 |
| TUFT1 | 2.058146 | 1.10E-11 | 3.33E-11 |
| GRIP1 | 2.084498 | 1.17E-11 | 3.54E-11 |
| CITED1 | 2.310624 | 1.20E-11 | 3.62E-11 |
| EPHA7 | 2.555224 | 1.32E-11 | 3.97E-11 |
| ABCA5 | -2.07607 | 1.39E-11 | 4.18E-11 |
| ENPP4 | -2.3542 | 1.42E-11 | 4.25E-11 |
| TMTC2 | 2.627164 | 1.47E-11 | 4.41E-11 |
| RCSD1 | -2.21362 | 1.48E-11 | 4.45E-11 |
| TNC | 2.552667 | 1.51E-11 | 4.53E-11 |
| TCEAL7 | 3.018343 | 1.53E-11 | 4.59E-11 |
| CRNDE | 2.016076 | 1.60E-11 | 4.79E-11 |
| FLJ41170 | 2.116422 | 1.66E-11 | 4.97E-11 |
| KCNJ2 | -2.08026 | 1.71E-11 | 5.10E-11 |
| ANKS1A | 2.080185 | 1.72E-11 | 5.12E-11 |
| PCOLCE | 2.556038 | 1.80E-11 | 5.35E-11 |
| SYNC | -2.81222 | 1.85E-11 | 5.49E-11 |
| CNN3 | 2.084208 | 2.02E-11 | 6.00E-11 |
| ALPK2 | -2.95114 | 2.06E-11 | 6.11E-11 |
| IFI16 | 2.374219 | 2.16E-11 | 6.40E-11 |
| TET1 | 2.412947 | 2.18E-11 | 6.45E-11 |
| DCX | 5.327581 | 2.31E-11 | 6.79E-11 |
| COL21A1 | 2.842076 | 2.61E-11 | 7.66E-11 |
| KCNT2 | 3.34151 | 2.75E-11 | 8.05E-11 |
| AC010980.2 /// LOC440934 /// LOC440934 | 2.585124 | 2.94E-11 | 8.59E-11 |
| TOX3 | 3.666985 | 2.98E-11 | 8.70E-11 |
| RAP1GAP2 | 3.777049 | 3.72E-11 | 1.08E-10 |
| SPON1 | 2.367878 | 3.74E-11 | 1.08E-10 |
| CYFIP2 | 2.102035 | 3.75E-11 | 1.08E-10 |
| BEX4 | 2.575857 | 3.76E-11 | 1.09E-10 |
| SAMD9 | 2.078861 | 3.82E-11 | 1.10E-10 |
| DNM3OS | 3.214769 | 4.22E-11 | 1.22E-10 |
| PCDHGA4 | 2.634382 | 4.33E-11 | 1.25E-10 |
| TMEM133 | 2.143428 | 4.49E-11 | 1.29E-10 |
| PCDH17 | 3.239763 | 4.75E-11 | 1.37E-10 |
| LOC101929504 | 2.297764 | 5.24E-11 | 1.50E-10 |
| EDNRA | 2.444433 | 5.92E-11 | 1.69E-10 |
| MS4A7 | 2.423945 | 6.12E-11 | 1.74E-10 |
| PCDH8 | 4.144587 | 6.50E-11 | 1.85E-10 |
| C5AR1 | 2.194804 | 6.97E-11 | 1.98E-10 |
| PPM1E | 2.144304 | 7.40E-11 | 2.09E-10 |
| CYR61 | 2.701672 | 8.09E-11 | 2.28E-10 |
| HIST2H2BE | -2.25978 | 8.26E-11 | 2.33E-10 |
| LGR5 | -2.63201 | 8.35E-11 | 2.35E-10 |
| TNNC2 | -5.54193 | 8.46E-11 | 2.38E-10 |
| IGFBPL1 | 2.673944 | 8.48E-11 | 2.39E-10 |
| MAN1C1 | 2.071097 | 9.07E-11 | 2.54E-10 |
| CDC42EP5 | 2.670372 | 9.58E-11 | 2.68E-10 |
| IGFBP3 | 2.725584 | 9.82E-11 | 2.74E-10 |
| COL11A1 | 3.60312 | 1.07E-10 | 2.98E-10 |
| TNNI1 | -4.35425 | 1.09E-10 | 3.03E-10 |
| TMEM132D | 2.642196 | 1.16E-10 | 3.22E-10 |
| NEFH | 2.11747 | 1.45E-10 | 4.01E-10 |
| PLAT | 2.282337 | 1.47E-10 | 4.06E-10 |
| ST6GALNAC5 | 2.167102 | 1.51E-10 | 4.15E-10 |
| TOX2 | 2.844962 | 1.62E-10 | 4.46E-10 |
| LAMA1 | 2.315483 | 1.63E-10 | 4.47E-10 |
| FLJ38379 | 2.598865 | 1.66E-10 | 4.54E-10 |
| ZNF536 | 3.492738 | 1.72E-10 | 4.72E-10 |
| LAPTM5 | 2.023493 | 1.98E-10 | 5.40E-10 |
| DPY19L2 | 3.195054 | 2.16E-10 | 5.85E-10 |
| PDLIM1 | -2.07434 | 2.33E-10 | 6.30E-10 |
| KLHL41 | -3.77109 | 2.39E-10 | 6.48E-10 |
| GPX3 | -3.04188 | 2.42E-10 | 6.55E-10 |
| COL2A1 | 4.168095 | 2.46E-10 | 6.64E-10 |
| C3orf70 | 2.818891 | 2.48E-10 | 6.69E-10 |
| SDC1 | 2.26535 | 2.74E-10 | 7.37E-10 |
| HOXA10 | -2.22412 | 2.85E-10 | 7.66E-10 |
| TNNT2 | 2.175617 | 2.96E-10 | 7.93E-10 |
| LOC729680 | -2.37573 | 3.01E-10 | 8.06E-10 |
| GPC3 | 2.251427 | 3.12E-10 | 8.35E-10 |
| GABRB3 | 2.200042 | 3.17E-10 | 8.50E-10 |
| LOC101060391 | 4.285343 | 3.24E-10 | 8.66E-10 |
| FNBP1L | 2.159595 | 3.35E-10 | 8.94E-10 |
| NLGN4X | 2.61225 | 3.54E-10 | 9.43E-10 |
| SYDE2 | 2.542099 | 3.70E-10 | 9.86E-10 |
| GHR | -2.83934 | 3.81E-10 | 1.01E-09 |
| BTBD3 | 2.103627 | 3.83E-10 | 1.02E-09 |
| DISP1 | 2.137998 | 3.97E-10 | 1.06E-09 |
| ISL1 | 3.979703 | 4.12E-10 | 1.09E-09 |
| LY96 | 2.350371 | 4.14E-10 | 1.10E-09 |
| RASD1 | 2.358469 | 4.50E-10 | 1.19E-09 |
| MSC | 2.005404 | 4.76E-10 | 1.25E-09 |
| C2orf40 | -2.31943 | 4.94E-10 | 1.30E-09 |
| LGI1 | -2.42631 | 5.03E-10 | 1.32E-09 |
| MAP2 | 2.655904 | 5.19E-10 | 1.36E-09 |
| DMRTA2 | 2.386162 | 5.59E-10 | 1.46E-09 |
| PCSK5 | 2.19592 | 5.66E-10 | 1.48E-09 |
| PBX4 | 2.397956 | 5.79E-10 | 1.52E-09 |
| ALDH1A1 | -2.76446 | 5.90E-10 | 1.54E-09 |
| LOC284219 | 2.057441 | 6.00E-10 | 1.57E-09 |
| TAGLN3 | 2.40921 | 6.08E-10 | 1.59E-09 |
| PROM1 | 3.882131 | 6.62E-10 | 1.72E-09 |
| PCDH19 | 2.122559 | 7.15E-10 | 1.86E-09 |
| ST8SIA1 | 2.150215 | 7.31E-10 | 1.90E-09 |
| HTRA1 | 2.127542 | 7.65E-10 | 1.98E-09 |
| C1orf105 | 3.879209 | 7.69E-10 | 1.99E-09 |
| B3GALT2 | 2.660126 | 7.88E-10 | 2.04E-09 |
| FBN3 | 2.399796 | 8.08E-10 | 2.09E-09 |
| PLK2 | 2.400256 | 8.09E-10 | 2.09E-09 |
| APOE | 2.087775 | 8.29E-10 | 2.14E-09 |
| VGLL2 | -2.50762 | 8.41E-10 | 2.17E-09 |
| GPR183 | 2.097723 | 8.72E-10 | 2.25E-09 |
| EGR3 | 2.625971 | 9.22E-10 | 2.37E-09 |
| TNNT1 | -4.13075 | 9.68E-10 | 2.48E-09 |
| DUXAP10 | 3.040926 | 1.22E-09 | 3.10E-09 |
| ALDH1A3 | 2.421934 | 1.26E-09 | 3.21E-09 |
| RNF182 | 2.808948 | 1.29E-09 | 3.29E-09 |
| NEB | -2.83976 | 1.32E-09 | 3.36E-09 |
| MRAP2 | 2.420899 | 1.49E-09 | 3.76E-09 |
| TSPAN12 | 2.464308 | 1.49E-09 | 3.76E-09 |
| CD24 | 2.428482 | 1.49E-09 | 3.77E-09 |
| LOC100131541 | 2.171292 | 1.49E-09 | 3.78E-09 |
| CDH3 | 2.482581 | 1.50E-09 | 3.79E-09 |
| ADRB2 | -2.22934 | 1.69E-09 | 4.27E-09 |
| MYOD1 | 2.380212 | 1.79E-09 | 4.51E-09 |
| PDZD2 | 2.130972 | 1.80E-09 | 4.52E-09 |
| GPC6 | 2.134441 | 1.87E-09 | 4.70E-09 |
| PITPNM3 | 2.233893 | 2.00E-09 | 5.01E-09 |
| TOM1L1 | 2.475308 | 2.04E-09 | 5.12E-09 |
| CD36 | -2.27378 | 2.17E-09 | 5.42E-09 |
| GBP1 | 2.294872 | 2.20E-09 | 5.48E-09 |
| TMEM132C | 2.095777 | 2.26E-09 | 5.62E-09 |
| APOBEC3B | 2.273489 | 2.53E-09 | 6.29E-09 |
| LINC00472 | 2.042101 | 2.61E-09 | 6.47E-09 |
| CA2 | -2.66254 | 2.84E-09 | 7.02E-09 |
| LPL | -2.79342 | 3.29E-09 | 8.08E-09 |
| ISG15 | 2.292393 | 3.80E-09 | 9.28E-09 |
| SPINK5 | 2.019309 | 3.84E-09 | 9.38E-09 |
| FIBIN | 2.072408 | 4.00E-09 | 9.77E-09 |
| IRX1 | 3.114038 | 4.05E-09 | 9.87E-09 |
| GNAI1 | 2.156853 | 4.15E-09 | 1.01E-08 |
| KLHL13 | 2.573747 | 4.27E-09 | 1.04E-08 |
| PKIA | -2.85979 | 4.52E-09 | 1.10E-08 |
| MTUS2 | 2.118393 | 4.54E-09 | 1.10E-08 |
| LINC00948 | -3.94336 | 4.59E-09 | 1.11E-08 |
| NRTN | 3.564188 | 4.84E-09 | 1.17E-08 |
| JAKMIP2 | 3.000792 | 5.69E-09 | 1.37E-08 |
| TEX14 | 3.279089 | 5.79E-09 | 1.39E-08 |
| LOC400043 | 2.351044 | 5.79E-09 | 1.39E-08 |
| MYLPF | -4.59168 | 6.30E-09 | 1.51E-08 |
| DMRT2 | 2.064253 | 6.35E-09 | 1.52E-08 |
| IGF2BP3 | 2.167624 | 6.99E-09 | 1.67E-08 |
| VCAM1 | 2.755924 | 7.81E-09 | 1.86E-08 |
| C1QB | 2.265409 | 7.84E-09 | 1.86E-08 |
| RGL1 | 2.017776 | 8.03E-09 | 1.91E-08 |
| LINC00622 | 2.258513 | 8.05E-09 | 1.91E-08 |
| SPRY4 | 2.137814 | 8.30E-09 | 1.97E-08 |
| HERC5 | 2.626509 | 8.91E-09 | 2.11E-08 |
| CRISPLD1 | 3.200834 | 1.03E-08 | 2.43E-08 |
| PCDHB5 | 2.466265 | 1.17E-08 | 2.75E-08 |
| CTGF | 2.3472 | 1.18E-08 | 2.76E-08 |
| IFI44 | 2.112248 | 1.18E-08 | 2.77E-08 |
| DUSP4 | 2.539546 | 1.19E-08 | 2.79E-08 |
| EYA2 | 2.776409 | 1.42E-08 | 3.31E-08 |
| NFKBIZ | 2.01856 | 1.58E-08 | 3.68E-08 |
| NMU | 2.64436 | 1.67E-08 | 3.87E-08 |
| CADM2 | 2.738294 | 1.71E-08 | 3.95E-08 |
| TMEM119 | 2.339971 | 1.71E-08 | 3.97E-08 |
| MT1X | -2.06376 | 1.80E-08 | 4.16E-08 |
| LINC00545 | 2.999951 | 1.81E-08 | 4.19E-08 |
| HSPB3 | -3.55637 | 2.49E-08 | 5.69E-08 |
| ZNF385B | -2.42399 | 2.76E-08 | 6.27E-08 |
| MURC | -2.80157 | 2.85E-08 | 6.47E-08 |
| CAPN6 | 2.939404 | 3.21E-08 | 7.28E-08 |
| ZNF804A | 2.918196 | 3.35E-08 | 7.58E-08 |
| TNFSF13B | 2.168053 | 3.63E-08 | 8.18E-08 |
| RAPGEF4 | 2.317138 | 3.65E-08 | 8.23E-08 |
| IL17B | 2.533067 | 3.73E-08 | 8.41E-08 |
| LIPG | 3.151086 | 4.16E-08 | 9.34E-08 |
| MYL1 | -4.90451 | 4.27E-08 | 9.58E-08 |
| FOXF1 | 3.421535 | 4.73E-08 | 1.05E-07 |
| CD14 | 2.302357 | 4.74E-08 | 1.06E-07 |
| DSP | 2.393992 | 4.88E-08 | 1.09E-07 |
| ADRA2A | 2.856855 | 5.01E-08 | 1.11E-07 |
| PCP4 | 3.493214 | 5.01E-08 | 1.11E-07 |
| TRIM71 | 2.004786 | 5.04E-08 | 1.12E-07 |
| NETO2 | 2.21065 | 5.11E-08 | 1.14E-07 |
| TSPAN7 | -2.07194 | 5.26E-08 | 1.17E-07 |
| HOOK1 | 2.154122 | 5.33E-08 | 1.18E-07 |
| LINC00588 | 3.228 | 5.59E-08 | 1.24E-07 |
| HRASLS | -2.31198 | 5.59E-08 | 1.24E-07 |
| EN2 | 2.33837 | 6.08E-08 | 1.34E-07 |
| LRFN5 | 2.60037 | 6.40E-08 | 1.41E-07 |
| ASB5 | -4.1292 | 7.28E-08 | 1.60E-07 |
| ANKRD6 | 2.040492 | 8.35E-08 | 1.82E-07 |
| FHOD3 | -2.47215 | 9.08E-08 | 1.98E-07 |
| EDN3 | 2.654079 | 9.42E-08 | 2.05E-07 |
| FST | 2.257601 | 9.55E-08 | 2.07E-07 |
| TFAP2B | 3.529598 | 1.40E-07 | 3.00E-07 |
| FOXD1 | 2.571055 | 1.47E-07 | 3.14E-07 |
| SATB2 | 2.0613 | 1.48E-07 | 3.17E-07 |
| NTN4 | 2.002815 | 1.89E-07 | 4.01E-07 |
| TWIST1 | 2.560388 | 1.94E-07 | 4.11E-07 |
| FAM129A | -2.06165 | 3.19E-07 | 6.64E-07 |
| ANKRD18A | 2.054767 | 3.64E-07 | 7.54E-07 |
| MYL4 | 2.465789 | 3.82E-07 | 7.90E-07 |
| C1QC | 2.05195 | 4.07E-07 | 8.40E-07 |
| RCAN2 | -2.04743 | 4.16E-07 | 8.57E-07 |
| CCND1 | 2.07902 | 4.54E-07 | 9.33E-07 |
| FOSB | 2.418766 | 4.79E-07 | 9.82E-07 |
| EYA1 | 2.28245 | 5.08E-07 | 1.04E-06 |
| SLC47A1 | -2.03269 | 5.38E-07 | 1.10E-06 |
| PPP1R14C | -2.27156 | 5.43E-07 | 1.11E-06 |
| NLGN1 | 2.243751 | 5.80E-07 | 1.18E-06 |
| COL10A1 | 2.251738 | 6.35E-07 | 1.29E-06 |
| LRRN1 | 2.713262 | 8.22E-07 | 1.65E-06 |
| LRRN3 | 2.31632 | 9.33E-07 | 1.86E-06 |
| MYH3 | 3.812723 | 9.36E-07 | 1.87E-06 |
| CASQ2 | -3.01205 | 1.17E-06 | 2.31E-06 |
| WNT5A | 2.270764 | 1.18E-06 | 2.33E-06 |
| SGMS1 | 2.242824 | 1.36E-06 | 2.68E-06 |
| INSM1 | 2.481627 | 1.53E-06 | 3.00E-06 |
| POU4F1 | 3.531874 | 1.63E-06 | 3.19E-06 |
| ADAMDEC1 | 2.028258 | 1.77E-06 | 3.45E-06 |
| HOTAIR | 2.856451 | 2.19E-06 | 4.24E-06 |
| MGP | 2.025162 | 2.23E-06 | 4.30E-06 |
| HMCN1 | 2.655477 | 2.30E-06 | 4.43E-06 |
| CNR1 | 2.18331 | 2.45E-06 | 4.71E-06 |
| FOS | 2.626663 | 2.54E-06 | 4.88E-06 |
| PIPOX | 3.563336 | 2.67E-06 | 5.11E-06 |
| ENPP6 | 2.955644 | 2.91E-06 | 5.56E-06 |
| RASSF10 | 2.825726 | 4.82E-06 | 9.02E-06 |
| OTX2 | 2.912435 | 4.85E-06 | 9.07E-06 |
| CYTL1 | 2.474885 | 5.12E-06 | 9.56E-06 |
| UNC45B | -2.85758 | 5.51E-06 | 1.03E-05 |
| ASS1 | 2.925297 | 6.65E-06 | 1.23E-05 |
| SCG2 | 2.08847 | 6.85E-06 | 1.26E-05 |
| ADIPOQ | -2.23063 | 6.99E-06 | 1.29E-05 |
| CCL8 | 2.199397 | 9.71E-06 | 1.77E-05 |
| RYR1 | -2.00779 | 1.00E-05 | 1.83E-05 |
| CBLN2 | 2.168966 | 1.31E-05 | 2.36E-05 |
| FGF9 | 2.117074 | 2.95E-05 | 5.13E-05 |
| DNER | 2.354625 | 3.02E-05 | 5.25E-05 |
| NPY | 2.834706 | 3.06E-05 | 5.31E-05 |
| BCHE | 2.602866 | 3.29E-05 | 5.69E-05 |
| SFRP2 | 2.694386 | 3.63E-05 | 6.25E-05 |
| LOC285556 | -2.45406 | 4.12E-05 | 7.07E-05 |
| CHODL | 2.211741 | 4.80E-05 | 8.18E-05 |
| ACTC1 | 2.148377 | 4.88E-05 | 8.32E-05 |
| CNTN3 | 2.642254 | 6.02E-05 | 0.000102 |
| SORCS1 | 2.124882 | 9.03E-05 | 0.00015 |
| PRAME | 2.432555 | 0.00014 | 0.000229 |
| MPPED2 | 2.106916 | 0.000142 | 0.000231 |
| IGJ | 2.136251 | 0.000162 | 0.000263 |
| MSTN | 2.051024 | 0.000377 | 0.00059 |
| FCRLA | 2.038808 | 0.000598 | 0.000917 |
| DSCR8 | 2.072656 | 0.001498 | 0.002217 |
| NNAT | 2.032479 | 0.002606 | 0.003763 |
| MYH8 | 2.057657 | 0.004701 | 0.006597 |
| RPS4Y1 | 2.572863 | 0.005973 | 0.008292 |
