# Supplementary figures and images for: CDK1 and CCNB1 as potential diagnostic markers of rhabdomyosarcoma: validation following bioinformatics analysis
Source: BMC Med Genomics. 2019 Dec 23;12:198. doi: 10.1186/s12920-019-0645-x (PMC6929508; doi:10.1186/s12920-019-0645-x)

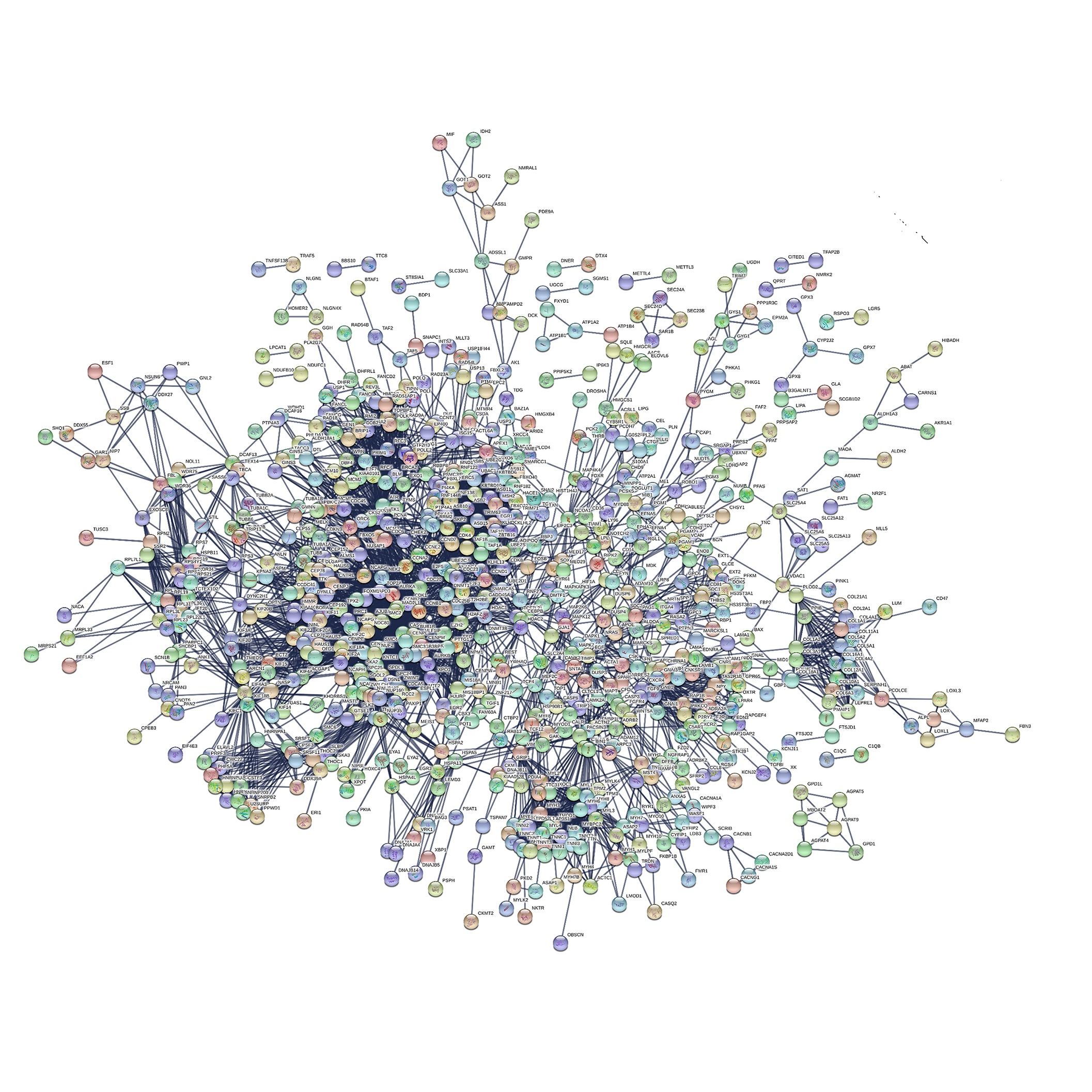

Supplement: Supplementary file 3 — Additional file 3: Figure S1. PPI network of DEGs. Each node expresses a protein, and each line represents the relationship of proteins to each other. Nodes of different colors have different score values. [file 12920_2019_645_MOESM3_ESM.png]
